# Inhibition of [FeFe]-hydrogenase by formaldehyde: proposed mechanism and reactivity of FeFe alkyl complexes†

**DOI:** 10.1039/d1sc05803g

**Published:** 2021-11-16

**Authors:** Fanjun Zhang, Toby J. Woods, Lingyang Zhu, Thomas B. Rauchfuss

**Affiliations:** School of Chemical Sciences, University of Illinois at Urbana-Champaign Urbana IL 61801 USA rauchfuz@illinois.edu

## Abstract

The mechanism for inhibition of [FeFe]-hydrogenases by formaldehyde is examined with model complexes. Key findings: (i) CH_2_ donated by formaldehyde covalently link Fe and the amine cofactor, blocking the active site and (ii) the resulting Fe-alkyl is a versatile electrophilic alkylating agent. Solutions of Fe_2_[(μ-SCH_2_)_2_NH](CO)_4_(PMe_3_)_2_ (1) react with a mixture of HBF_4_ and CH_2_O to give three isomers of [Fe_2_[(μ-SCH_2_)_2_NCH_2_](CO)_4_(PMe_3_)_2_]^+^ ([2]^+^). X-ray crystallography verified the NCH_2_Fe linkage to an octahedral Fe(ii) site. Although [2]^+^ is stereochemically rigid on the NMR timescale, spin-saturation transfer experiments implicate reversible dissociation of the Fe–CH_2_ bond, allowing interchange of all three diastereoisomers. Using ^13^CH_2_O, the methylenation begins with formation of [Fe_2_[(μ-SCH_2_)_2_N^13^CH_2_OH](CO)_4_(PMe_3_)_2_]^+^. Protonation converts this hydroxymethyl derivative to [2]^+^, concomitant with ^13^C-labelling of all three methylene groups. The Fe–CH_2_N bond in [2]^+^ is electrophilic: PPh_3_, hydroxide, and hydride give, respectively, the phosphonium [Fe_2_[(μ-SCH_2_)_2_NCH_2_PPh_3_](CO)_4_(PMe_3_)_2_]^+^, 1, and the methylamine Fe_2_[(μ-SCH_2_)_2_NCH_3_](CO)_4_(PMe_3_)_2_. The reaction of [Fe_2_[(μ-SCH_2_)_2_NH](CN)_2_(CO)_4_]^2−^ with CH_2_O/HBF_4_ gave [Fe_2_[(μ-SCH_2_)_2_NCH_2_CN](CN)(CO)_5_]^−^ ([4]^−^), the result of reductive elimination from [Fe_2_[(μ-SCH_2_)_2_NCH_2_](CN)_2_(CO)_4_]^−^. The phosphine derivative [Fe_2_[(μ-SCH_2_)_2_NCH_2_CN](CN)(CO)_4_(PPh_3_)]^−^ ([5]^−^) was characterized crystallographically.

## Abstract 2

The mechanism for inhibition of [FeFe]-hydrogenases by formaldehyde is examined with model complexes.

## Graphical abstract

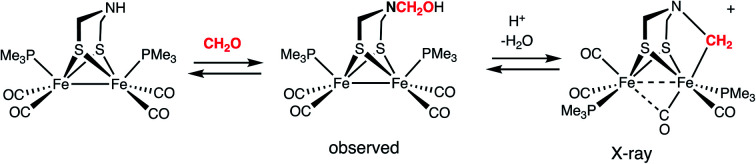

## Introduction

**Fig. 1:**
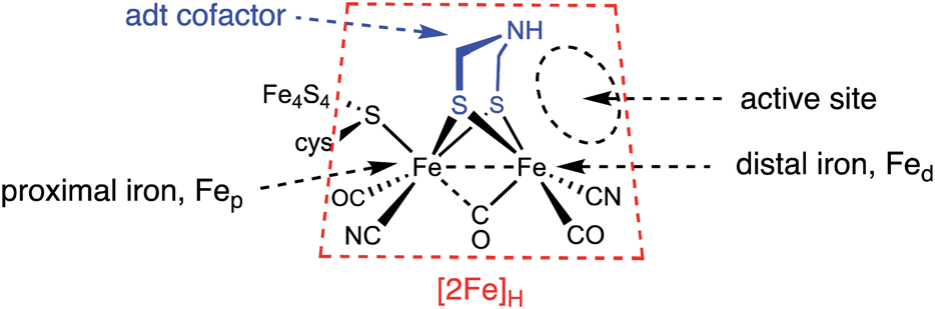
Nomenclature for the [2Fe]_H_ active site of the [FeFe]-hydrogenases.

The hydrogenase enzymes attract attention because they are extremely efficient catalysts for the production and utilization of hydrogen.^1–4^ These enzymes operate by an orchestration of protonations and electron transfers with the substrates being bound in a pocket that, at least in its H_ox_ state, is a Frustrated Lewis Pair (FLP). These components are illustrated in Fig. 1.

Since these catalysts are based on iron, the most earth-abundant transition metal,^5^ Nature's designs promise to inspire to new synthetic catalysts that exhibit the enzyme-like activity but with more convenient molecular weight and air-sensitivity.

Given the significance of the [FeFe]-hydrogenases, many methods have been applied to elucidating their mechanism.^6–10^ One powerful mechanistic probe involves the use of inhibitors. Researchers from Bochum and Oxford described the reversible inhibition of the [FeFe]-hydrogenase from *Clostridium acetobutylicum* and *Desulfovibrio desulfuricans* with formaldehyde.^11,12^ The inhibited state was subsequently characterized for the spectroscopically simpler enzyme from *Chlamydomonas reinhardtii*.^13^ Spin resonance measurements were enabled by the presence of the *S* = ½ [4Fe–4S]^+^ center and augmented by the use of ^13^CH_2_O. Reversible inhibition is unequivocal, the molecular details of the inhibition remain uncertain. One important clue is that inhibition occurs for the reduced states of the enzyme, the oxidized states are less affected.

**Scheme 1:**
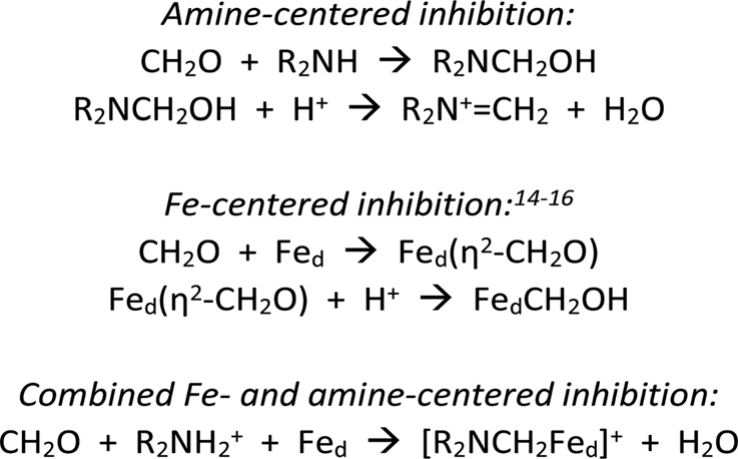
Hypotheses for inhibition of [FeFe]-hydrogenases by CH_2_O.

Scheme 1 summarizes some scenarios for the binding of CH_2_O at [2Fe]_H_.

The amine pathways, which involve standard organic reactions, might be relevant to what Bachmeier *et al.* refer to as “matrix” formaldehyde, *i.e.*, unselective binding of formaldehyde in the vicinity of the active site. The Fe-centered reactions are precedented in organometallic chemistry, although not necessarily with iron. Bachmeier *et al.* favor this hypothesis. The third pathway, for which we provide evidence, involves covalent linking the amine and distal iron with a methylene bridge, locking up the [2Fe]_H_ active site.

The model complexes used in this paper are Fe_2_[(μ-SCH_2_)_2_NH](CO)_4_L_2_, where L = PMe_3_ and CN^−^. These models feature the authentic azadithiolate cofactor bound to a pair of Fe(CO)_2_L centers. Such complexes are functional models in that they undergo protonation to give hydrides and are redox-active.^17^

## Results and discussion

### [Fe_2_[(μ-SCH_2_)_2_NCH_2_](CO)_4_(PMe_3_)_2_]^+^

Solutions of Fe_2_[(μ-SCH_2_)_2_NH](CO)_4_(PMe_3_)_2_ (1) were found to react with a mixture of HBF_4_ and paraformaldehyde to give [Fe_2_[(μ-SCH_2_)_2_NCH_2_](CO)_4_(PMe_3_)_2_]^+^ ([2]^+^). Using stoichiometric amounts of the three reagents, the conversion proceeds rapidly and in good yields at room temperature. These conditions are compatible with those reported for the enzyme. The formula of [2]^+^ was initially determined by ESI-MS, which showed a strong parent ion (eqn (1)).1
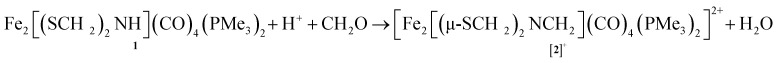


In a control experiment, the reaction of the propanedithiolate Fe_2_(μ-S_2_C_3_H_6_)(CO)_4_(PMe_3_)_2_ with HBF_4_ and paraformaldehyde afforded only the well-known hydride [HFe_2_(μ-S_2_C_3_H_6_)(CO)_4_(PMe_3_)_2_]^+^;^18^ the formaldehyde had no effect.

**Fig. 2:**
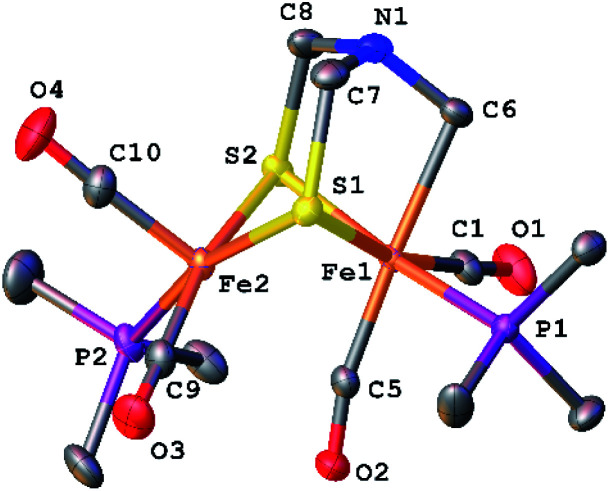
Structure of [Fe_2_[(μ-SCH_2_)_2_NCH_2_](CO)_4_(PMe_3_)_2_]BAr^F^_4_ ([2]BAr^F^_4_) with thermal ellipsoids shown at 50% probability. H atoms and BAr^F^_4_^−^ have been omitted for clarity. Selected distances and angles (Å and °): Fe1–Fe2, 2.5934(3); Fe1–C5, 1.796(2); Fe2–C5, 2.559(2); Fe1–C6, 2.168(2); N1–C6, 1.441(2); N1–C7, 1.431(3); N1–C8, 1.432(2); Fe1–C5–O2, 167.8(2); Fe1–C1–O1, 177.9(2).

The structure of [2]^+^ was determined by an X-ray crystallographic study of its BAr^F^_4_^−^ salt (Ar^F^ = C_6_H_3_-3,5-(CF_3_)_2_) (Fig. 2). The two PMe_3_ ligands are *trans*-dibasal. Fe1 has an S_2_(CO)_2_(PMe_3_)(alkyl) coordination sphere. The ligand–Fe1–ligand angles are suitable for octahedral coordination, as appropriate for Fe(ii). The proximal Fe center Fe2 occupies a S_2_(CO)_2_(PMe_3_) coordination sphere. Its coordination number is ambiguous because one CO, primarily bound to Fe1, is semi-bridging: Fe1–CO = 1.796(2) and Fe2–CO = 2.559(2) Å. The Fe1–C5–O2 angle for the semi-bridging CO ligand is 167.8(2)°, suggesting that Fe2 is weakly Lewis acidic. Analogous to terminal hydride derivatives of Fe_2_^II^(μ-SR)_2_ complexes,^19,20^ the ligand *trans* to alkyl is CO. In a related thioaldehyde complexes^21^ Fe_2_(μ-SR)(μ-η^2^-SCHR′)(diphosphine)(CO)_4_, CO is also *trans* to alkyl.^22^

The NMR data for [2]^+^ are consistent with a stereo-rigid, chiral structure. For example, the ^13^C NMR spectrum shows four CO signals, three signals in the *δ* 211.45–210.70 region assigned to terminal CO groups, and one signal at *δ* 201.8 assigned to the semi-bridging CO. These ^13^CO signals are all coupled to ^31^P (*J*_PC_ = 19.7 Hz), characteristic of CO *cis* to PMe_3_.^23^ The ^13^C NMR signal for the formaldehyde-derived methylene appears at *δ* 75.77. Its ^1^H NMR spectrum shows multiplets at *δ* 5.52 and 4.79, assigned to the diastereotopic CH_2_ protons. The four SCH_2_N protons are nonequivalent, also consistent with the low symmetry of the complex. Spin-saturation transfer experiments, which probes the exchange of signals at rates faster than 1/*T*_1_, were conducted on [2]^+^. Saturation of one of the SCH_2_ signals centered at *δ* 3.84 (*T*_1_ = 1.17 s for SCH_2_) or one of the NCH_2_Fe signals at *δ* 4.79 (*T*_1_ = 1.31 s for NCH_2_Fe) revealed that these sites do not exchange on the seconds time scale (Fig. S14 and S15†). As discussed below, ^13^C-labeling reveals that all three methylene groups do in fact exchange over the course of several minutes.

The ^31^P NMR data for [2]^+^ reveal the presence of a mixture of three (chiral) diastereoisomers in a 5 : 1 : 1 ratio. The minor diastereomers are not evident in the above-discussed ^13^C NMR data. If we assume that Fe1 center has CO *trans* to the alkyl ligand, as mentioned above, the three isomers result from the three diastereomeric sites on Fe2:

We assume that the main isomer (A) has *trans*-dibasal phosphine ligands as established by X-ray crystallography. This dominant and one minor isomer (B) both show ^31^P–^31^P coupling (respectively, *J* = 7.4, 7.6 Hz). The two ^31^P NMR signals for the third isomer (minor, C) show no ^31^P, ^31^P coupling. Its unique (non)coupling is consistent with a unique structure, *i.e.*, apical–basal disposition of the PMe_3_ ligands.
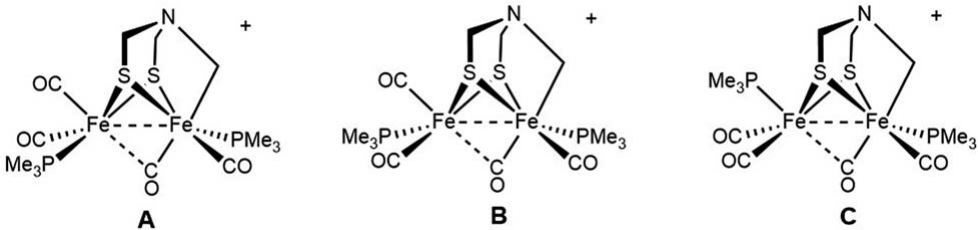


**Scheme 2:**
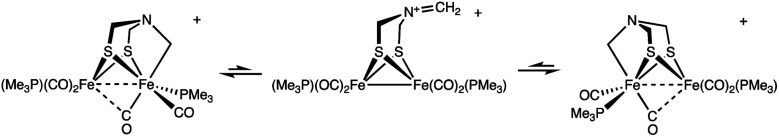
Proposed stereodynamics for [2]^+^. The process would proceed with racemization.

The entirety of the NMR data is accommodated by an exchange process involving reversible scission of the Fe–C bond, concomitant with regeneration of an Fe(i)Fe(i) species. Scission of the CH_2_–Fe bond introduces an effective plane of symmetry such that the two Fe(i) centers become equivalent (Scheme 2). Further relevant to stereodynamics, the exchange for the FeL_3_ sites is rapid in Fe(i)Fe(i) complexes, whereas bioctahedral Fe(ii)Fe(ii) complexes are more rigid.^24^

**Fig. 3:**
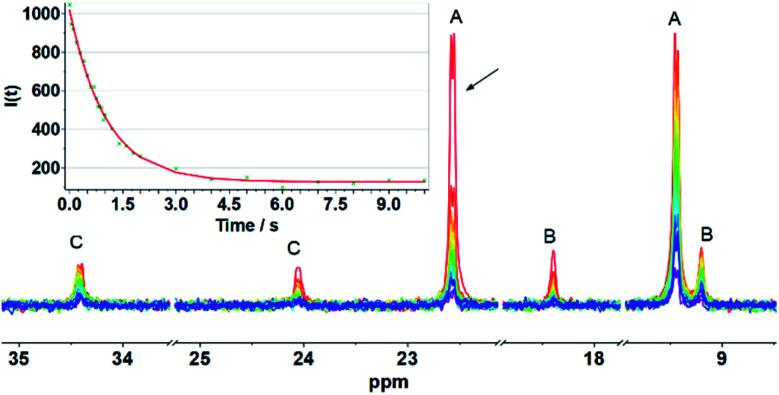
^31^P NMR spin saturation transfer spectra of [2]BAr^F^_4_ at 298 K in CD_2_Cl_2_. Irradiation of the signal at *δ* 22.59. *T*_1_ = 8.2 s for the resonance at *δ* 9.45. Inset: graph of intensity (*I*) of the *δ* 9.45 peak *vs.* irradiation time at *δ* 22.59. Fitting: *I*_*t*_ = *I*_0_ × {1/(1 + *τ*/8.2) × exp[−*t* × (1/8.2 + 1/*τ*)] + 1/(1 + 8.2/*τ*)}, where *τ* = 1.178 s, *k* = 1/*τ* = 0.85 s^−1^.

Evidence of the process shown in Scheme 2 is provided by ^31^P NMR spin saturation experiments. The *T*_1_ of the signal at *δ* 9.45 was determined to be 8.2 s, and the exchange rate was *k* = 0.85 s^−1^. Saturation of either of the signals at *δ* 22.45 or 9.45 resulted in collapse of the other five ^31^P NMR signals (Fig. 3).

### Mechanistic studies

Since Brønsted acids are required for the conversion of 1 to [2]^+^, we examined the ammonium complex [Fe_2_[(μ-SCH_2_)_2_NH_2_](CO)_4_(PMe_3_)_2_]^+^ ([1H]^+^). These results, which overlap with those reported earlier by Pickett,^25^ are rather fundamental and merit thorough analysis. ^1^H and ^31^P NMR spectra confirm that [1H]^+^ is stable in solution for days. Treating a CH_2_Cl_2_ solution of [1H]BF_4_ with NaBAr^F^_4_ induced tautomerization to the hydride [HFe_2_[(μ-SCH_2_)_2_NH](CO)_4_(PMe_3_)_2_]^+^ ([H1]^+^). According to IR measurements, the tautomerization is complete after 3 h at room temperature (eqn (2)).2
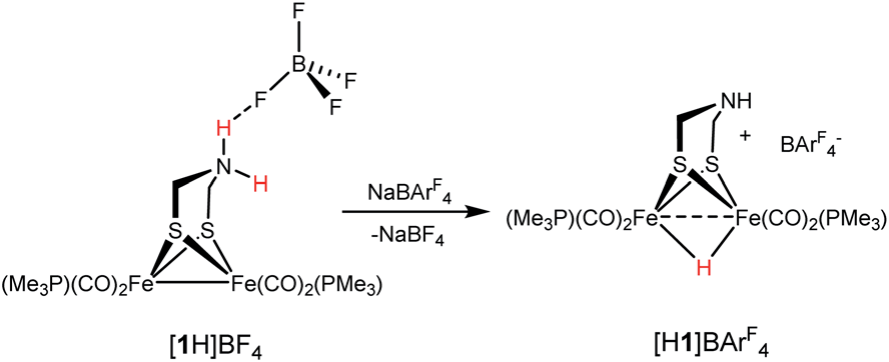


The ^1^H NMR spectrum for [H1]^+^ matches published data for related salts.^18^ The anion-dependent tautomerization reflects the stabilization of ammonium centers by hydrogen-bonding to BF_4_^−^, which persists in solution.^26^ One consequence of the ion-pairing (or its absence in the case of BAr^F^_4_^−^) is that the proton-induced reaction of 1 with CH_2_O is sensitive to the identity of the acid: H(OEt_2_)BF_4_ cleanly gives [2]^+^ but H(OEt_2_)_2_BAr^F^_4_, depending on the specific conditions, can afford significant quantities of the hydride [H1]^+^.

**Fig. 4:**
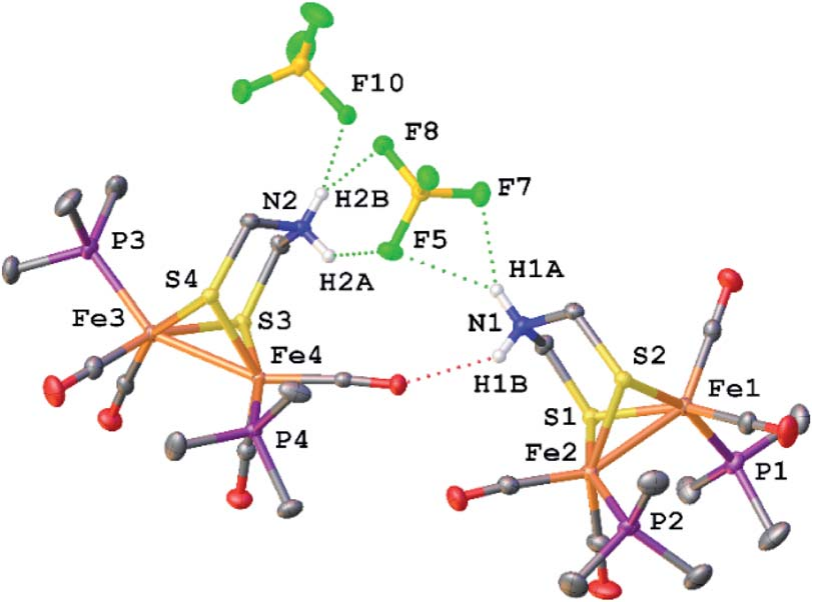
Structure of [Fe_2_[(μ-SCH_2_)_2_NH_2_](CO)_4_(PMe_3_)_2_]BF_4_ with thermal ellipsoids shown at 50% probability. H atoms except for the NH_2_ centres have been omitted for clarity. Two of the three ion pairs in the asymmetric unit are shown. Notice the presence of stereoisomers of [1H]^+^. Selected distances (Å): Fe1–Fe2, 2.5689(3); Fe3–Fe4 2.5444(3); H1A–F5, 2.54(2); H1A–F7, 2.10(2); H2A–F5, 2.30(2); H2B–F8, 2.41(2); H2B–F10, 2.00(2).

X-ray crystallography verified the extensive hydrogen-bonding in solid [1H]^+^ (Fig. 4). The asymmetric unit consists of three ion pairs; two cations have *trans*-dibasal phosphine ligands, one is apical–basal. All NH centers are hydrogen bonded to BF_4_^−^. The F⋯N distances range from 1.97–2.54 Å with the average distance of 2.22 Å.^27^

The hydroxymethylation of secondary amines by formaldehyde is well studied.^28^ When a solution of 1 was treated with CH_2_O in the absence of acid, only subtle shifts (<5 cm^−1^) were observed in the IR spectrum in the *ν*_CO_ region. It is known, however, that ν_C_O is relatively insensitive to substituents on nitrogen of the amine. For example, in this work we found that the *ν*_CO_ bands for Fe_2_[(μ-SCH_2_)_2_NR](CO)_4_(PMe_3_)_2_ are almost identical for R = H (1983, 1943, 1899 cm^−1^) and R = Me (1983, 1945, 1909, 1894 cm^−1^). ^1^H NMR spectroscopy proved to be a more sensitive indicator of the interaction of 1 and CH_2_O. A 1 : 0.25 mixture of these reactants generates ∼25% of a new species that we assign to the hydroxymethyl derivative Fe_2_[(μ-SCH_2_)_2_NCH_2_OH](CO)_4_(PMe_3_)_2_. The same species is observed with ^13^CH_2_O under otherwise identical conditions. In that experiment, the SCH_2_N groups did not show any enrichment. When 1 and CH_2_O were mixed in a 1 : 1 ratio, several species are observed, Fe_2_[(μ-SCH_2_)_2_NCH_2_OH](CO)_4_(PMe_3_)_2_, some unreacted 1, and what appears to be Fe_2_[(μ-SCH_2_)_2_N(CH_2_O)_*n*_CH_2_OH](CO)_4_(PMe_3_)_2_. Equilibration of these species is rapid, since the mixture reacts with 1 equiv. of HBF_4_ to cleanly give [2]^+^. No reaction was evident when 1 was treated with PhCHO, in the presence or absence of HBF_4_.

**Scheme 3:**
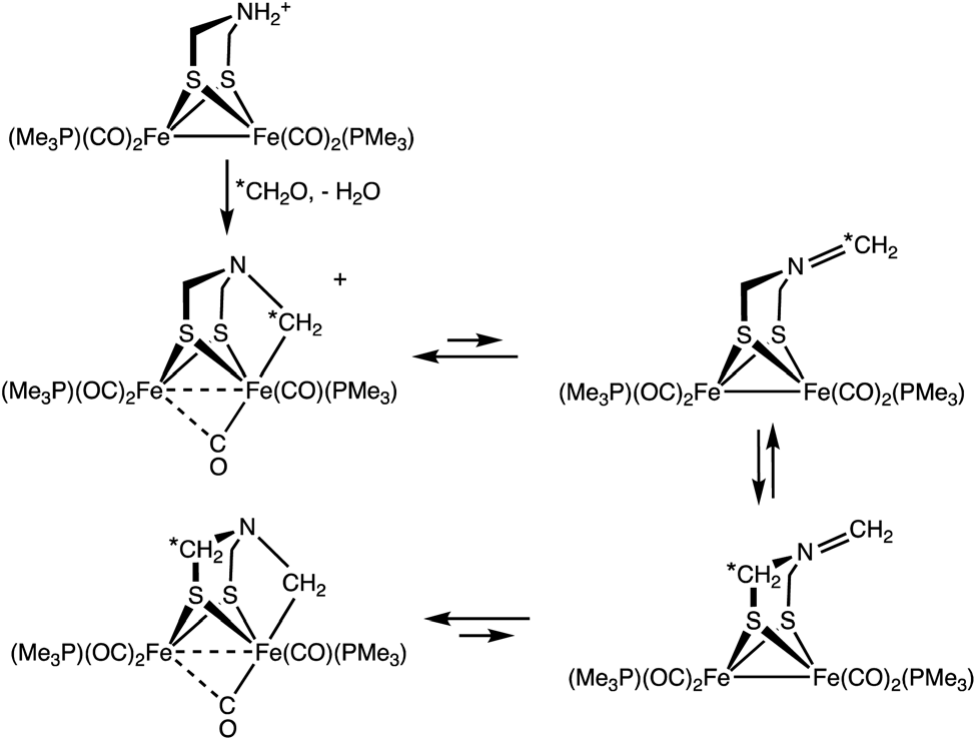
Synthesis of [2]^+^ using ^13^CH_2_O, showing site exchange.

Treatment of a solution of ^13^CH_2_O and 1 with H(Et_2_O)_2_BAr^F^_4_ gave [^13^2]^+^ with selective formation of the Fe–^13^CH_2_ isotopomer. Interestingly, this label exchanges with the other methylene groups in the complex over the course of hours (Scheme 3). The kinetics of exchange are first order in [2]^+^ up to about 90% conversion, which points to an intramolecular process. The NMR data show that this ^13^CH_2_/^12^CH_2_ exchange affects the diastereotopic SCH_2_ groups equally. We suggest that exchange occurs for the Fe(i)Fe(i) species where the diastereomerization is rapid. The ESI-MS of the product of the labelling shows only singly labelled [2]^+^. Intermolecular processes would be expected to yield detectable levels of doubly labeled product.

### Reaction of [Fe_2_[(μ-SCH_2_)_2_NH](CN)_2_(CO)_4_]^2−^ with CH_2_O/HBF_4_

**Scheme 4:**
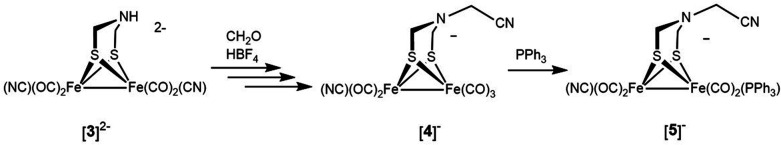
Pathway for formation of [Fe_2_[(μ-SCH_2_)_2_NCH_2_CN](CN)(CO)_4_(PPh_3_)]^−^ ([5]^−^).

The reaction of paraformaldehyde with [Fe_2_[(μ-SCH_2_)_2_NH](CN)_2_(CO)_4_]^2−^ ([3]^2−^) was investigated because this complex resembles the [2Fe]_H_ active site, which is also a dicyanide. In the presence of one equiv. of HBF_4_, [3]^2−^ converts to the ammonium derivative, which is stable in MeCN solution for several minutes prior to irreversible tautomerization to the hydride (Fig. S39†). Addition of paraformaldehyde to [3H]^−^ gives one main product, which we assign as the pentacarbonyl [Fe_2_[(μ-SCH_2_)_2_NCH_2_CN](CN)(CO)_5_]^−^ ([4]^−^). This formula is supported by ESI-MS analysis. Attempted purification of [4]^−^ was unsuccessful, however its FT-IR spectrum is very similar to that for [Fe_2_[(μ-S_2_C_3_H_6_)](CN)(CO)_5_]^−^. When ^13^CH_2_O was used, the singly labeled product was generated according to ESI-MS. We propose that [4]^−^ arises by reductive elimination of the nitrile from [Fe_2_[(μ-SCH_2_)_2_NCH_2_](CN)_2_(CO)_4_]^−^ followed by CO-scavenging (Scheme 4).

**Fig. 5:**
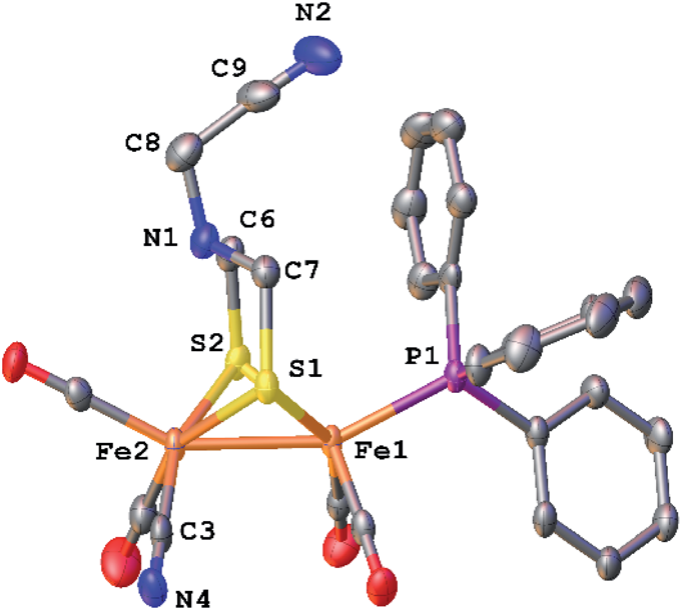
Structure of Et_4_N[Fe_2_[(μ-SCH_2_)_2_NCH_2_CN](CN)(CO)_4_(PPh_3_)] (Et_4_N[5]) with thermal ellipsoids shown at 50% probability. H atoms and the Et_4_N^+^ cation have been omitted for clarity. Selected distances (Å): Fe1–Fe2, 2.5263(8); Fe2–C3, 1.928(5); C3–N4, 1.148(6); C9–N2, 1.144(8).

The phosphine derivative of [4]^−^ was obtained when the reaction of [3]^2−^ with CH_2_O was conducted in the presence of PPh_3_ (Scheme 4). The ^31^P NMR signal of this product at *δ* 60.1 indicates coordination of PPh_3_, leading to the formula [Fe_2_[(μ-SCH_2_)_2_NCH_2_CN](CN)(CO)_4_(PPh_3_)]^−^ ([5]^−^). In the FT-IR spectrum of [5]^−^, the *ν*_CO_ bands are shifted by 21 cm^−1^ toward high energy compared to dicyanide complex [3]^2−^. The structure of [5]^−^ was verified by X-ray crystallography (Fig. 5), which confirms the presence of a conventional [Fe_2_(μ-SR)_2_(CN)(CO)_4_(PPh_3_)]^−^ complex,^29^ and, most importantly, the presence of the cyanomethyl substituent.

### Reactions of methylenated FeFe complex with nucleophiles

The Fe–CH_2_N bond in [2]^+^ is electrophilic. For example, treating [2]^+^ with PPh_3_ cleanly gave [Fe_2_[(μ-SCH_2_)_2_NCH_2_PPh_3_](CO)_4_(PMe_3_)_2_]BF_4_ ([6]^+^), the result of C–P bond formation. Dealkylation of Fe involves reduction to an [Fe(i)]_2_ complex (eqn (3)).3
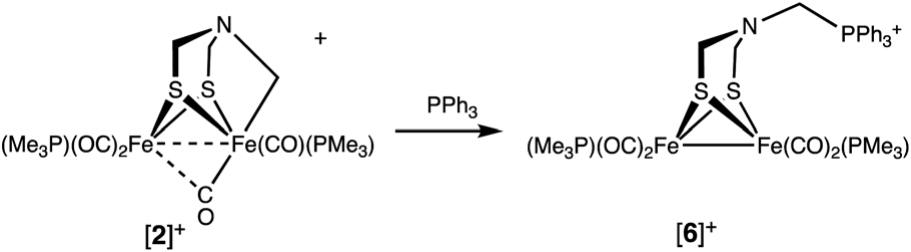


The PMe_3_ ligands in [6]^+^ appear equivalent, as is typical for related [Fe(i)]_2_ complexes. The presence of a phosphonium center is indicated by the ^31^P NMR singlet at *δ* 9.17, much higher field than *δ* 65.7 for Fe_2_(μ-S_2_C_3_H_6_)(CO)_5_(PPh_3_).^30^ In the region assigned to NCH_2_P, the ^1^H NMR spectrum features a broad signal at *δ* 4.90. The broadness is associated with the nonequivalent protons, each of which is coupled to ^31^P. The IR spectrum of [6]BF_4_ also agrees with reduction of Fe(ii)Fe(ii) to Fe(i)Fe(i): *ν*_CO_ shifts to lower frequency by 55 cm^−1^ (1978, 1948, 1903 cm^−1^). These frequencies are comparable to those in 1.

**Fig. 6:**
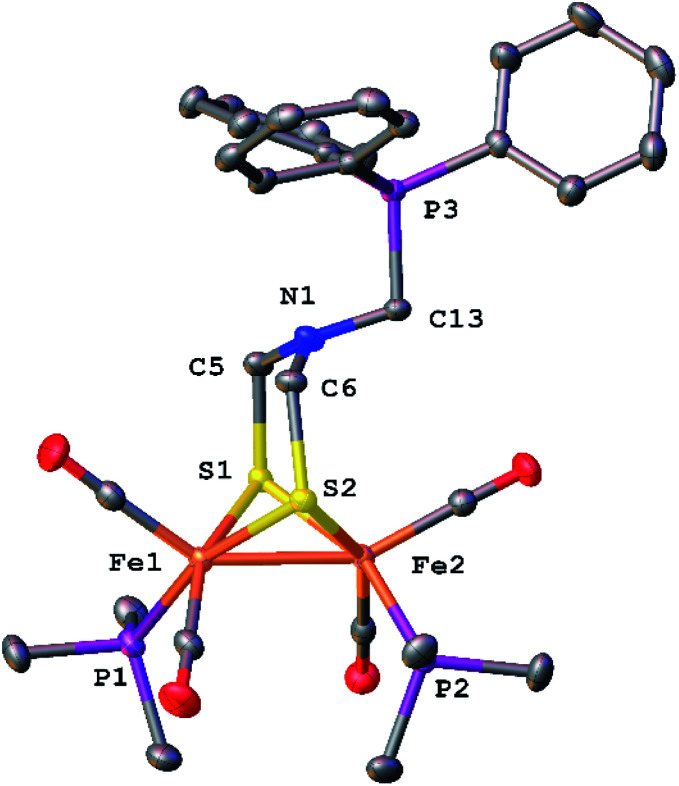
Structure of [Fe_2_[(μ-SCH_2_)_2_NCH_2_PPh_3_](CO)_4_(PMe_3_)_2_]BF_4_ ([6]BF_4_) with thermal ellipsoids shown at 50% probability. H atoms and BF_4_^−^ anion have been omitted for clarity. Selected distances (Å): Fe1–Fe2, 2.5706(4); N1–C5, 1.436(3); N1–C6, 1.445(3); N1–C13, 1.449(3).

The structure of [6]BF_4_ was verified by X-ray crystallography (Fig. 6). The complex is a conventional Fe_2_(μ-SR)_2_(CO)_6−*x*_L_*x*_ butterfly. The bulky phosphonium substituent is distant from the Fe_2_ core.

Conversion of [2]^+^ back to 1 was induced upon treatment with Et_4_NOH. From this reaction, 1 was recovered in 40% yield after purification by column chromatography. The electrophilic nature of the Fe–CH_2_ bond is also supported by the reaction of [2]^+^ with BH(OAc)_3_^−^, a mild hydride donor. In this case, Fe_2_[(μ-SCH_2_)_2_NMe](CO)_4_(PMe_3_)_2_ was obtained in good yield (eqn (4)).4
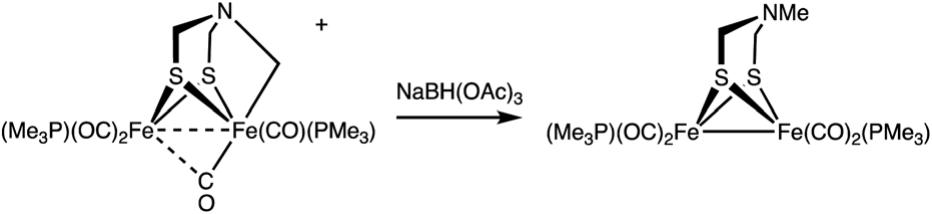


## Conclusions

**Scheme 5:**
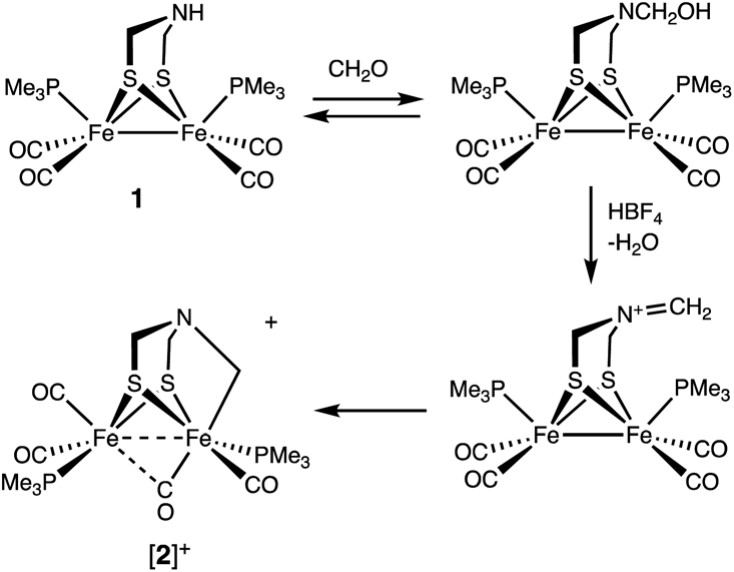
Proposed pathway for the methylenation of 1.

As a specific conclusion, this work provides a plausible model for the inhibition of [FeFe]-hydrogenases by formaldehyde. The methylene group donated by formaldehyde occupies both substrate binding sites, amine and the distal Fe. The methylenation proceeds by addition of the aldehyde to the secondary amine followed by generation of the iminium cation, which oxidatively adds to one of the Fe(i) centers, oxidizing the diiron site by 2e^−^ (Scheme 5). Many examples exist for the addition of iminium cations to low-valent metals.^31^ The methylenation reaction does not proceed from the diiron μ-hydride. In accord with the results of Bachmeier *et al.*,^13^ the methylenation is selective for reduced state(s) of the diiron center, as required for oxidative addition.

Complex [2]^+^ represents a rare mimic of a terminal hydride for the [FeFe]-hydrogenases. Normally terminal hydrides of synthetic diiron dithiolates rapidly isomerize,^20^ which precludes extensive characterization of this key intermediate.^32^ In the Mulheim mechanism, the diferrous terminal hydride corresponds to H_hyd_ state, defined as [4Fe–4S]^+^–Fe_p_(ii)–amine–Fe_d_(ii)H. In this state, the hydride is protic, being reversibly deprotonated by the amine cofactor. Consistent with this model, the alkyl ligand in [2]^+^ is electrophilic. Furthermore, analogous to the reversible deprotonation of H_hyd_, [2]^+^ reversibly dealkylates (see Scheme 2) to give a Fe(i)Fe(i) species as proposed for the H_sred_H^+^ state. Complex [2]^+^ exists as an equilibrium mixture of three isomers. The finding that these isomers are separated by less than ∼1 kcal mol^−1^, shows that stereochemistry of the other ligands on the diiron dithiolate has little influence on the Fe–alkyl bond (and by inference Fe–hydride bond). Also like H_hyd_ state, [2]^+^ has a highly unsymmetrical semi-bridging CO *trans* to R (= alkyl, hydride) on the distal Fe is persistent. In a future paper we plan to describe the redox chemistry of this electrophilic Fe(ii)Fe(ii) in our quest to further probe analogues of the elusive H_hyd_H^+^ state.

**Scheme 6:**
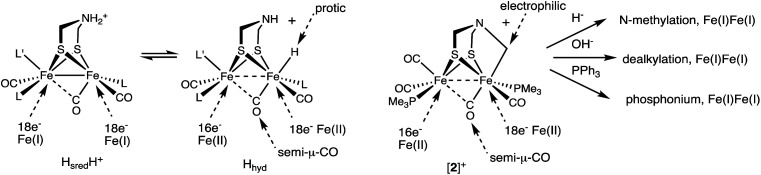
Comparison of the [2Fe]_H_ centers in the H_sred_H^+^ and H_hyd_ states (L = CN^−^) and [2]^+^, including its reactions.

As established by its reactions with a range of nucleophiles (Scheme 6), [2]^+^ presents opportunities for appending the Fe_2_(μ-SR)_2_ center to other scaffolds.

The susceptibility of [2]^+^ to nucleophilic attack is reminiscent of Co(iii)-alkyls as represented by vitamin B_12_ and its derivatives and models.^33,34^ Given the vast chemistry of B_12_ mimics, it is possible that a wide range of diiron alkyl chemistry awaits discovery and development.

## Experimental

### Materials and methods

Reactions were conducted in stirred solutions or slurries under nitrogen at room temperature unless otherwise indicated. Sample work-up routinely included rinsing solids with Et_2_O or pentane and storage under vacuum to remove traces of solvent. All reactions and purifications were conducted using standard Schlenk techniques or in an MBraun glovebox under N_2_. Solvents were purified using solvent purification system equipped with alumina filtration column. CD_2_Cl_2_ was degassed by freeze–pump–thaw cycles and dried using 4 Å molecular sieves. ^1^H, ^31^P{^1^H}, and ^13^C NMR spectra were recorded on Varian 500, Varian 600, Bruker 500, or Bruker Ascend 600 MHz spectrometers. Chemical shifts (*δ*/ppm) are referenced to residual solvent peak (5.32 ppm for ^1^H and 53.84 ppm for ^13^C in CD_2_Cl_2_). Chemical shifts (*δ*/ppm) for ^31^P{^1^H} NMR were calibrated using 85% H_3_PO_4_ as an external reference (0 ppm). Solution IR spectra were recorded on a PerkinElmer Spectrum 100 FTIR spectrometer. Elemental analysis was performed utilizing an Exeter CE-440 elemental Analyzer. A Waters Micromass Quattro II spectrometer was used to acquire ESI-MS data. Crystallographic data were collected on a Bruker D8 Venture kappa diffractometer equipped with a Photon II CPAD detector. An Iμs Microfocus Mo source (*λ* = 0.71073 Å) coupled with a multi-layer mirror monochromator provided the incident beam. Literature procedures were followed for the synthesis of (Et_4_N)_2_[Fe_2_[(μ-SCH_2_)_2_NH](CN)_2_(CO)_4_],^35^ Fe_2_[(μ-SCH_2_)_2_NH](CO)_4_(PMe_3_)_2_,^25^ and Fe_2_[(μ-SCH_2_)_2_NMe](CO)_4_(PMe_3_)_2_.^23,25^ Other chemicals were purchased from commercial sources and used without further purification.

#### [Fe_2_[(μ-SCH_2_)_2_NH_2_](CO)_4_(PMe_3_)_2_]BF_4_ ([1H]BF_4_)

To a solution of Fe_2_[(μ-SCH_2_)_2_NH](CO)_4_(PMe_3_)_2_ (1) (100 mg, 0.21 mmol) in 10 mL of CH_2_Cl_2_ was added HBF_4_·Et_2_O (34 mg, 0.21 mmol). After 10 min, the solution was concentrated to ∼1 mL, and a red solid was precipitated upon addition of 10 mL of Et_2_O. Yield: 95 mg (81%). ^1^H NMR (500 MHz, CD_2_Cl_2_): *δ* 6.36 (s, 2H, NH_2_), 3.49 (s, 4H, (SCH_2_)_2_N), 1.57 (d, 18H, P(CH_3_)_3_). ^31^P{^1^H} NMR (202 MHz, CD_2_Cl_2_): *δ* 26.24. IR (CH_2_Cl_2_): *ν*_CO_ = 2000, 1963, 1923 cm^−1^. Anal. calcd for C_12_H_25_BF_4_Fe_2_NO_4_P_2_S_2_: C, 25.2; H, 4.41; N, 2.45. Found, C, 25.06; H, 4.05; N, 2.32.

#### [HFe_2_[(μ-SCH_2_)_2_NH](CO)_4_(PMe_3_)_2_]BAr^F^_4_ ([H1]BAr^F^_4_)

To a solution of [1H]BF_4_ (40 mg, 0.070 mmol) in 4 mL of CH_2_Cl_2_ was added solid NaBAr^F^_4_ (73 mg, 0.070 mmol). IR spectra showed full conversion to [H1]BAr^F^_4_ after 3 h. The reaction mixture was filtered through Celite and evaporated. The residue was recrystallized using CH_2_Cl_2_/pentane at −30 °C to give a red crystalline solid. Yield: 85% (79.9 mg). ^1^H NMR (500 MHz, CD_2_Cl_2_): *δ* 7.72 (s, 8H, ArH), 7.57 (s, 4H, ArH), 4.13 (s, 4H, SCH_2_), 2.32 (p, *J* = 8.8 Hz, 1H, NH), 1.55 (d, *J*_PH_ = 10.2 Hz, 18H, P(CH_3_)_3_), −14.52 (t, *J*_PH_ = 21.5 Hz, 1H). ^31^P{^1^H} NMR (202 MHz, CD_2_Cl_2_): *δ* 21.81. IR (CH_2_Cl_2_): *ν*_CO_ = 2033, 1992 cm^−1^. ESI-MS *m*/*z* calcd for [M^+^], 483.9. Found, 484.0. Anal. calcd for C_44_H_35_BF_24_Fe_2_NO_4_P_2_S_2_: C, 39.25; H, 2.62; N, 1.04. Found, 39.23; H, 2.57; N, 1.27.

#### [Fe_2_[(μ-SCH_2_)_2_NCH_2_](CO)_4_(PMe_3_)_2_]BF_4_ ([2]BF_4_)

A solution of Fe_2_[(μ-SCH_2_)_2_NH](CO)_4_(PMe_3_)_2_ (1) (200 mg, 0.41 mmol) in 20 mL of CH_2_Cl_2_ was treated with CH_2_O (25 mg, 0.83 mmol). After stirring this mixture for 1 h, HBF_4_·Et_2_O (67 mg, 0.41 mmol) was added by syringe. The color of the reaction mixture immediately changed from red to dark brown. After a further 30 min, the mixture was analyzed by IR spectroscopy, which showed that the bands for 1 were replaced by new bands at higher energy. The solution was concentrated to 5 mL under vacuum. Addition of 40 mL of Et_2_O precipitated the black solid. Yield: 207 mg (86%). ^1^H NMR (500 MHz, CD_2_Cl_2_): *δ* 5.51 (br, 1H, NCH_2_Fe), 5.14 (br, 1H, NCH_2_Fe), 3.95 (br, 2H, SCH_2_), 3.31 (br, 2H, SCH_2_), 1.69 (br, 18H, P(CH_3_)_3_). ^31^P{^1^H} NMR (203 MHz, CD_2_Cl_2_): *δ* 35.00 (br), 24.67, 24.28, 22.70 (br), 8.94 (br). IR (CH_2_Cl_2_): *ν*_CO_ = 2044, 2017, 1990, 1942 cm^−1^. HR-MS (ESI) *m*/*z* calcd for [M^+^], 495.9321. Found, 495.9312. Elemental analysis was obtained on the BAr^F^_4_^−^ salt (see next procedure).

#### [Fe_2_[(μ-SCH_2_)_2_NCH_2_](CO)_4_(PMe_3_)_2_]BAr^F^_4_ ([2]BAr^F^_4_)

A 20 mL vial was loaded with [2]BF_4_ (50 mg, 0.086 mmol), 1.0 equiv. of NaBAr^F^_4_ (88 mg, 0.086 mmol), followed by 5 mL of CH_2_Cl_2_. After stirring the mixture for 1 h, solvent was removed *in vacuo*. The residue was extracted into 2 mL of CH_2_Cl_2_. This extract was filtered through Celite and layered with hexane. Dark-brown microcrystals were obtained after 2 days. Yield: 110 mg (95%). ^1^H NMR (500 MHz, CD_2_Cl_2_): *δ* 7.73 (m, 8H, ArH), 7.57 (s, 4H, ArH), 5.52 (m, 1H, NCH_2_Fe), 4.79 (m, 1H, NCH_2_Fe), 4.17 (dd, *J* = 11.5, 3.8 Hz, 1H, SCH_2_), 3.84 (dd, *J* = 11.8, 8.7 Hz, 1H, SCH_2_), 3.25 (dd, *J* = 11.8, 3.4 Hz, 2H, SCH_2_). 1.66 (d, *J*_PH_ = 10.3 Hz, 9H, P(CH_3_)_3_), 1.53 (d, *J*_PH_ = 10.4 Hz, 9H, P(CH_3_)_3_). ^31^P{^1^H} NMR (243 MHz, CD_2_Cl_2_), three isomers were detected: *δ* 22.59 (d, *J*_PP_ = 7.3 Hz), 9.45 (d, *J*_PP_ = 7.4 Hz), *trans*-dibasal; 34.46 (d, *J*_PP_ = 7.7 Hz), 24.10 (d, *J*_PP_ = 7.6 Hz), *cis*-dibasal; 18.41 (s), 9.21 (s), apical-basal. ^13^C NMR (126 MHz, CD_2_Cl_2_): *δ* 212.37–210.12 (m, *t*-CO), 201.90 (d, *J*_PC_ = 19.7 Hz, μ-CO), 162.18 (q, ^1^*J*_BC_ = 49.8 Hz), 135.22, 131.08–128.70 (qq, ^2^*J*_CF_ = 31.4 Hz, ^4^*J*_CF_ = 2.9 Hz), 125.03 (q, ^1^*J*_CF_ = 272.4 Hz), 118.55–116.81 (m), 75.77 (d, ^2^*J*_PC_ = 12.0 Hz, NCH_2_Fe), 58.45 (s, SCH_2_), 58.15 (d, ^3^*J*_PC_ = 5.5 Hz, SCH_2_), 18.74 (d, ^1^*J*_PC_ = 33.4 Hz, P(CH_3_)_3_), 16.69 (d, ^1^*J*_PC_ = 32.0 Hz, P(CH_3_)_3_). IR (CH_2_Cl_2_): *ν*_CO_ = 2046, 2020, 1992, 1942 (μ-CO). Anal. calcd for C_45_H_36_BF_24_Fe_2_NO_4_P_2_S_2_: C, 39.76; H, 2.67; N, 1.03. Found, C, 39.33; H, 2.59; N, 1.09. Single crystals were grown by diffusion of hexane into a CH_2_Cl_2_ solution.

#### Reaction of [2]BF_4_ with NaBH(OAc)_3_

To a solution of [2]BF_4_ (15 mg, 0.026 mmol) in 2 mL of MeCN was added NaBH(OAc)_3_ (5.5 mg, 0.026 mmol, 1 equiv.). The reaction solution changed from dark brown to red immediately. After a further 10 min, solvent was removed, and the residue was extracted into pentane. Removing the solvent under vacuum gave Fe_2_[(SCH_2_)_2_NMe](CO)_4_(PMe_3_)_2_ as a red solid. Yield: 86% (11 mg). The NMR spectrum of this product matches that of authentic Fe_2_[(μ-SCH_2_)_2_NMe](CO)_4_(PMe_3_)_2_.^25^^1^H NMR (500 MHz, CD_2_Cl_2_): *δ* 2.91 (s, 4H, SCH_2_), 2.10 (s, 3H, NCH_3_), 1.49 (d, *J*_PH_ = 9.1 Hz, 18H, P(CH_3_)_3_). ^31^P{^1^H} NMR (202 MHz, CD_2_Cl_2_): *δ* 22.81. ESI-MS *m*/*z* calcd for [M + H^+^], 497.9. Found, 498.2. IR (CH_2_Cl_2_): *ν*_CO_ = 1983, 1945, 1909, 1894 sh cm^−1^.

#### [Fe_2_[(μ-SCH_2_)_2_NCH_2_PPh_3_](CO)_4_(PMe_3_)_2_]BF_4_ ([6]BF_4_)

A solution of PPh_3_ (23 mg, 0.086 mmol) in 2 mL of CH_2_Cl_2_ was added dropwise to a solution of [2]BF_4_ (50 mg, 0.086 mmol) in 2 mL of CH_2_Cl_2_. The color changed from purple to red immediately. After stirring for 10 min, the solution was concentrated to 1 mL. The concentrate was layered with 10 mL of Et_2_O, and this biphasic mixture was stored at −30 °C. Red crystals appeared after 24 h. Yield: 66 mg (91%). ^1^H NMR (500 MHz, CD_2_Cl_2_): *δ* 8.49–7.29 (m, 5H, ArH), 4.90 (s, 2H, NCH_2_P), 3.45 (s, 4H, SCH_2_), 1.50 (d, *J*_PH_ = 9.2 Hz, 18H, P(CH_3_)_3_). ^31^P{^1^H} NMR (203 MHz, CD_2_Cl_2_): *δ* 24.15, 9.18. IR (CH_2_Cl_2_): *ν*_CO_ = 1978, 1948, 1903. ESI-MS: *m*/*z* calcd for [M^+^ − CO], 730.4. Found, 730.0. Anal. calcd for C_31_H_39_BF_4_Fe_2_NO_4_P_3_S_2_: C, 44.05; H, 4.65; N, 1.66. Found, C, 43.63; H, 4.96; N, 1.80. Single crystals were grown by diffusion of Et_2_O into a CH_2_Cl_2_ solution at −30 °C.

#### Reaction of [Fe_2_[(μ-SCH_2_)_2_NCH_2_](CO)_4_(PMe_3_)_2_]BF_4_ ([2]BF_4_) with Et_4_NOH

To a solution of [2]BF_4_ (40 mg, 0.069 mmol) in 2 mL of THF was added Et_4_NOH (10.1 mg, 0.069 mmol, 1.0 equiv., 20% wt aqueous solution). After 2 h, the reaction mixture was evaporated to dryness. The residue was extracted into 1 mL of CH_2_Cl_2_. This extract was filtered through Celite to remove Et_4_NBF_4_. A concentrate of this filtrate was purified by column chromatography on silica gel eluting with Et_2_O/pentane. Yield of 1: 13 mg (40%). Product as 1 was identified by FT-IR and ^1^H NMR spectroscopy, as well as TLC.

#### Et_4_N[Fe_2_[(μ-SCH_2_)_2_NCH_2_CN](CN)(CO)_4_(L)] ((Et_4_N[4] (L = CO) and Et_4_N[5] (L = PPh_3_)

##### Et_4_N[4]

A solution of (Et_4_N)_2_[Fe_2_[(μ-SCH_2_)_2_NH](CN)_2_(CO)_4_] (50 mg, 0.078 mmol) in MeCN was treated with paraformaldehyde (4.7 mg, 0.016 mmol). After stirring this mixture for 2 h, a solution of H(OEt_2_)BF_4_ (13 mg, 0.078 mmol) in 2 mL of MeCN was added dropwise. The color of the reaction mixture changed from deep red to dark brown immediately. FT-IR: *ν*_CO_ = 2038, 2000, 1980, 1945, 1935 (sh), 1912 cm^−1^; *ν*_CN_ = 2108 cm^−1^. ESI-MS: *m*/*z* calcd for [M^−^], 423.8. Found, 423.8. When the experiment was conducted in the presence of ^13^CH_2_O, the FT-IR spectrum was the same. ESI-MS: *m*/*z* calcd for [M^−^], 424.8. Found, 424.8.

##### Et_4_N[5]

The experiment above was repeated using [HPPh_3_]BF_4_ (27 mg, 0.078 mmol) in place of H(OEt_2_)BF_4_. A solution of (Et_4_N)_2_[Fe_2_[(μ-SCH_2_)_2_NH](CN)_2_(CO)_4_] (50 mg, 0.078 mmol) in MeCN was treated with paraformaldehyde (4.7 mg, 0.016 mmol). After 2 h, the IR spectrum showed no change in the CO region. A solution of [HPPh_3_]BF_4_ (27 mg, 0.078 mmol) in 2 mL of MeCN was then added dropwise. The color of the reaction mixture changed from red to dark brown immediately. After 12 h, the color turned to red again. The mixture was then concentrated to 2 mL, and the concentrate was filtered through Celite and layered with 20 mL of Et_2_O. After 2 days at −30 °C, the layered solution yielded a red solid. Yield: 75% (45 mg). ^1^H NMR (600 MHz, CD_2_Cl_2_): *δ* 7.68–7.66 (m, 6H, ArH) 7.40–7.39 (m, 9H, ArH), 3.17–3.15 (q, 8H, ^+^N(CH_2_CH_3_)_4_), 2.57–2.52 (br, 4H, SCH_2_), 2.43 (s, 2H, NCH_2_CN), 1.25 (t, 12H, ^+^N(CH_2_CH_3_)_4_). ^31^P{^1^H} NMR (203 MHz, CD_2_Cl_2_): *δ* 60.06. ^13^C NMR (151 MHz, CD_2_Cl_2_): *δ* 218.28 (CO), 138.69 (d, CN), 133.71 (d, *J*_PC_ = 11.5 Hz, P(C_6_H_5_)_3_), 129.67 (d, *J*_PC_ = 2 Hz, P(C_6_H_5_)_3_), 128.44 (d, *J*_PC_ = 9.0 Hz, P(C_6_H_5_)_3_), 114.82 (NCH_2_CN), 53.23 (^+^N(CH_2_CH_3_)_4_), 50.64 (SCH_2_), 46.77 (NCH_2_CN), 8.00 (^+^N(CH_2_CH_3_)_4_). IR (CH_2_Cl_2_): *ν*_CO_ = 1988, 1950, 1916 cm^−1^, *ν*_CN_ = 2081 cm^−1^. Anal. calcd for C_35_H_41_Fe_2_N_4_O_4_PS_2_·0.2CH_2_Cl_2_: C, 52.49; H, 5.18; N, 6.96. Found, C, 52.48; H, 5.42; N, 7.15. ESI-MS: *m*/*z* calcd for [M^−^], 657.9. Found, 657.9. Single crystals were grown by diffusion of Et_2_O into a CH_2_Cl_2_ solution at room temperature.

## Data availability

All experimental and crystallographic data are available in the ESI.†

## Author contributions

Methodology, investigation, writing: F. Z.; conceptualisation and writing: T. B. R. Investigation: L. Z. and T. J. W. All authors have given approval to the final version of the manuscript.

## Conflicts of interest

There are no conflicts to declare.

## Supplementary Material

SC-012-D1SC05803G-s001

SC-012-D1SC05803G-s002
