# Supplementary material for: Inhibition of [FeFe]-hydrogenase by formaldehyde: proposed mechanism and reactivity of FeFe alkyl complexes
Source: Chem Sci. 2021 Nov 16;12(47):15673–81. doi: 10.1039/d1sc05803g (PMC8653999; doi:10.1039/d1sc05803g)
Supplement: SC-012-D1SC05803G-s001 [file SC-012-D1SC05803G-s001.pdf]

Electronic Supplementary Material (ESI) for Chemical Science.
This journal is © The Royal Society of Chemistry 2021

Supporting Information

Inhibition of [FeFe]-Hydrogenase by Formaldehyde: Proposed Mechanism and Reactivity of FeFe Alkyl Complexes

Fanjun Zhang,* Toby J. Woods, Lingyang Zhu and Thomas B. Rauchfuss*
School of Chemical Sciences
University of Illinois
Urbana, IL 61801, USA

Table of Contents

Spectra for $[Fe_2[(SCH_2)_2NCH_2](CO)_4(PMe_3)_2]BF_4$ ([2]BF_4) and ([2]BAr^F_4)............S3

Exchange Process in [2]BAr^F_4 Determined by Spin Saturation Transfer S15

Mechanistic Studies for Formation of $[2]^+$ S20

1. Spectra for $[Fe_2[(SCH_2)_2NH_2](CO)_4(PMe_3)_2]BF_4$ ([**1**H]BF_4)................................S20
2. Spectra for $[HFe_2[(SCH_2)_2NH](CO)_4(PMe_3)_2]BAr^F_4$ ([H**1**]BAr^F_4)..........................S23
3. Reaction of $[Fe_2[(SCH_2)_2NH](CO)_4(PMe_3)_2]$ (**1**) or [H**1**]BAr^F_4 with CH_2O...........S26
4. IR for the reaction of **1** + PhCHO + HBF_4 ....S31
5. Detection of the *C*H_2 exchange in [**2**]BAr^F_4 by ^{13}C NMR S32

Spectra for $Et_4N[Fe_2[(SCH_2)_2NCH_2CN](CN)(CO)_4PPh_3]$ (Et_4N[5])........................S34

Mechanistic Studies for Formation of Et_4N[5] S42

Spectra for $[Fe_2[(SCH_2)_2NCH_2PPh_3](CO)_4(PMe_3)_2]^+$ ($[6]^+$) S45

Spectra for $Fe_2[(SCH_2)_2NMe](CO)_4(PMe_3)_2$..S51

Crystal Data and Structure Refinements..S55

Crystallography...S57

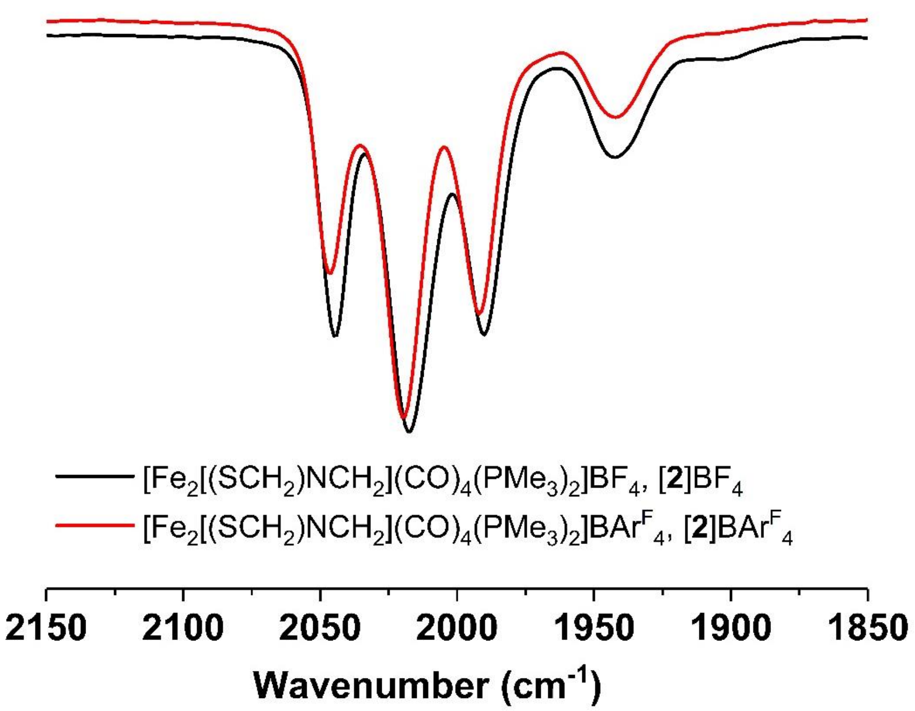

Figure S1. IR spectra of $[Fe_2(SCH_2)_2NCH_2(CO)_4(PMe_3)_2]BF_4$ ([**2**]BF_4) and $[Fe_2(SCH_2)_2NCH_2(CO)_4(PMe_3)_2]BAr^F{}_4$ ([**2**]$BAr^F{}_4$) in CH_2Cl_2 solution.

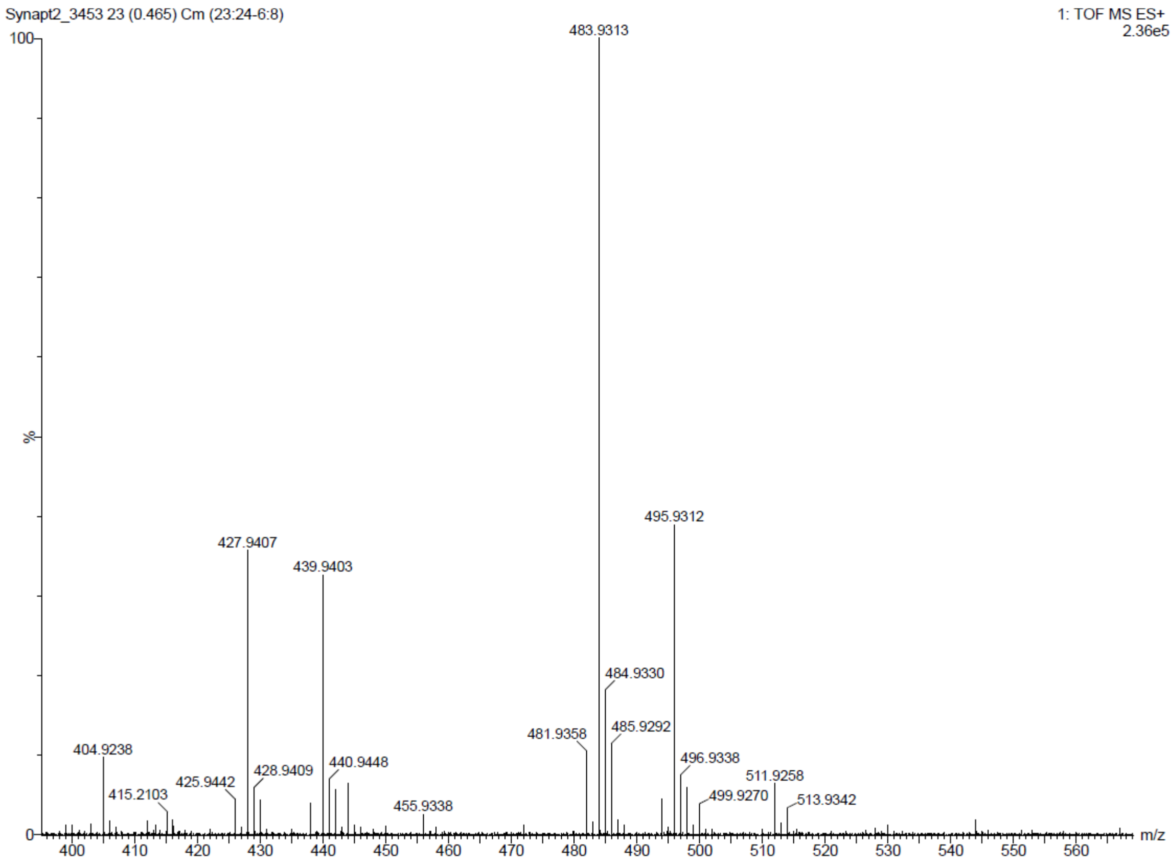

Figure S2. HR-MS (ESI) spectrum of $[Fe_2[(SCH_2)_2NCH_2](CO)_4(PMe_3)_2]BF_4$ ([**2**]BF_4) in CH_2Cl_2 solution.

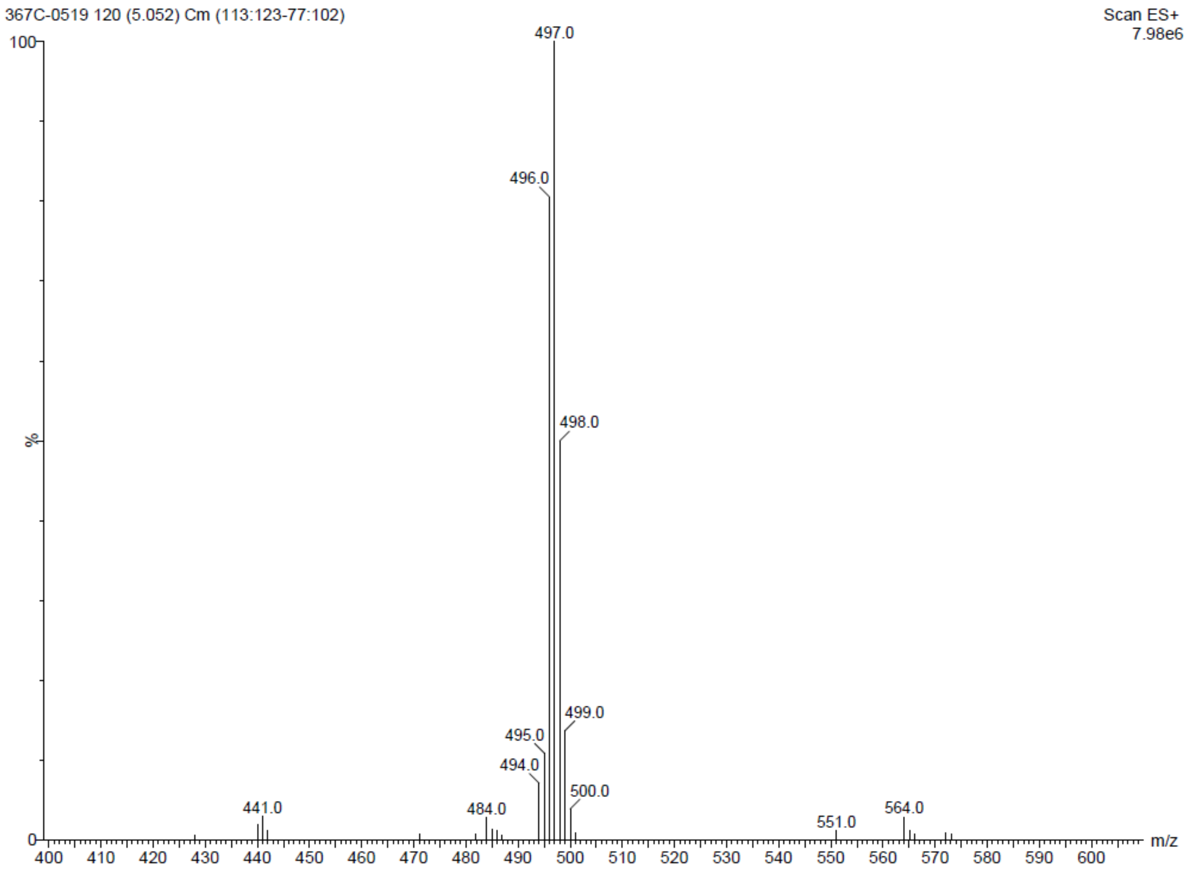

Figure S3. ESI-MS spectrum of ^{13}C labeled $[Fe_2[(SCH_2)_2NCH_2](CO)_4(PMe_3)_2]BF_4$ ([**2**]BF_4) in CH_2Cl_2 solution.

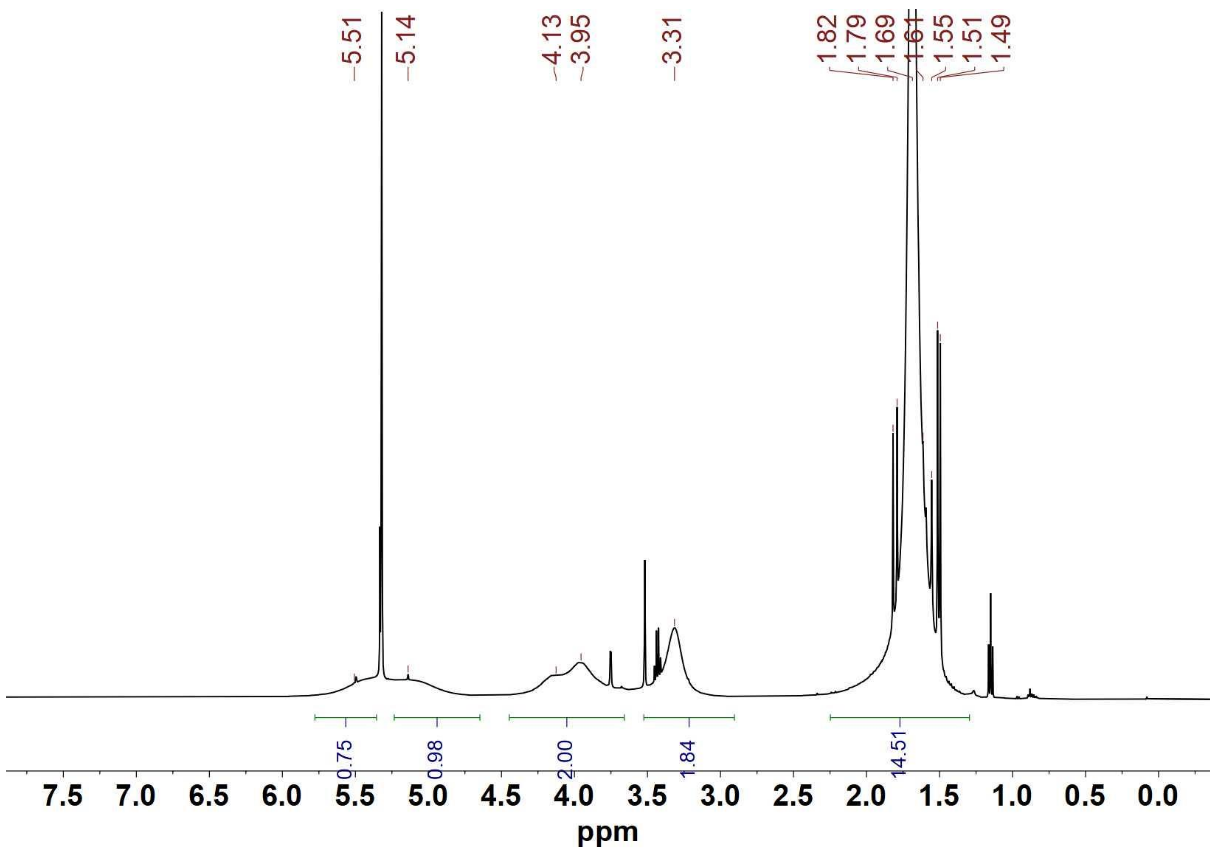

Figure S4. ^{1}H NMR spectrum of $[Fe_2[(SCH_2)_2NCH_2](CO)_4(PMe_3)_2]BF_4$ ([**2**]BF_4) in CD_2Cl_2 at room temperature.

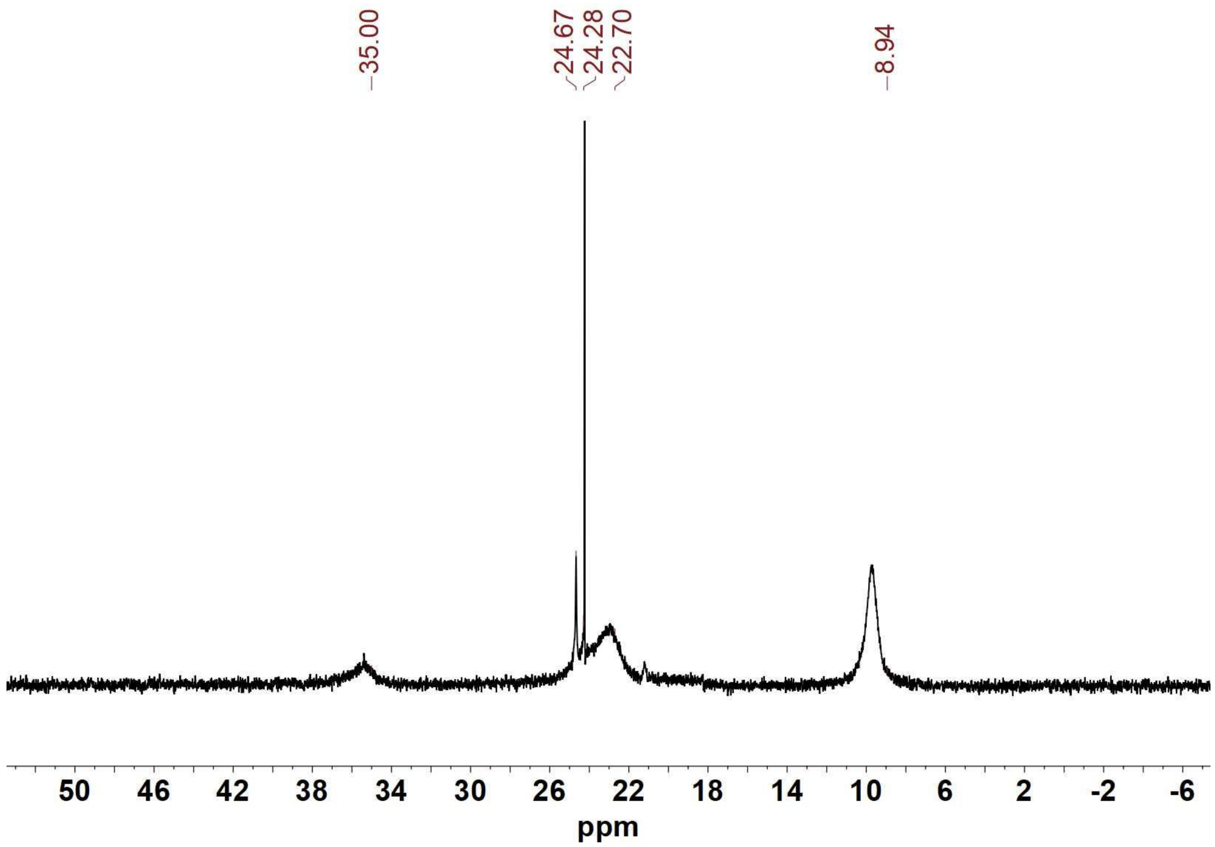

Figure S5. ^{31}P NMR spectrum of $[Fe_2[(SCH_2)_2NCH_2](CO)_4(PMe_3)_2]BF_4$ ([**2**]BF_4) in CD_2Cl_2 at room temperature.

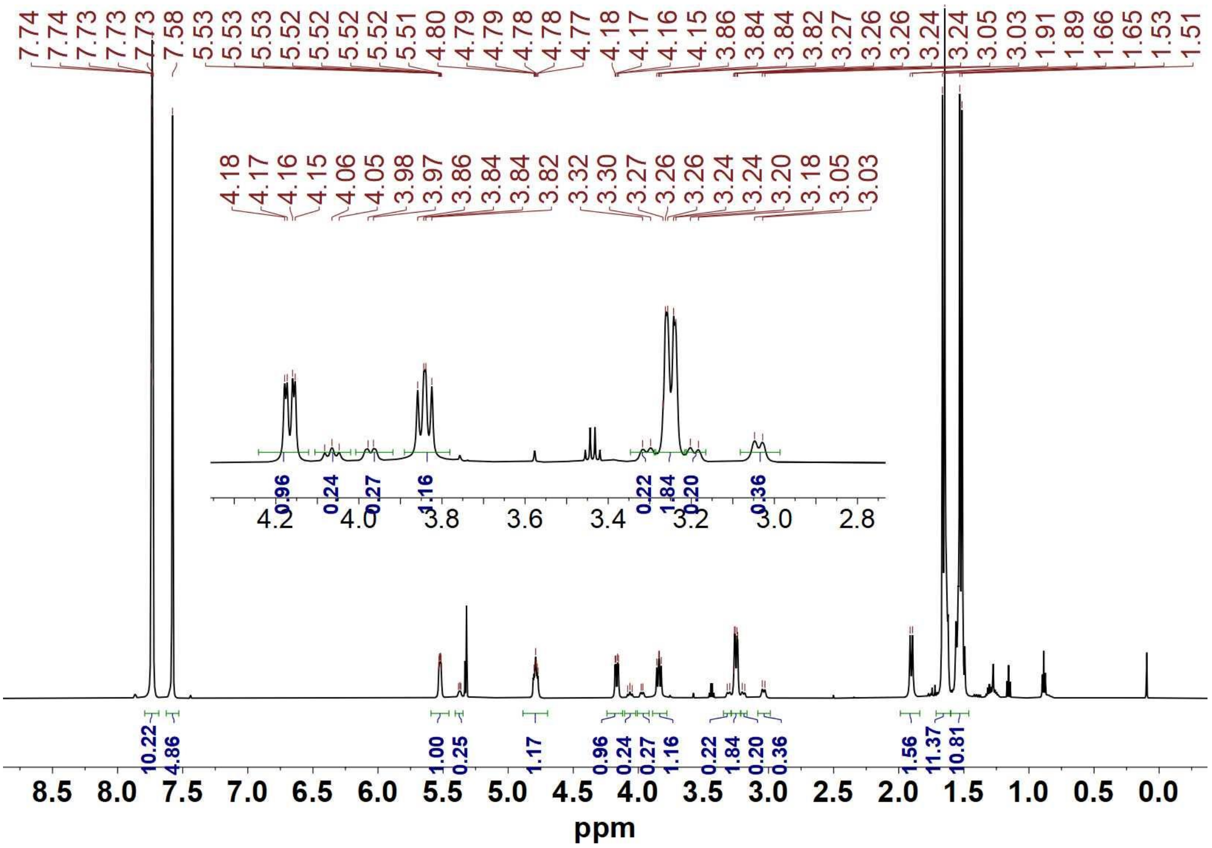

Figure S6. ^{1}H NMR spectrum of $[Fe_2[(SCH_2)_2NCH_2](CO)_4(PMe_3)_2]BAr^F{}_4$ ($[\mathbf{2}]BAr^F{}_4$) in CD_2Cl_2 at room temperature.

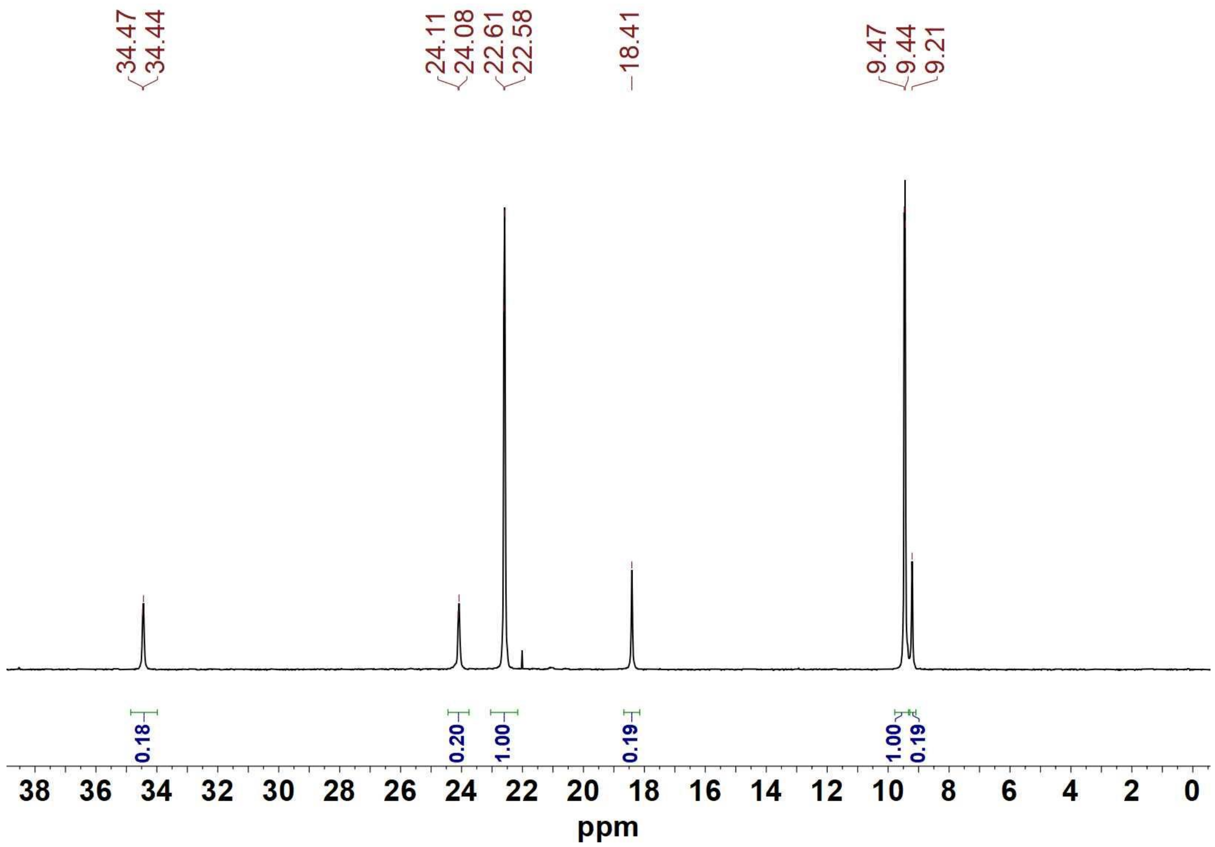

Figure S7. ^{31}P NMR spectrum of $[Fe_2[(SCH_2)_2NCH_2](CO)_4(PMe_3)_2]BAr^F{}_4$ ([**2**]$BAr^F{}_4$) in CD_2Cl_2 at room temperature.

Three isomers were detected and assigned as follows:
δ 22.59 (d, J_{PP} = 7.3 Hz), 9.45 (d, J_{PP} = 7.4 Hz), *trans*-dibasal; 34.46 (d, J_{PP} = 7.7 Hz), 24.10 (d, J_{PP} = 7.6 Hz), *cis*-dibasal; 18.41 (s), 9.21 (s), apical-basal.

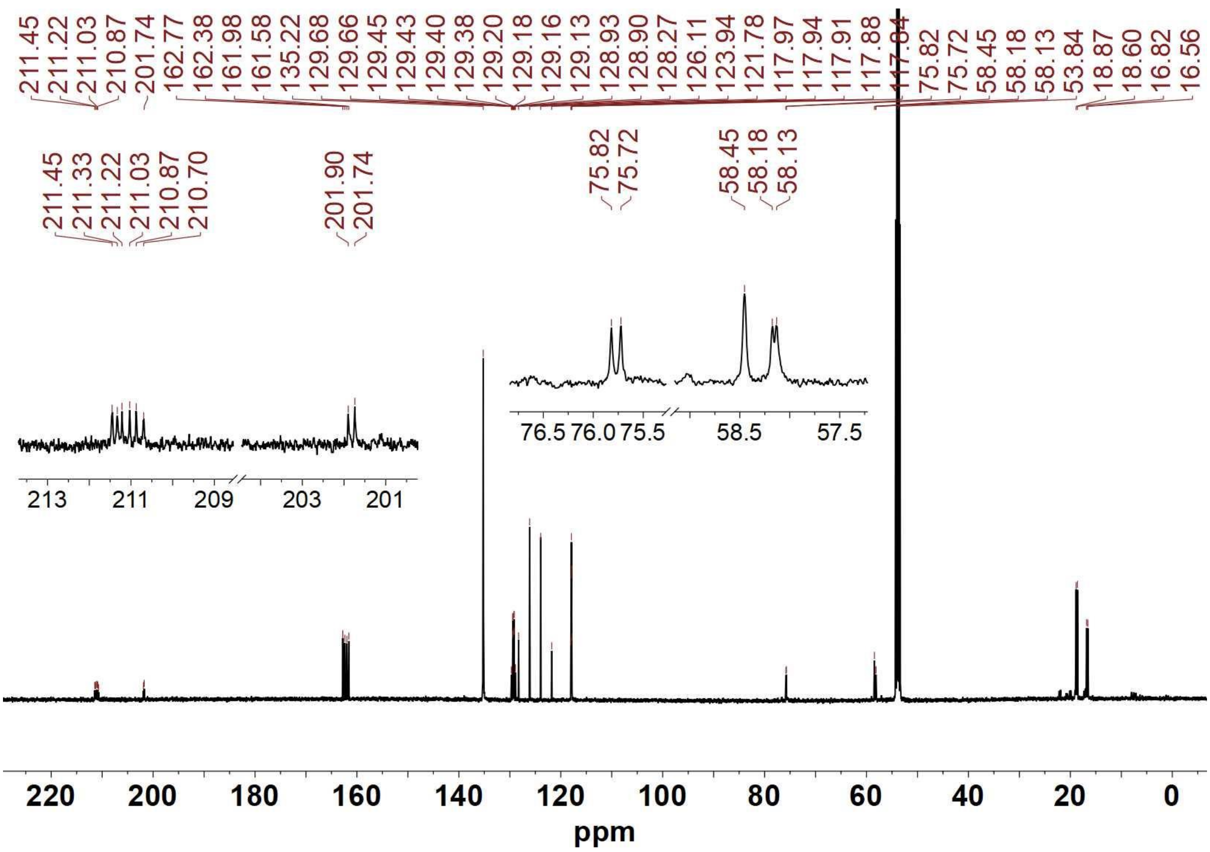

Figure S8. ^{13}C NMR spectrum of $[Fe_2[(SCH_2)_2NCH_2](CO)_4(PMe_3)_2]BAr^F{}_4$ ($[\mathbf{2}]BAr^F{}_4$) in CD_2Cl_2 at room temperature.

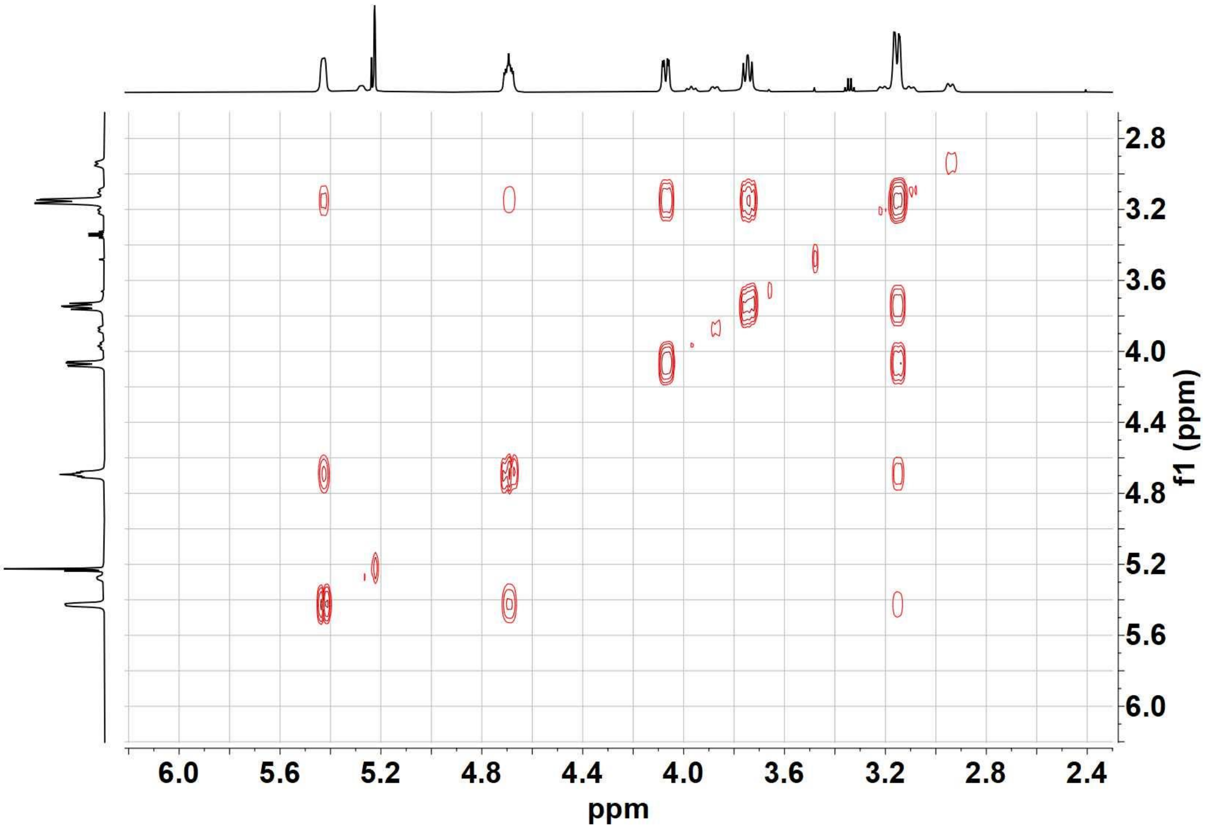

Figure S9. 2D-COSY NMR spectrum (CH_2 region) of $[Fe_2[(SCH_2)_2NCH_2](CO)_4(PMe_3)_2]BAr^F{}_4$ ([**2**]$BAr^F{}_4$) in CD_2Cl_2 at room temperature.

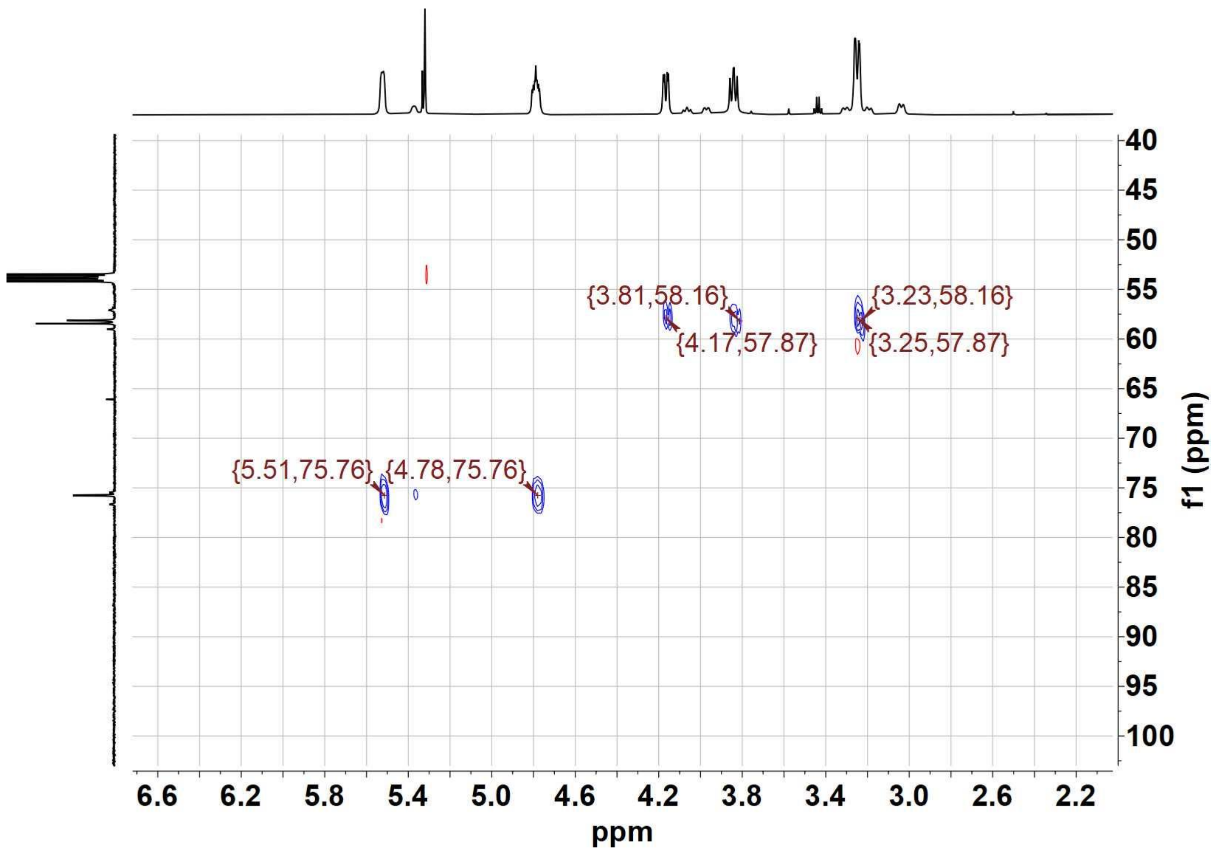

Figure S10. 2D-HSQC (^1H-^{13}C) NMR spectrum (CH_2 region) of $[Fe_2[(SCH_2)_2NCH_2](CO)_4(PMe_3)_2]BAr^F{}_4$ ([**2**]$BAr^F{}_4$) in CD_2Cl_2 at room temperature.

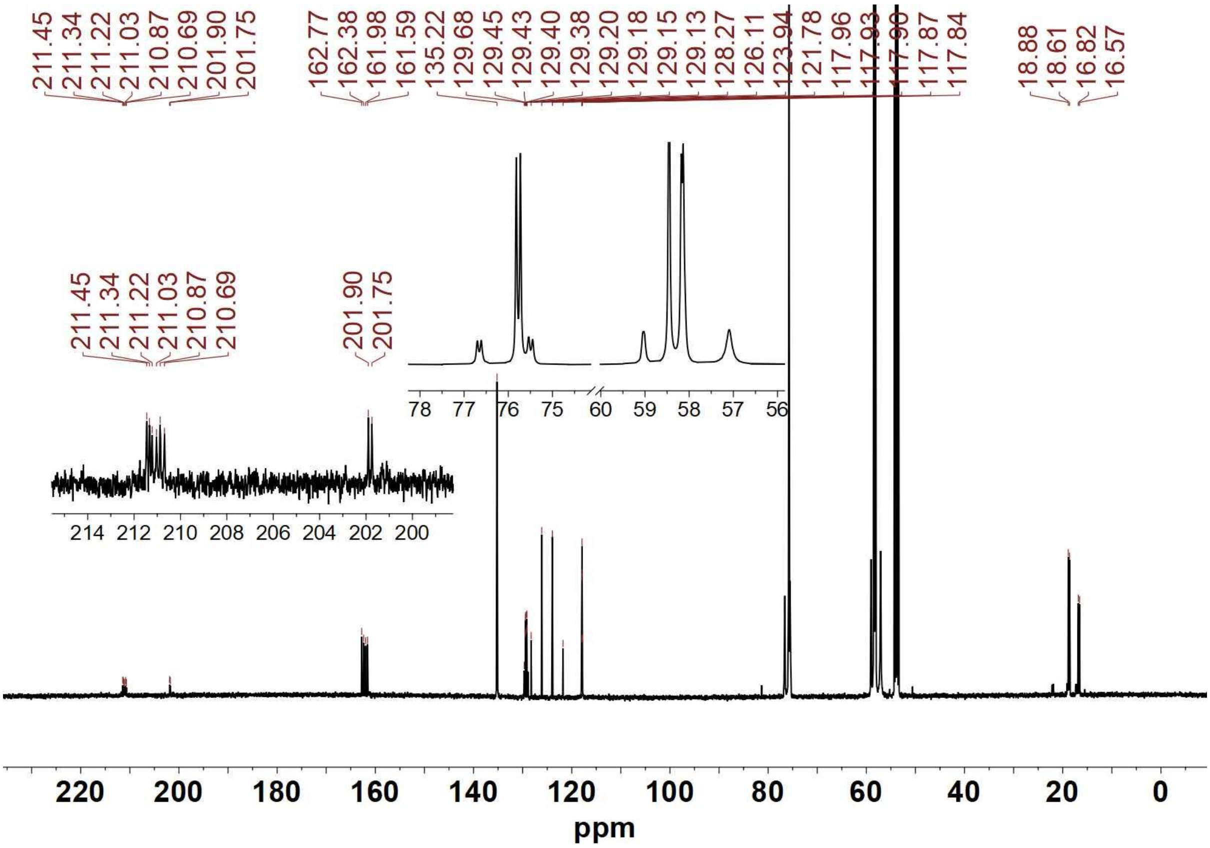

Figure S11. ^{13}C NMR spectrum of ^{13}C labeled $[Fe_2[(SCH_2)_2NCH_2](CO)_4(PMe_3)_2]BAr^F{}_4$ ([**2**]$BAr^F{}_4$) in CD_2Cl_2 at room temperature.

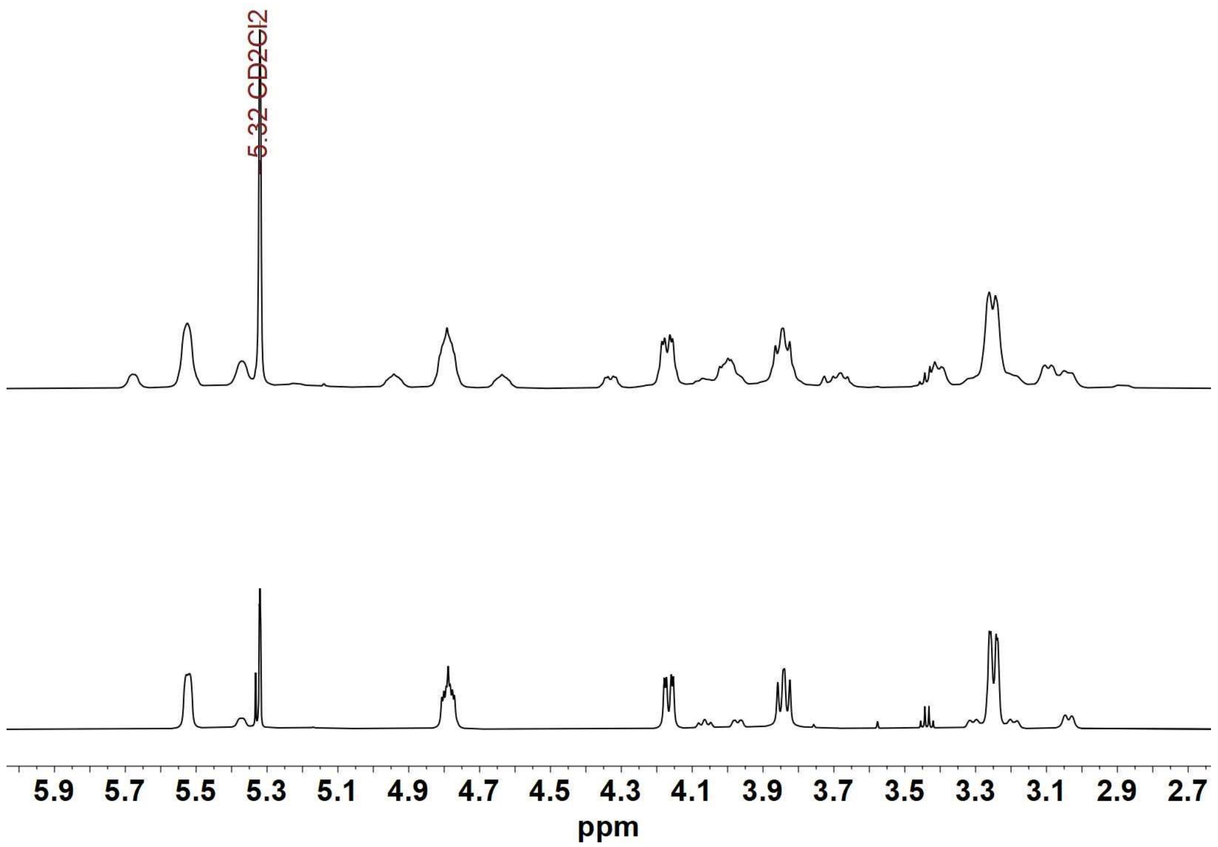

Figure S12. ^{1}H NMR spectrum (CH_2 region) of ^{13}C labeled (top) and unlabeled (bottom) $[Fe_2[(SCH_2)_2NCH_2](CO)_4(PMe_3)_2]BAr^F{}_4$ ([**2**]$BAr^F{}_4$) in CD_2Cl_2 at room temperature.

Exchange Process in [2]BAr$^F{}_4$ Determined by Spin Saturation Transfer

1D Spin-Saturation Transfer (SST) experiment (both ^{31}P and ^{1}H NMR) was used to monitor the slow exchange process in [**2**]BAr$^F{}_4$ in CD_2Cl_2 at 298 K. One signal is selectively irradiated, and the signal intensity of the other peak connected with the irradiated signal via chemical exchange is monitored.

For exchange process of

$$A \underset{k_B}{\overset{k_A}{\rightleftharpoons}} B$$

Selectively irradiation of peak A, the intensity of peak B changes following the equation below, and the exchange rate can be extracted:

$$\frac{I(t)}{I(0)} = \frac{\tau'}{\tau} \exp\left(-\frac{t}{\tau'}\right) + \frac{\tau'}{T_1}$$

$$\tau' = \frac{1}{\frac{1}{T_1} + \frac{1}{\tau}}$$

Where t is the irradiation time applied to peak A, T_1 is the longitudinal (spin-lattice) relaxation time of peak B (measured by inversion-recovery method here), τ is the life time of nuclei stays at site B, and the exchange rate $k_B = 1/\tau$.

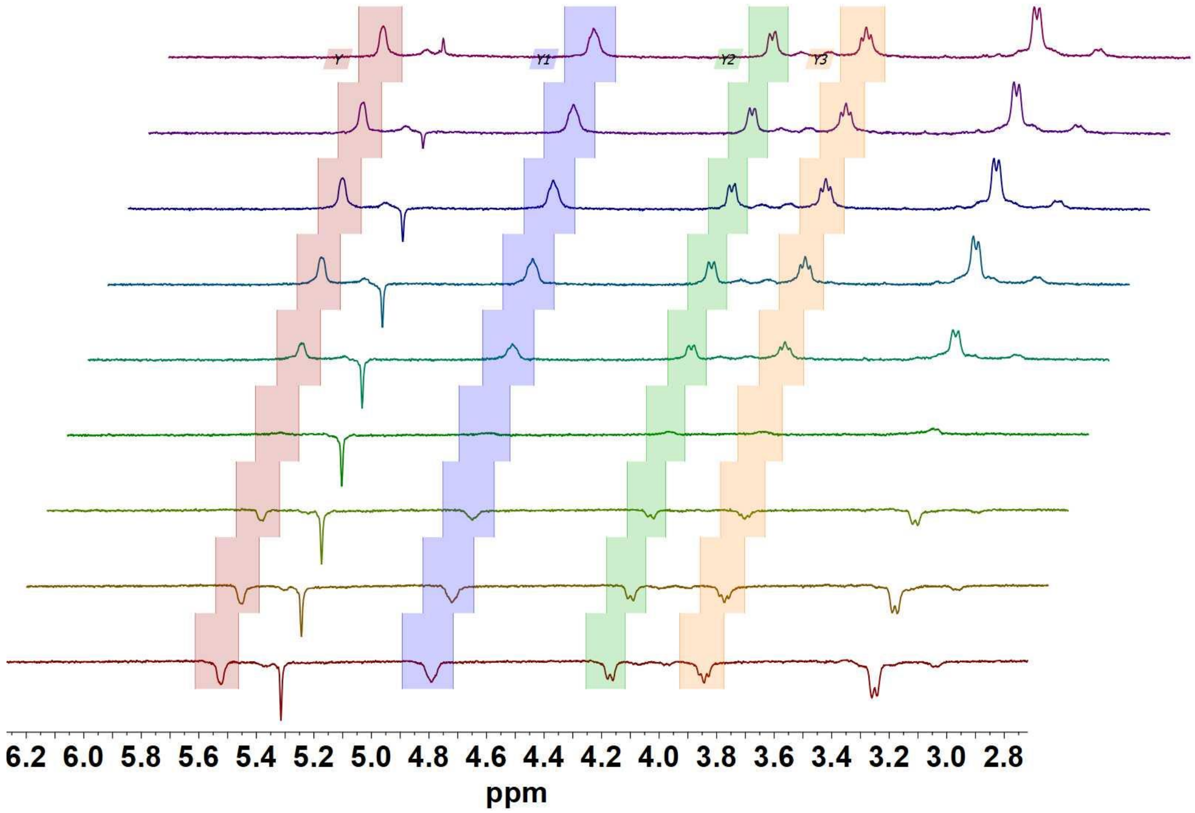

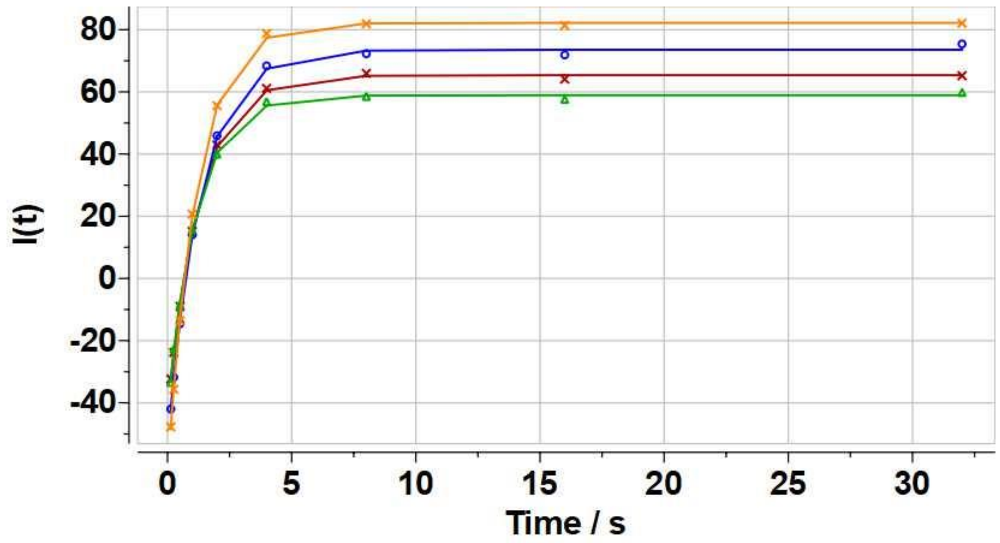

Figure S13. Stacked ^{1}H NMR spectra of [**2**]BAr$^F{}_4$ (29 mM) with different relaxation time in CD_2Cl_2 at 298 K (top); Plot of peak intensity at δ 5.52 (red), 4.79 (blue), 4.17 (green) and 3.84 (orange) *vs* relaxation time (bottom).
Fitted with three parameter exponential equation: $I_t = A + B*\exp(-t/T_1)$.

Results:
δ 5.52, T_1 *= 1.29 s; δ* 4.79, T_1 *= 1.31 s;*
δ 4.17, T_1 *= 1.17 s; δ* 3.84, T_1 *= 1.17 s.*

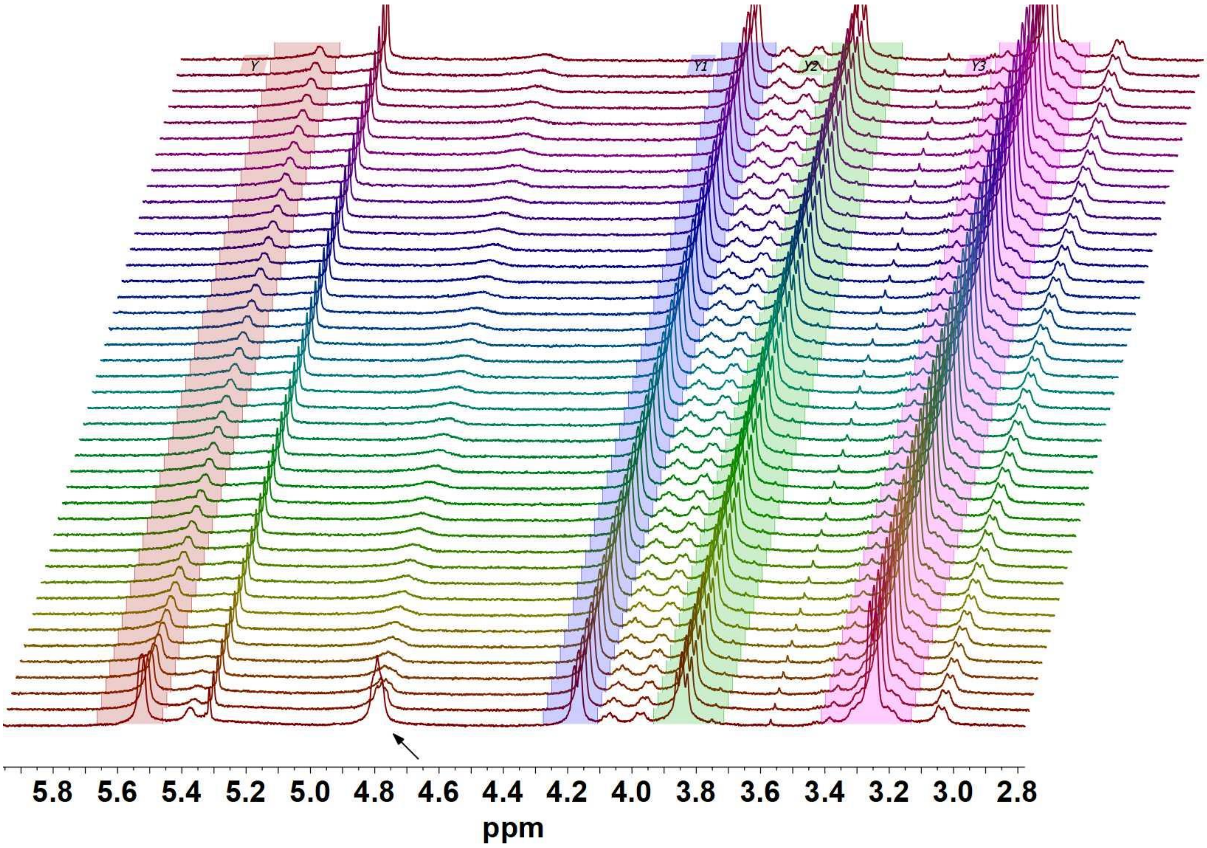

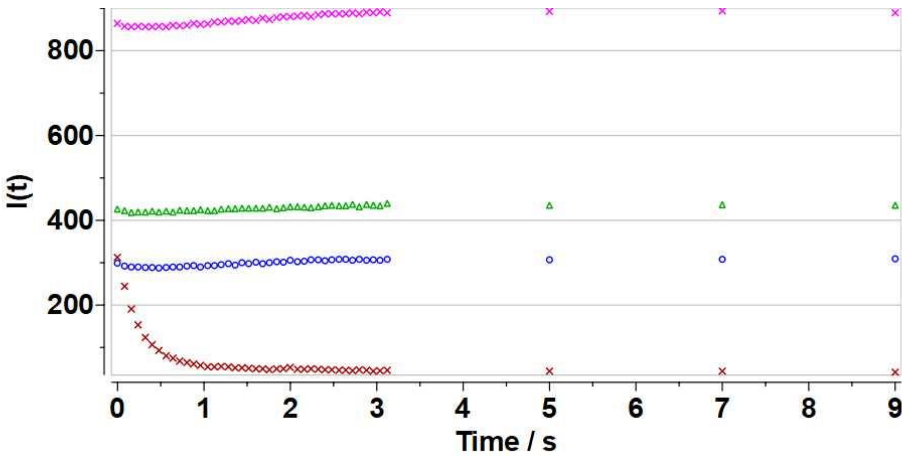

Figure S14. ^{1}H NMR spectra of [**2**]BAr^{F}_{4} (29 mM) at 298 K in CD_2Cl_2 showing the change in intensity of the proton signal at δ 5.52, 4.17, 3.84 and 3.25 upon irradiation at δ 4.79 (top), and the plot of intensity *vs* irradiation time at δ 4.79 (bottom).

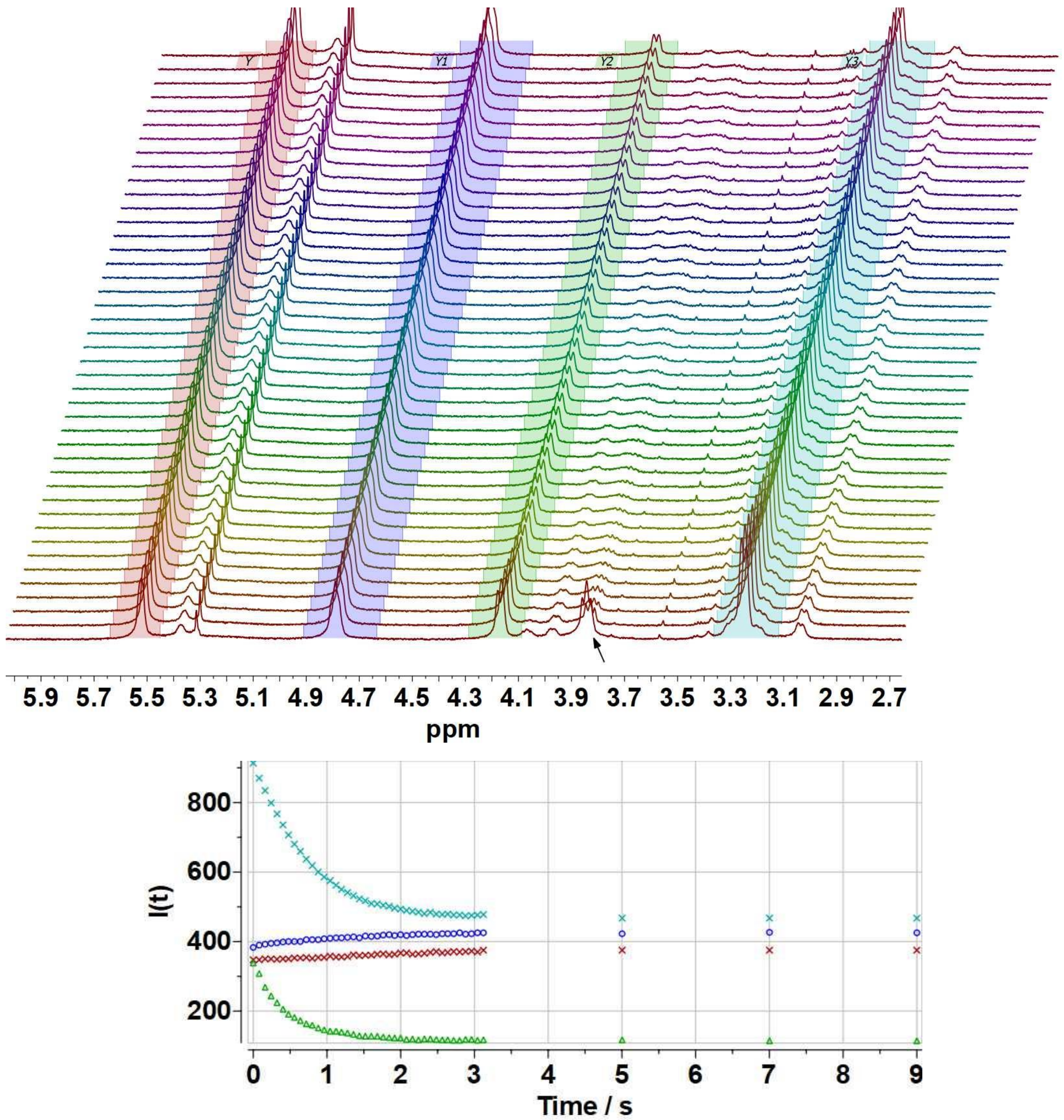

Figure S15. ^{1}H NMR spectra of [**2**]BAr$^F{}_4$ (29 mM) at 298 K in CD_2Cl_2 showing the change in intensity of the proton signal at δ 5.52, 4.79, 4.17 and 3.25 upon irradiation at δ 3.84 (top), and the plot of intensity vs irradiation time at δ 3.84 (bottom).

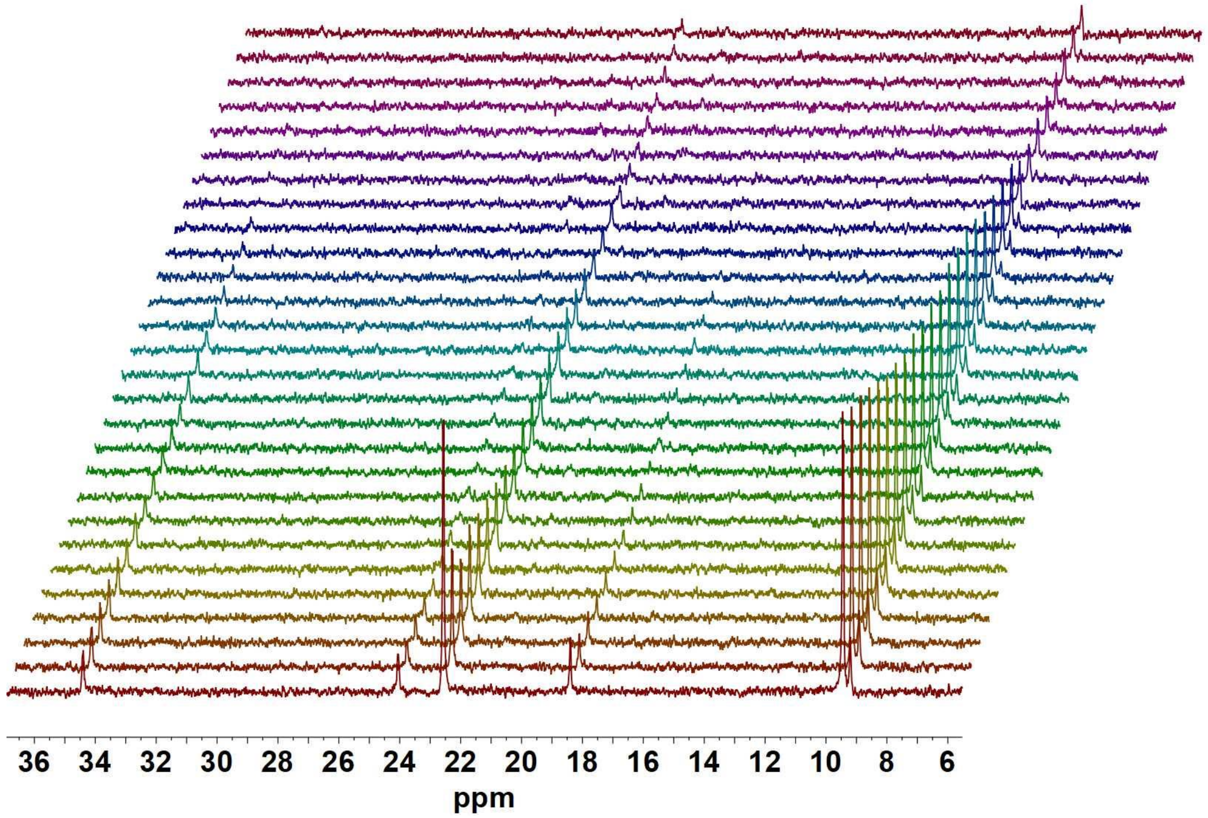

Figure S16. Spin saturation transfer spectra of [**2**]BAr$^F{}_4$ at 298 K in CD_2Cl_2, showing the decline in intensity of the phosphine signal at δ 9.45, 24.10, 18.41 and 9.21 upon irradiation at δ 22.59.

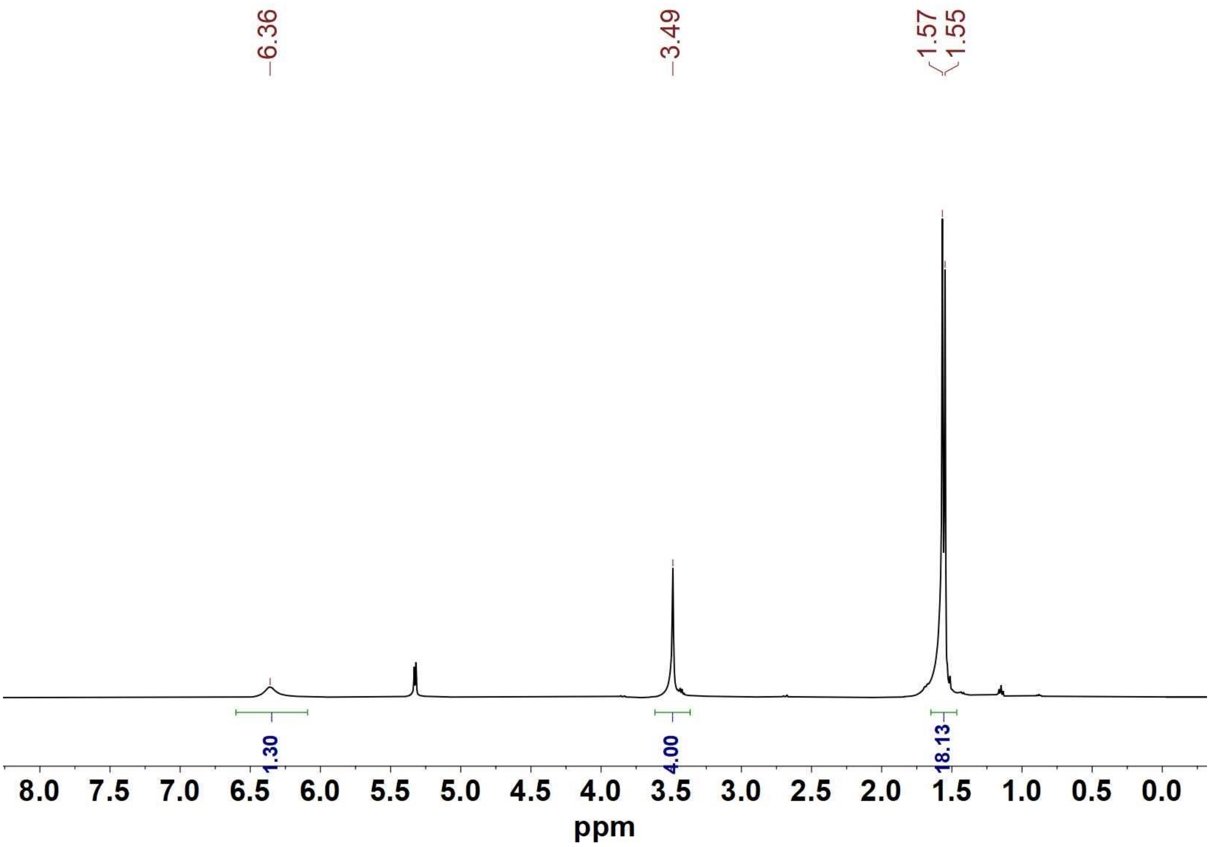

Figure S17. ^1H NMR spectrum of $[Fe_2[(SCH_2)_2NH_2](CO)_4(PMe_3)_2]BF_4$ ([**1**H]BF_4) in CD_2Cl_2 at room temperature.

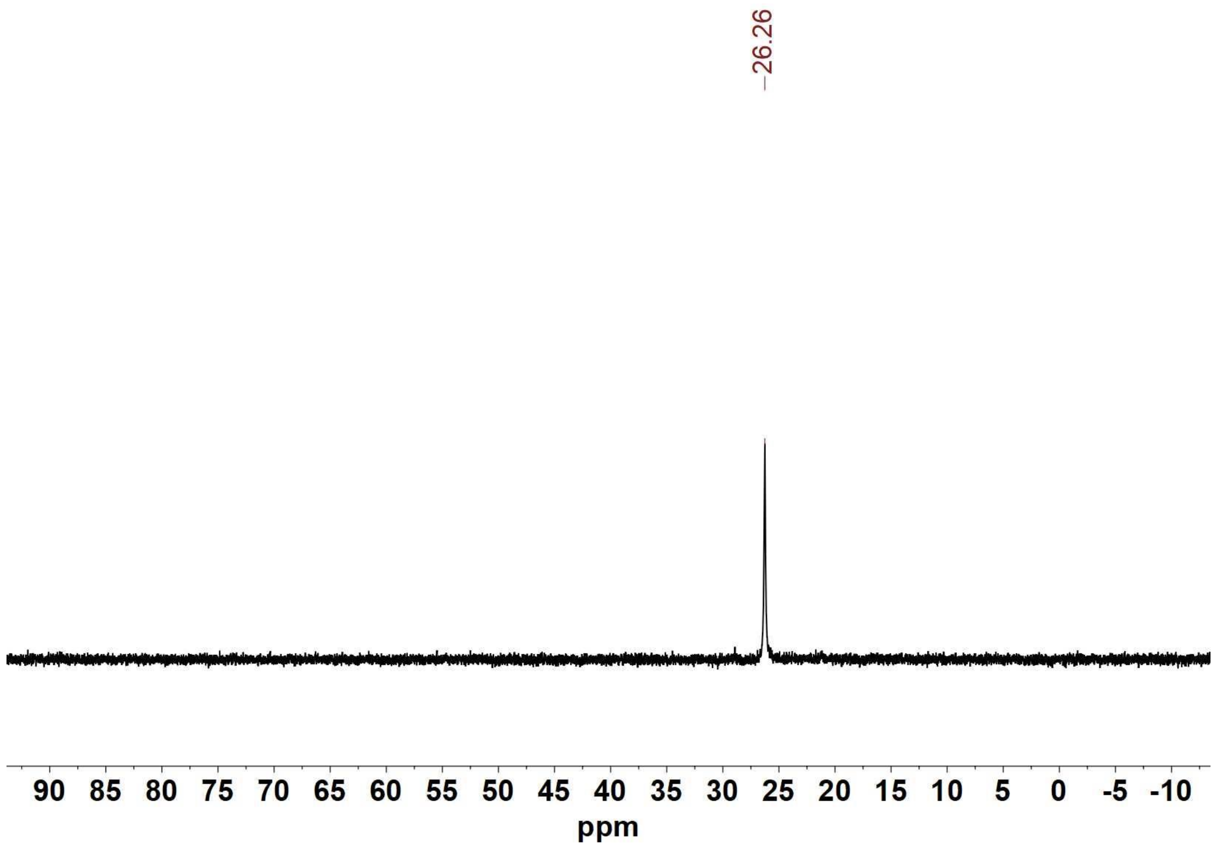

Figure S18. ^{31}P NMR spectrum of $[Fe_2[(SCH_2)_2NH_2](CO)_4(PMe_3)_2]BF_4$ ([**1**H]BF_4) in CD_2Cl_2 at room temperature.

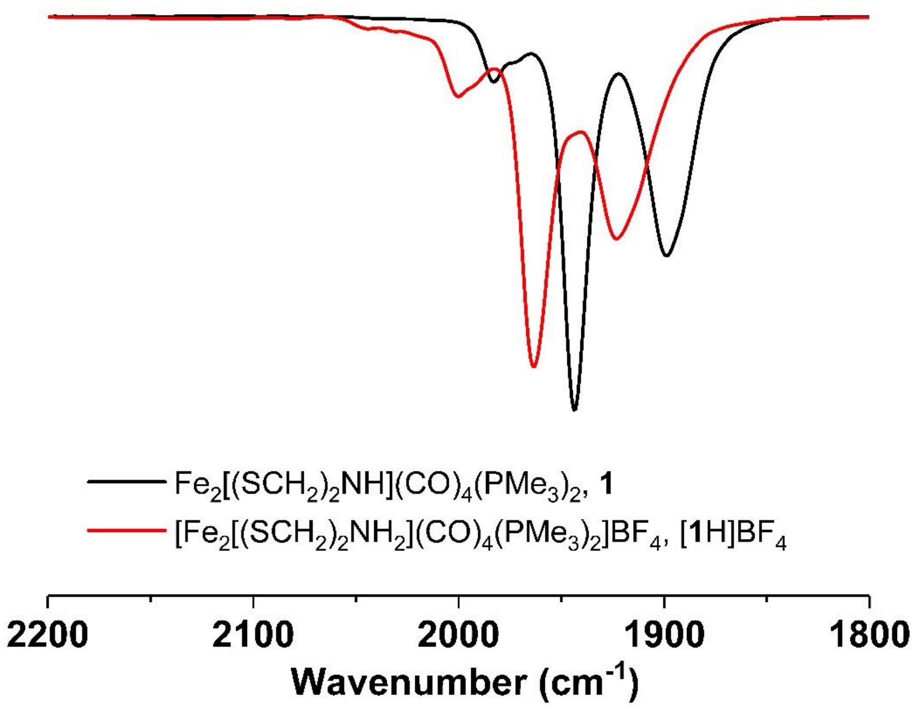

Figure S19. IR spectra of $Fe_2[(SCH_2)_2NH](CO)_4(PMe_3)_2$ (**1**) and $[Fe_2[(SCH_2)_2NH_2](CO)_4(PMe_3)_2]BF_4$ ([**1**H]BF_4) in CH_2Cl_2 solution.

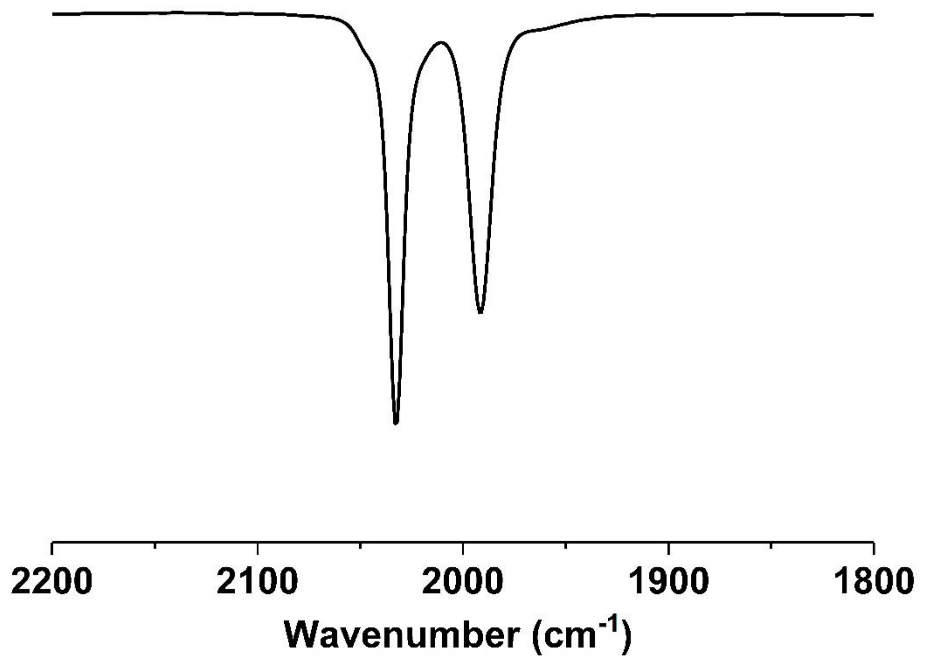

Figure S20. IR spectrum of $[HFe_2[(SCH_2)_2NH](CO)_4(PMe_3)_2]BAr^F{}_4$ ([H**1**]$BAr^F{}_4$) in CH_2Cl_2 solution.

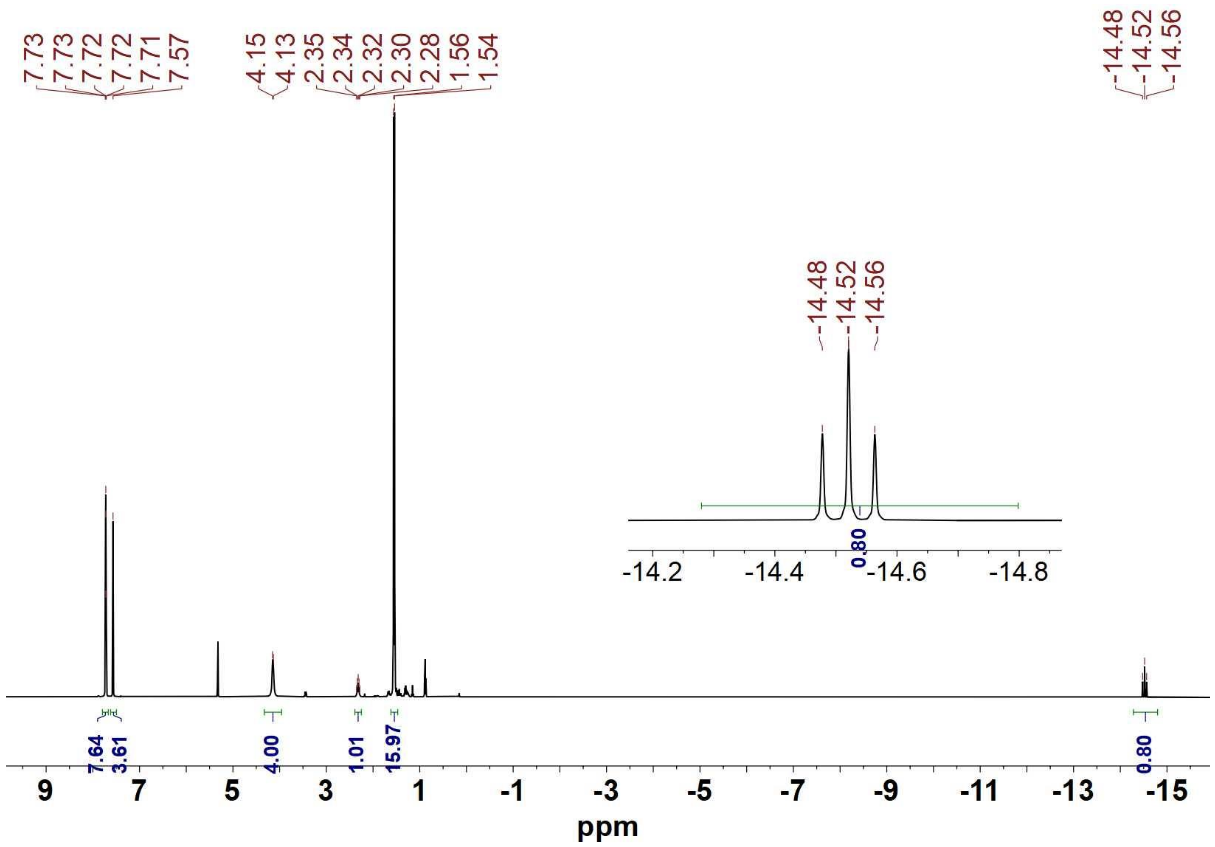

Figure S21. ^{1}H NMR spectrum of $[HFe_2[(SCH_2)_2NH](CO)_4(PMe_3)_2]BAr^F{}_4$ ($[H\mathbf{1}]BAr^F{}_4$) in CD_2Cl_2 at room temperature.

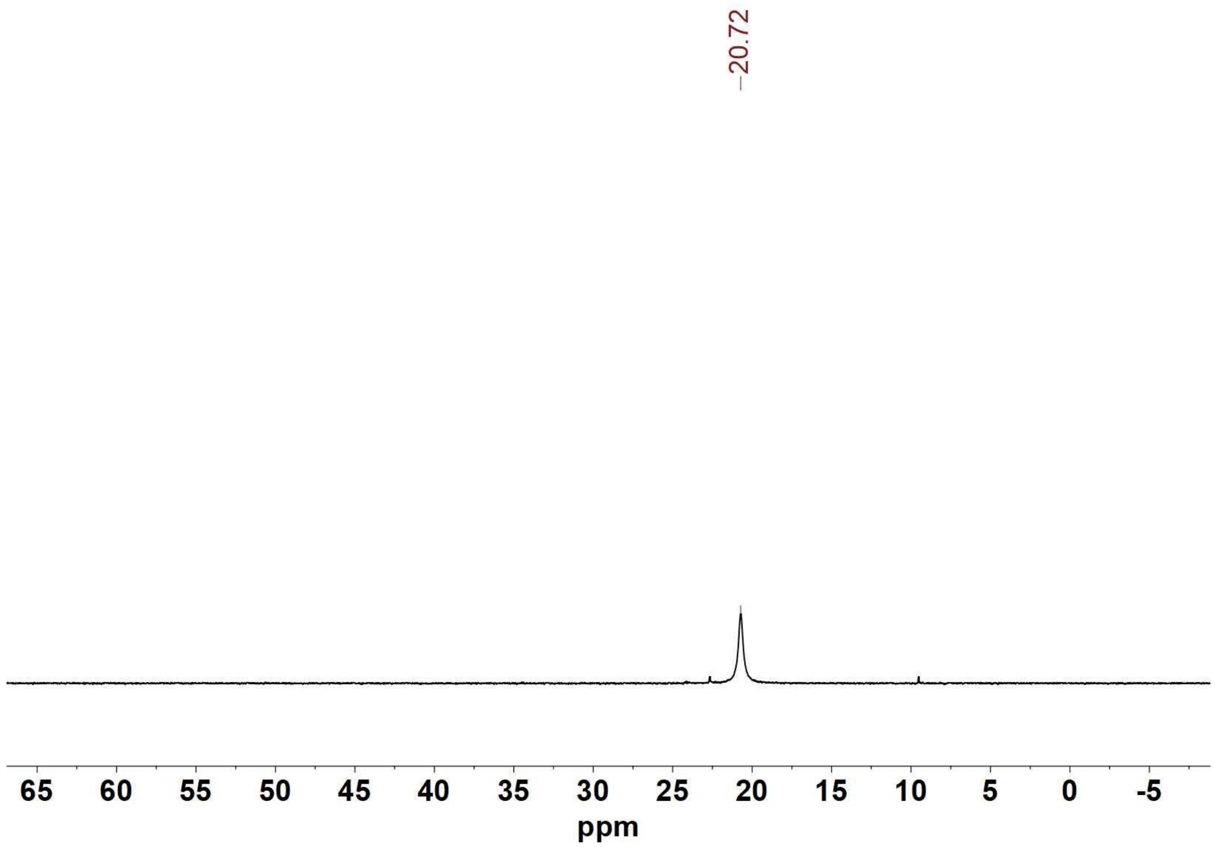

Figure S22. ^{31}P NMR spectrum of $[HFe_2[(SCH_2)_2NH](CO)_4(PMe_3)_2]BAr^F{}_4$ ($[H\mathbf{1}]BAr^F{}_4$) in CD_2Cl_2 at room temperature.

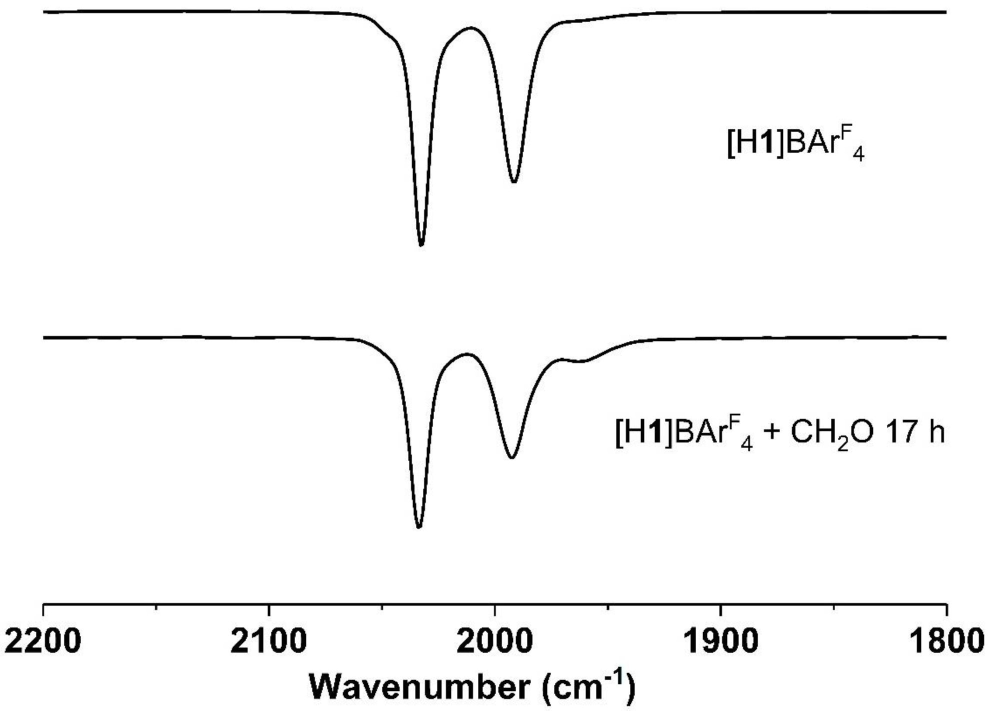

Figure S23. IR spectrum for reaction of $[HFe_2[(SCH_2)_2NH](CO)_4(PMe_3)_2]BAr^F{}_4$ ([H**1**]$BAr^F{}_4$) with 3 equiv paraformaldehyde in CH_2Cl_2 solution.

*The result indicates the reaction of hydride tautomer [H**1**]$^+$ with paraformaldehyde does not give [**2**]$^+$, which highlight the role of the ammonium center in the conversion of **1** to [**2**]$^+$.*

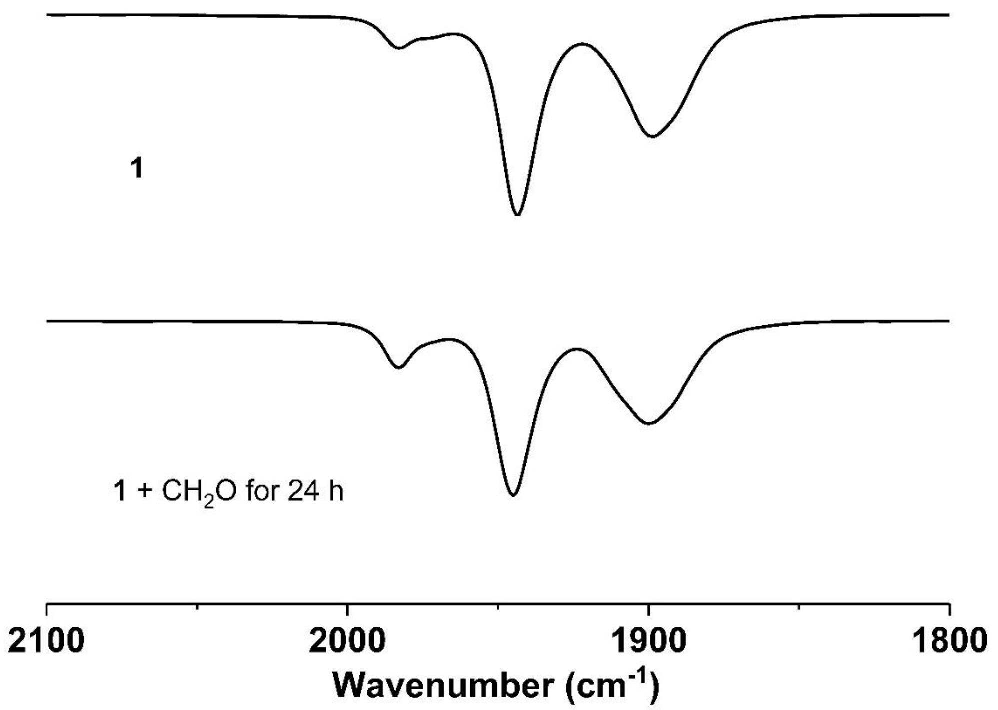

Figure S24. IR spectrum of $Fe_2[(SCH_2)_2NH](CO)_4(PMe_3)_2$ (**1**) and **1** + CH_2O for 24 h in CH_2Cl_2 solution.

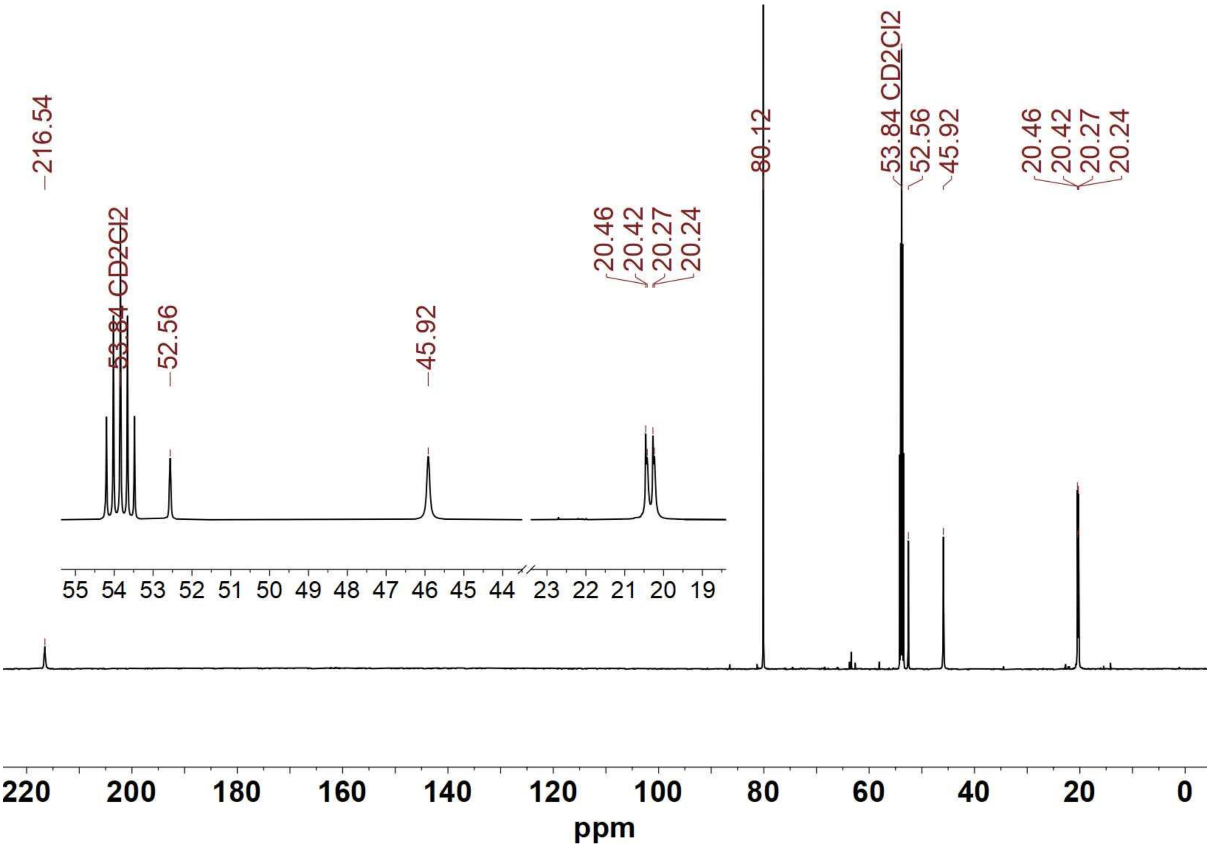

Figure S25. ^{13}C NMR spectrum for the reaction of $[Fe_2(SCH_2)_2NH(CO)_4(PMe_3)_2]$ with 0.25 equiv $^{13}CH_2O$ in CD_2Cl_2 at room temperature.

Assignments:
δ 80.11 (enriched with ^{13}C) and 52.56 ($[Fe_2[(SCH_2)_2N^{13}CH_2OH](CO)_4(PMe_3)_2]$);
δ 45.91 ($[Fe_2[(SCH_2)_2NH](CO)_4(PMe_3)_2]$).

In order to exam whether there is exchange during the formation of N-CH_2-OH, the reaction of $[Fe_2[(SCH_2)_2NH](CO)_4(PMe_3)_2]$ with 0.25 equiv $^{13}CH_2O$ in CD_2Cl_2 was checked at room temperature. According to the ^{13}C NMR determined after 72 h, two new peaks appeared at δ 80.11 (enriched with ^{13}C) and δ 52.56, which could be assigned to the 1 : 1 adduct of $[Fe_2[(SCH_2)_2N^{13}CH_2OH](CO)_4(PMe_3)_2]$. However, the peak at δ 45.91 corresponding to the SCH_2 of $Fe_2[(SCH_2)_2NH](CO)_4(PMe_3)_2$ has not been enriched, indicating no exchange during this process.

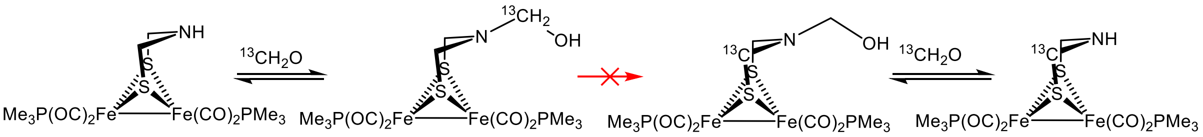

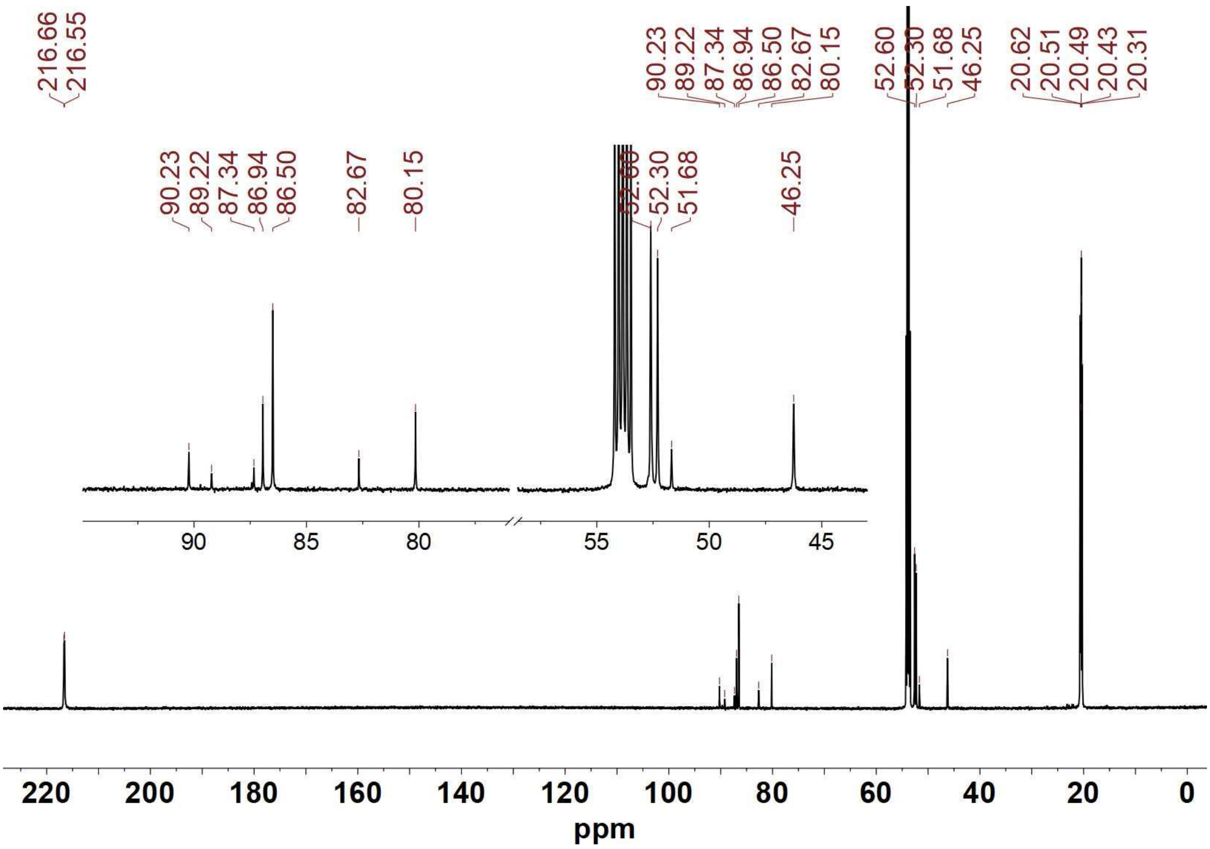

Figure S26. ^{13}C NMR spectrum for the reaction of $[Fe_2[(SCH_2)_2NH](CO)_4(PMe_3)_2]$ with 1 equiv CH_2O in CD_2Cl_2 at room temperature.

Assignments:

δ 80.15 and 52.60 ($[Fe_2[(SCH_2)_2NCH_2OH](CO)_4(PMe_3)_2]$);

δ 90.23, 89.22, 87.34, 86.94, 86.50, 82.67 for $-(CH_2)_n-OH$ and 52.30, 51.68 for SCH_2 ($[Fe_2[(SCH_2)_2N(CH_2)_nOH](CO)_4(PMe_3)_2]$);

δ 45.91 ($[Fe_2[(SCH_2)_2NH](CO)_4(PMe_3)_2]$).

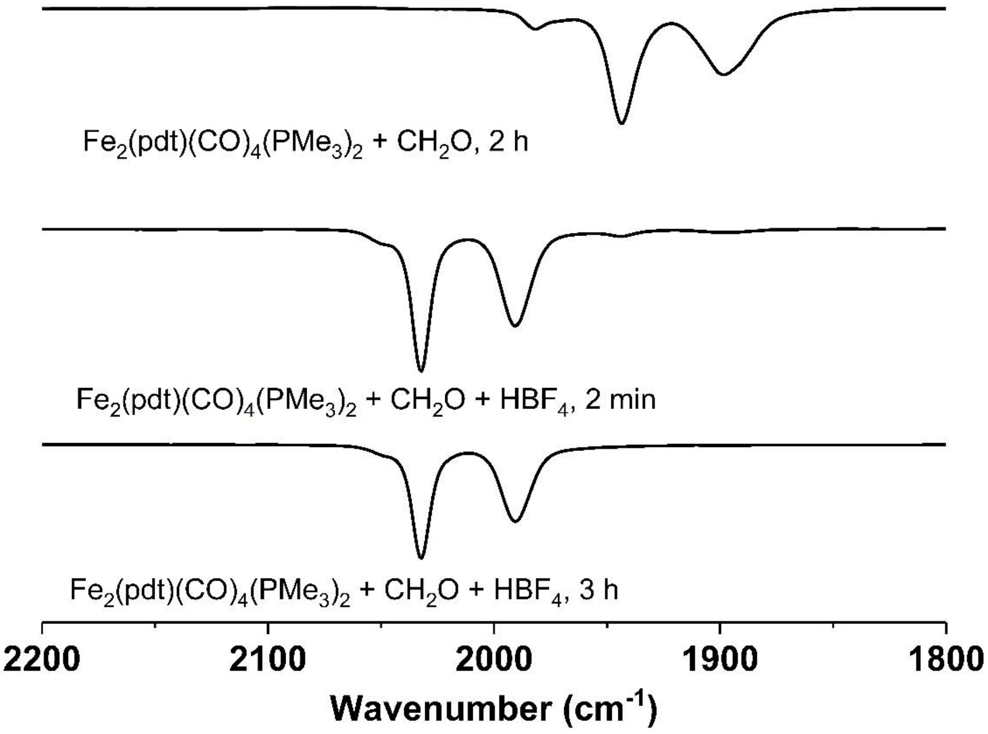

Figure S27. IR spectra for reaction of [Fe_2(pdt)$(CO)_4(PMe_3)_2$] + CH_2O + HBF_4 in CH_2Cl_2 solution.

According to the IR spectra, no reaction is evident when [Fe_2(pdt)$(CO)_4(PMe_3)_2$] was treated with CH_2O. In the presence of 1 equiv HBF_4, bridging hydride [HFe_2(pdt)$(CO)_4(PMe_3)_2$]BF_4 was formed as the protonation product.

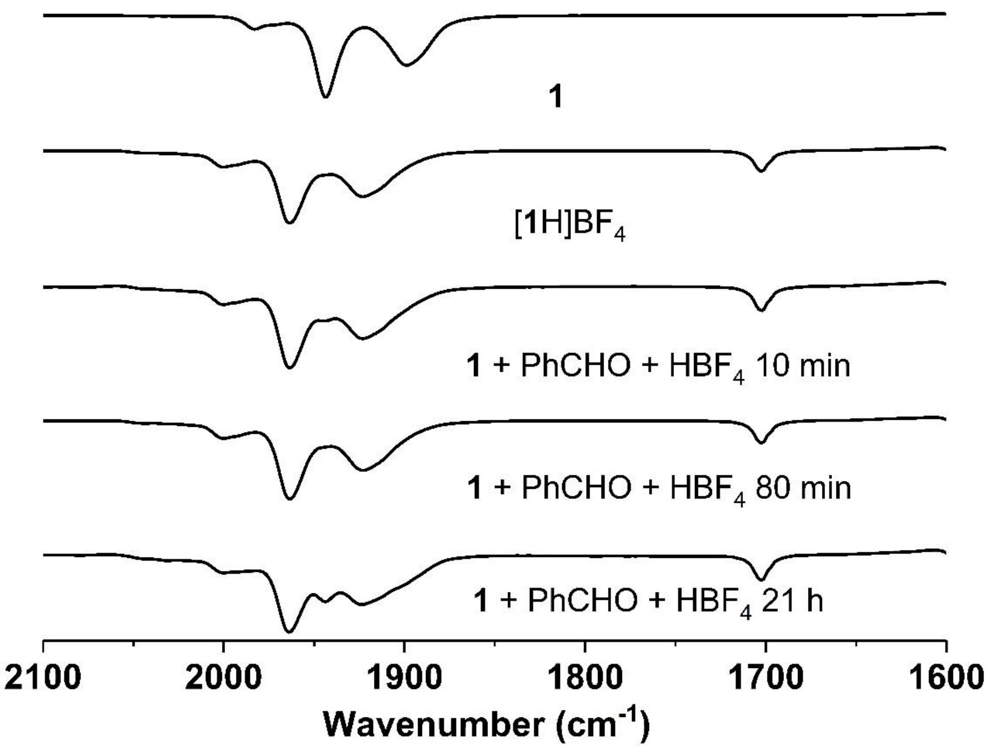

Figure S28. IR spectra for reaction of $Fe_2[(SCH_2)_2NH](CO)_4(PMe_3)_2$ (**1**) + PhCHO + $HBF_4{\cdot}Et_2O$ in CH_2Cl_2 solution. The band at 1702 cm^{-1} is assigned to C=O vibration of PhCHO.

According to the IR spectra, no reaction is evident when ***1*** *was treated with PhCHO. In the presence of* HBF_4*, (*$[Fe_2[(SCH_2)_2NH_2](CO)_4(PMe_3)_2]BF_4$*) ([****1****H]*$BF_4$*) was formed as the protonation product.*

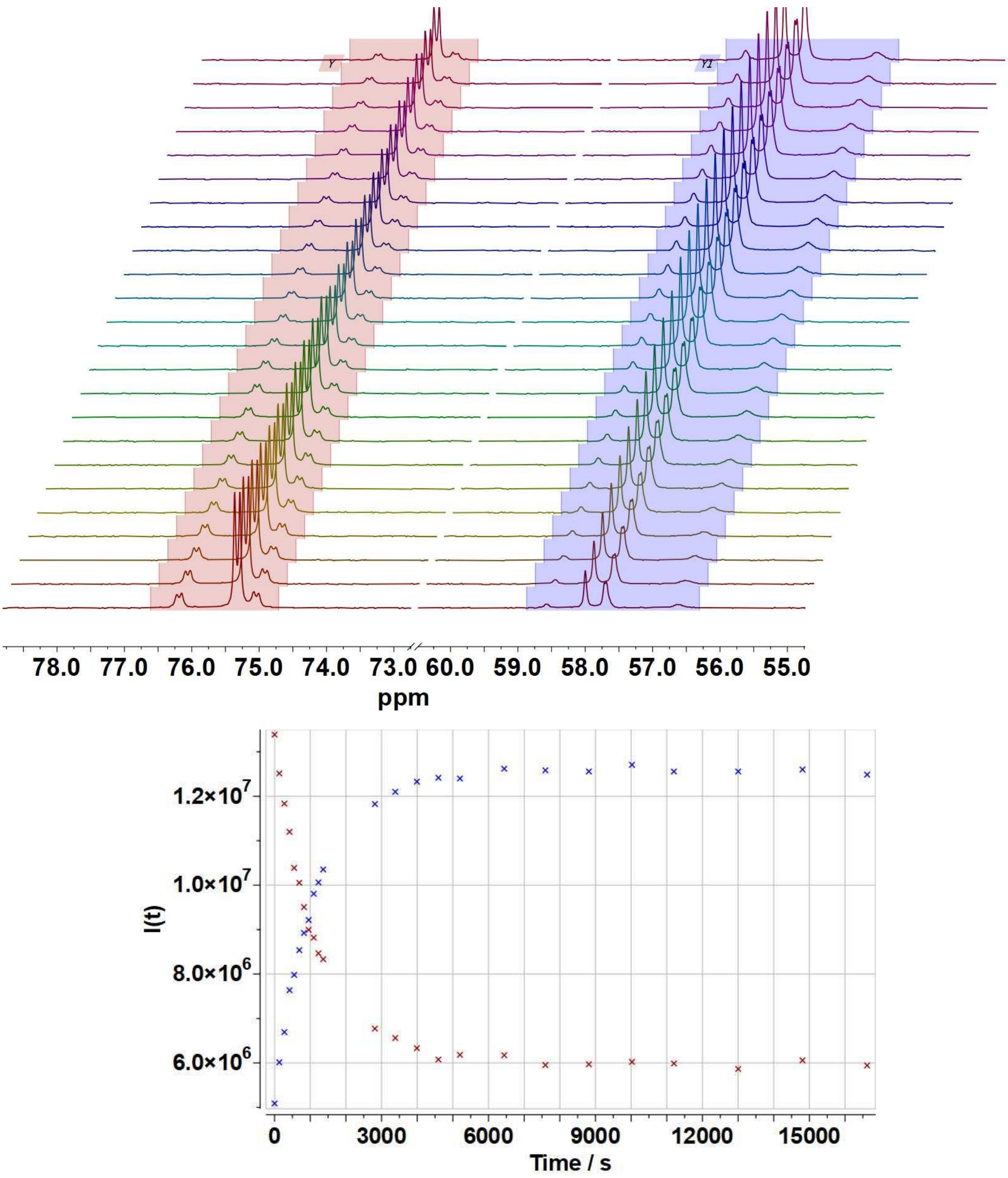

Figure S29. ^{13}C NMR spectrum (CH_2 region) for the reaction of $[Fe_2[(SCH_2)_2NH](CO)_4(PMe_3)_2]$ (47 mM) + $^{13}CH_2O$ + $HBAr^F_4$ (1 : 1 : 1) over time in CD_2Cl_2 at 296 K (top); Plot of peak intensity *vs* time, showing the decline in intensity of the signal at δ 76.66, 75.77, 75.49 (brown), and the increase in intensity of the signal at δ 59.03, 58.46, 58.16, 57.09 (blue) (bottom). The exchange process reaches equilibrium after ~90 minutes.

For the equilibrium

$$A \underset{k_{-1}}{\overset{k_1}{\rightleftharpoons}} B$$

k_1 is the rate coefficient for the reaction that consumes A; k_{-1} is the rate coefficient for the backwards reaction, then

$$\frac{k_1}{k_{-1}} = \frac{[B]_e}{[A]_e}$$

$[A]_e$ and $[B]_e$ are the concentrations of A and B at equilibrium, respectively. $[A]_0$ is the initial concentration of reactant A, $[A]_t$ is the concentration of A at time t, then

$$\ln\left(\frac{I_0 - I_e}{I_t - I_e}\right) = \ln\left(\frac{[A]_0 - [A]_e}{[A]_t - [A]_e}\right) = (k_1 + k_{-1})t$$

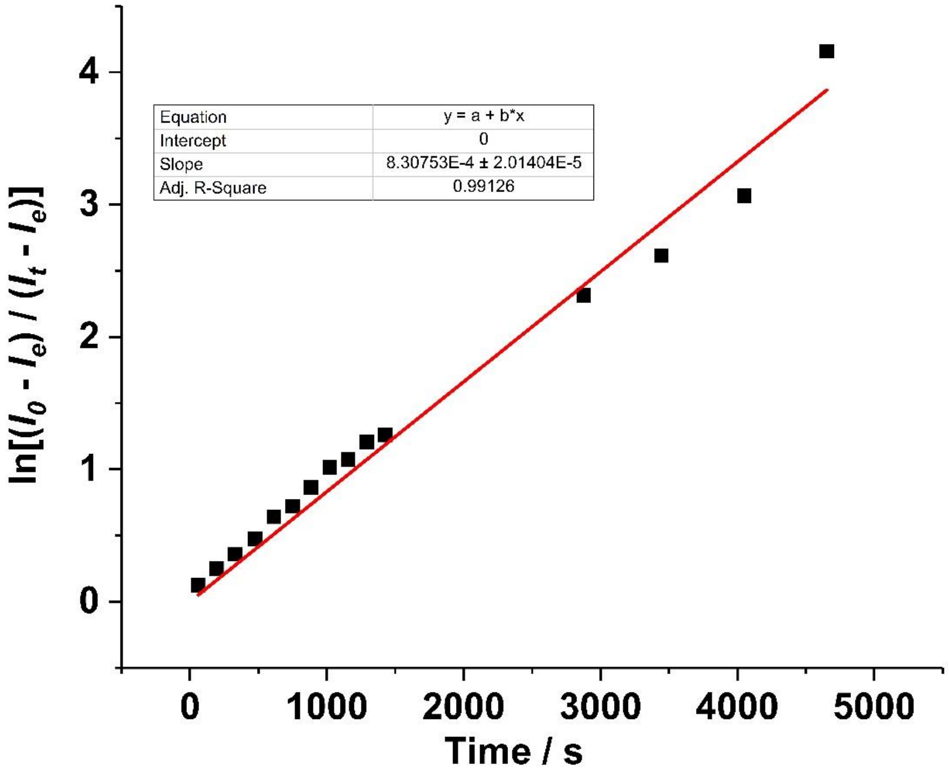

Figure S30. Plot of $\ln[(I_0 - I_e)/(I_t - I_e)]$ *vs* reaction time ($[\mathbf{2}]^+_0$ = 47 mM). *Fitting equation*: $\ln[(I_0 - I_e)/(I_t - I_e)] = (k_1 + k_{-1})t$. *Results:* $k_1 + k_{-1} = 8.31 \times 10^{-4} \pm 2.01 \times 10^{-5}\ s^{-1}$. The linear plot indicates the kinetics of exchange are first order in the concentration of $[\mathbf{2}]^+$, which points to an intramolecular process.

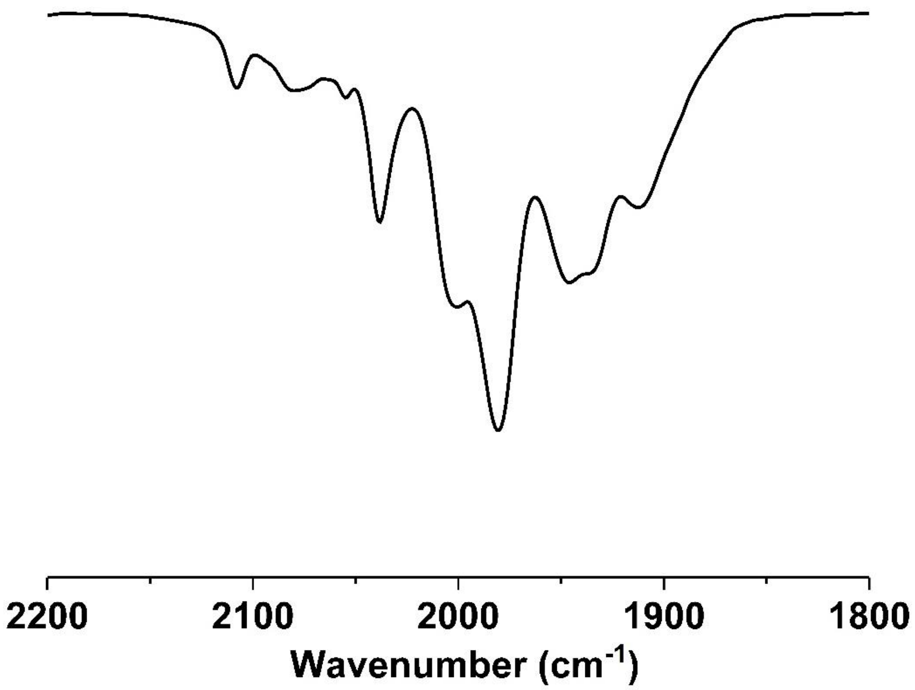

Figure S31. IR spectrum of crude $Et_4N[Fe_2[(SCH_2)_2NCH_2CN](CN)(CO)_5]$ (Et_4N[**4**]) in CH_3CN solution.

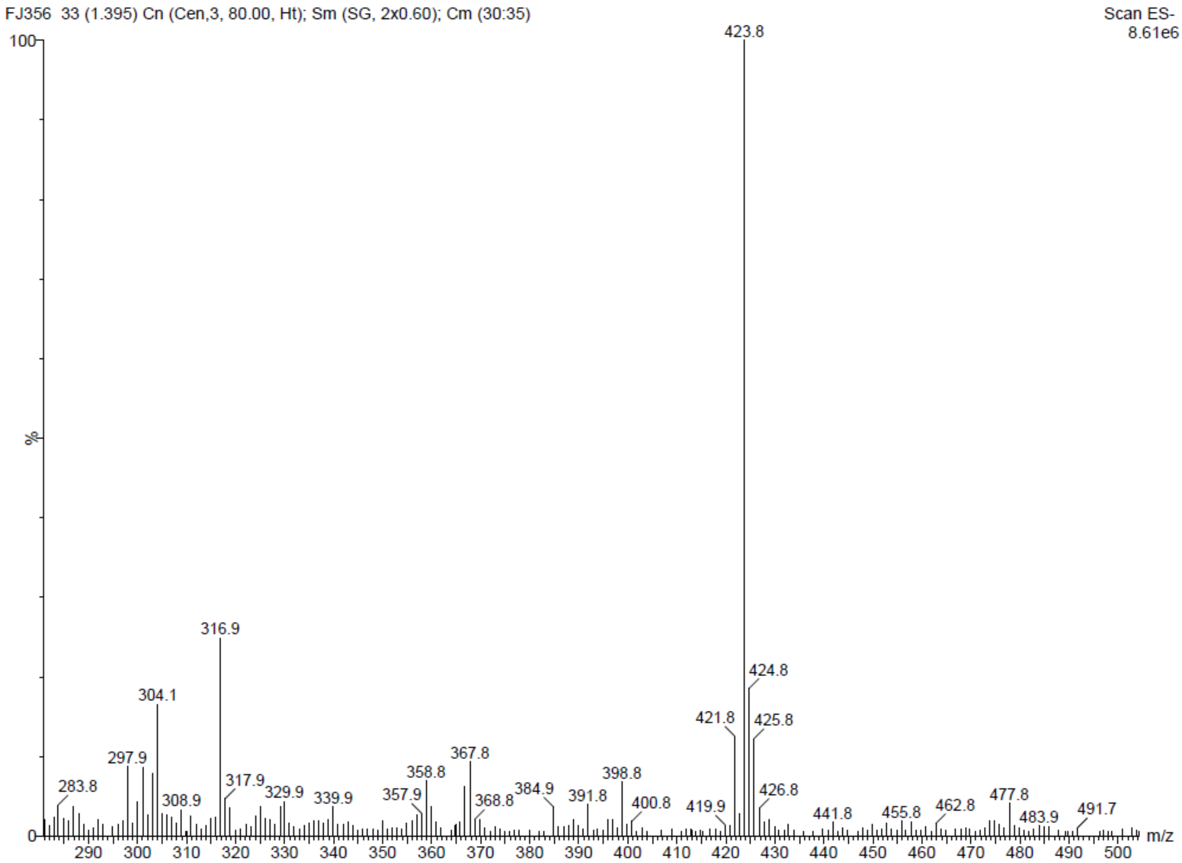

Figure S32. ESI-MS spectrum of $Et_4N[Fe_2[(SCH_2)_2NCH_2CN](CN)(CO)_5]$ (Et_4N[**4**]) in CH_3CN solution.

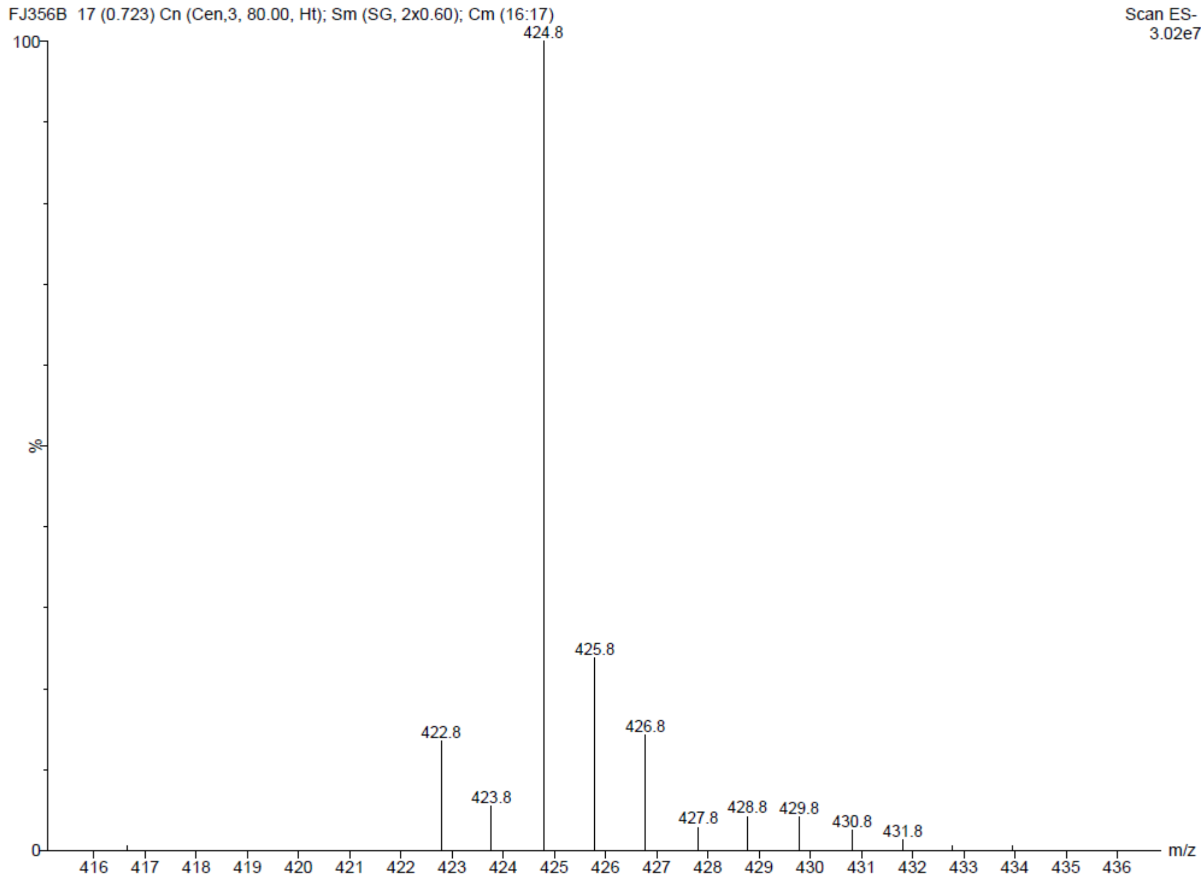

Figure S33. ESI-MS spectrum of ^{13}C labeled $Et_4N[Fe_2[(SCH_2)_2NCH_2CN](CN)(CO)_5]$ (Et_4N[**4**]) in CH_3CN solution.

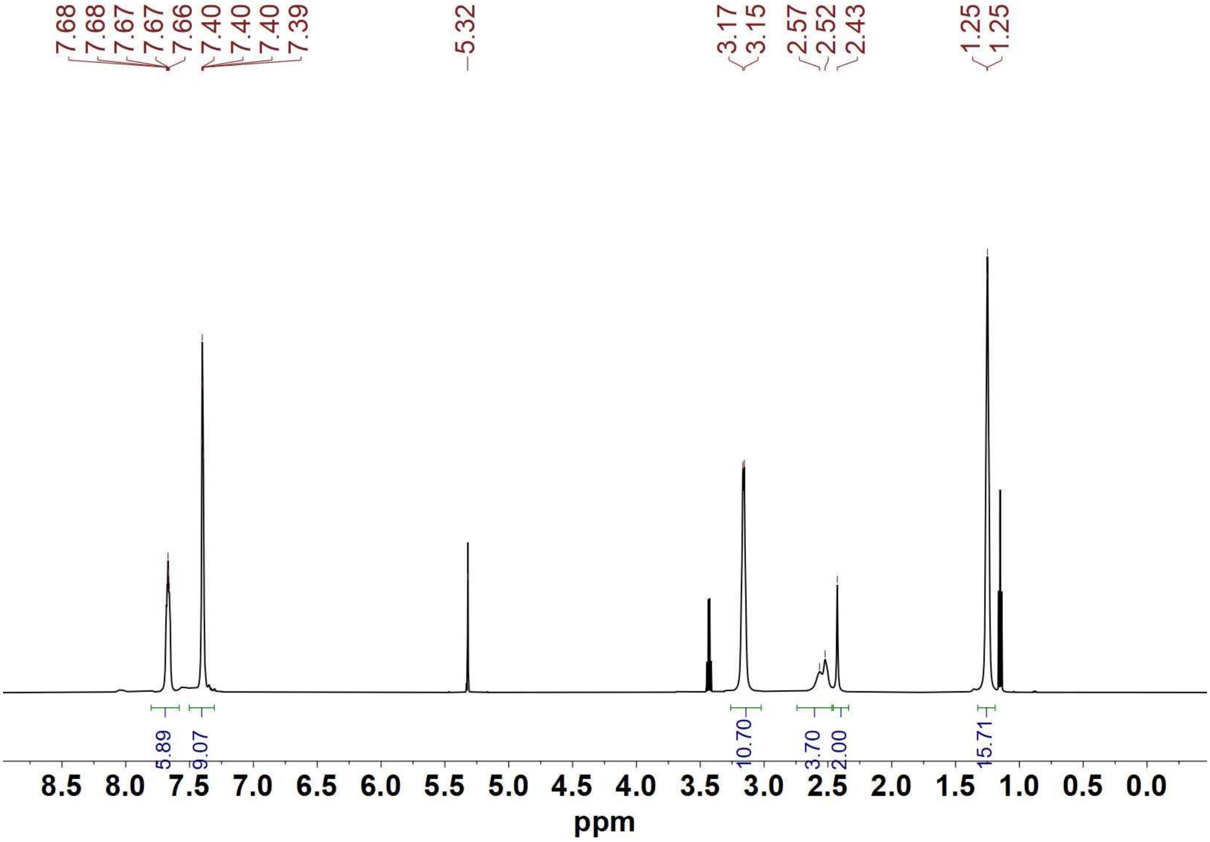

Figure S34. ^{1}H NMR spectrum of $Et_4N[Fe_2[(SCH_2)_2NCH_2CN](CN)(CO)_4PPh_3]$ (Et_4N[**5**]) in CD_2Cl_2 at room temperature.

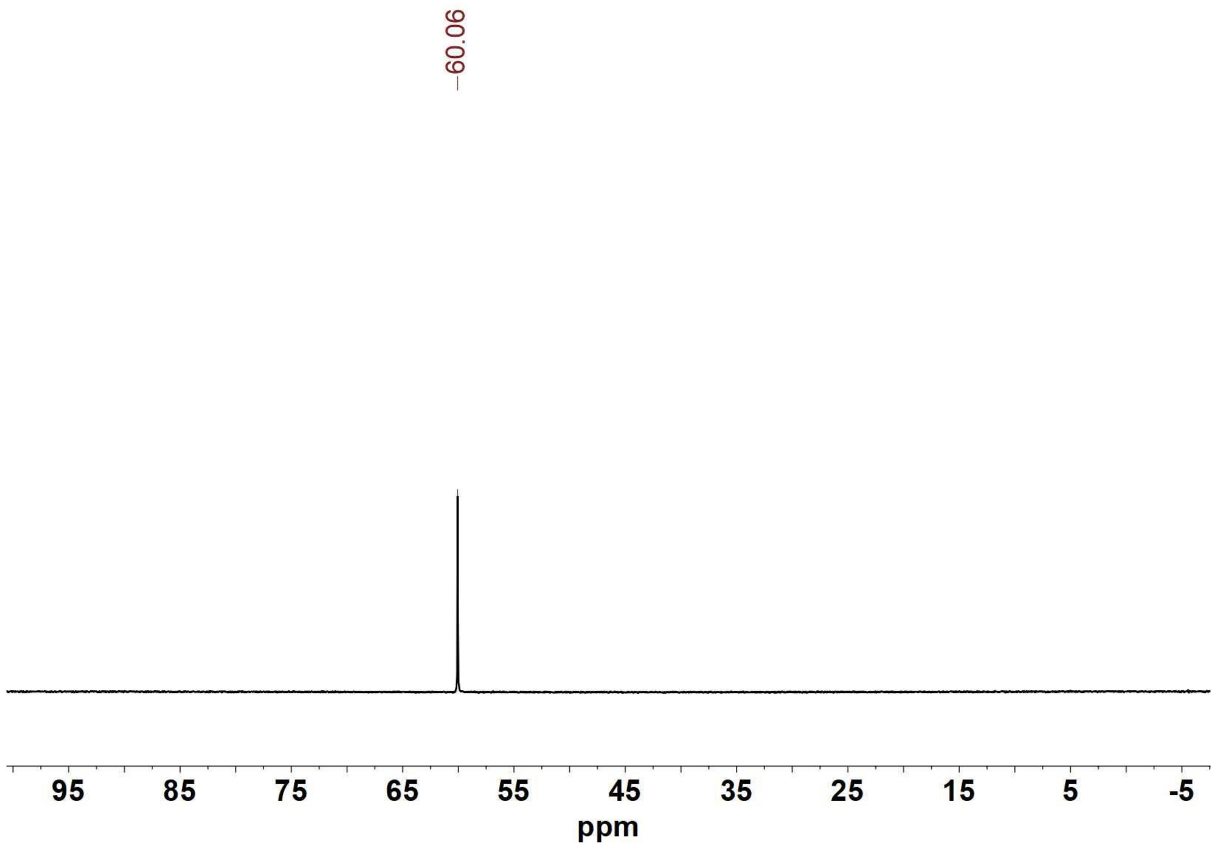

Figure S35. ^{31}P NMR spectrum of $Et_4N[Fe_2[(SCH_2)_2NCH_2CN](CN)(CO)_4PPh_3]$ (Et_4N[**5**]) in CD_2Cl_2 at room temperature.

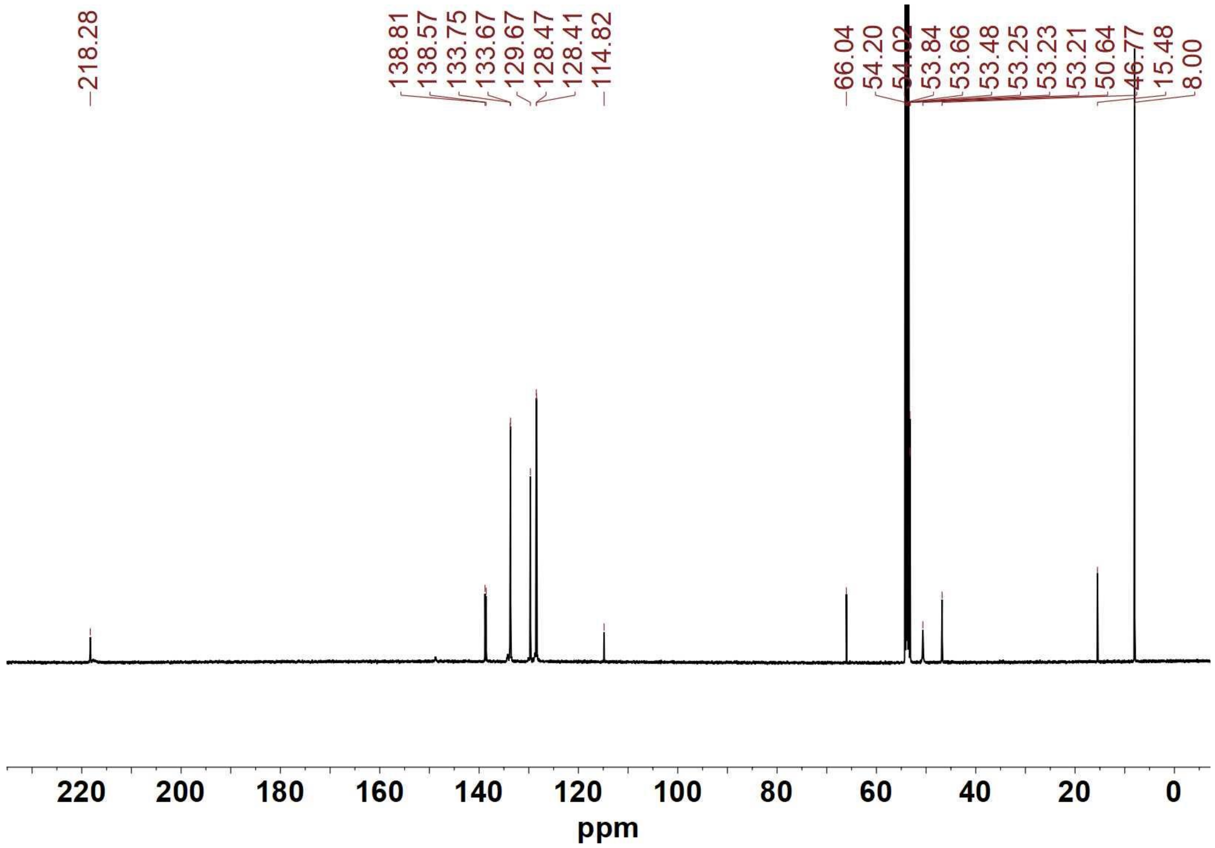

Figure S36. ^{13}C NMR spectrum of $Et_4N[Fe_2[(SCH_2)_2NCH_2CN](CN)(CO)_4(PPh_3)]$ (Et_4N[**5**]) in CD_2Cl_2 at room temperature.

Assignments:
δ 66.04, 15.48 (diethyl ether)

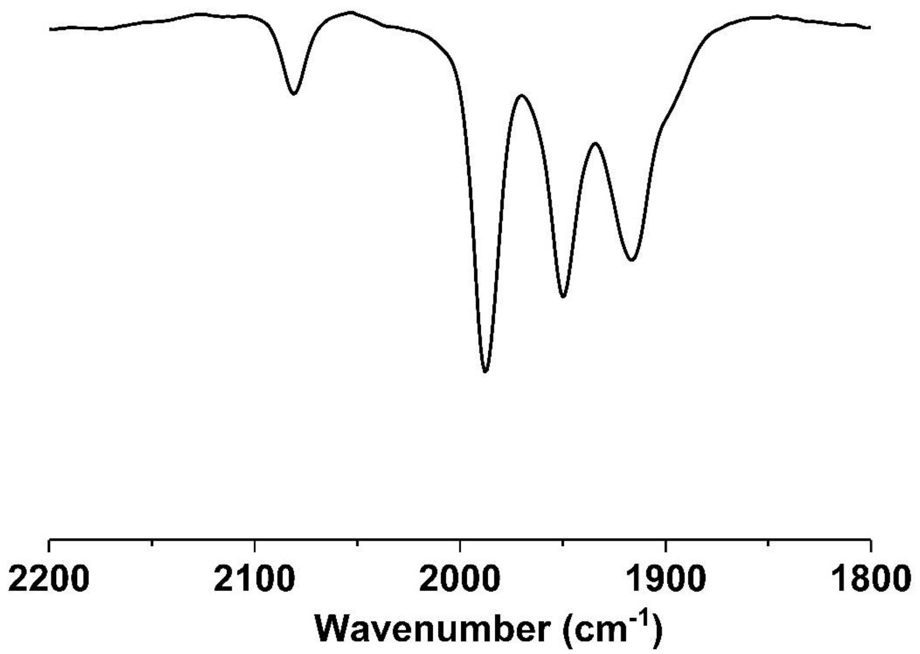

Figure S37. IR spectrum of $Et_4N[Fe_2[(SCH_2)_2NCH_2CN](CN)(CO)_4PPh_3]$ (Et_4N[**5**]) in CH_2Cl_2 solution.

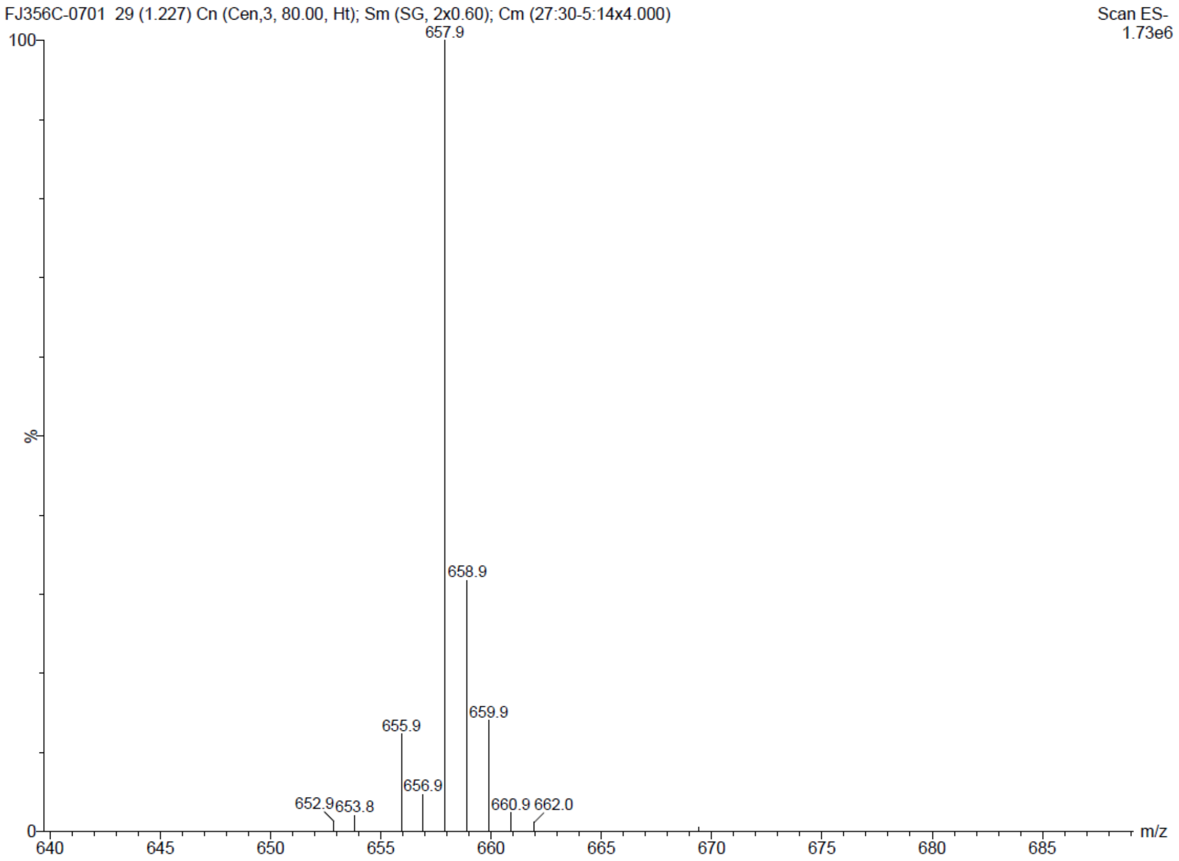

Figure S38. ESI-MS spectrum of $Et_4N[Fe_2[(SCH_2)_2NCH_2CN](CN)(CO)_4PPh_3]$ (Et_4N[**5**]) in CH_2Cl_2 solution.

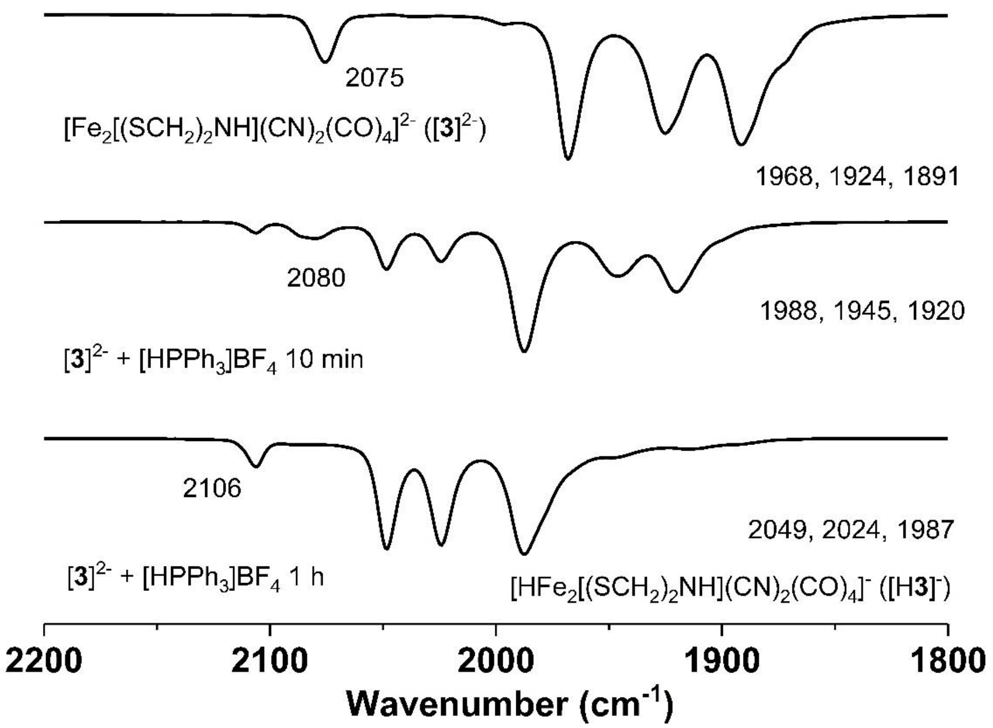

Figure S39. IR spectra of $(Et_4N)_2[Fe_2[(SCH_2)_2NH](CN)_2(CO)_4]$ ($(Et_4N)_2$[**3**]) + $[HPPh_3]BF_4$ in CH_3CN solution.

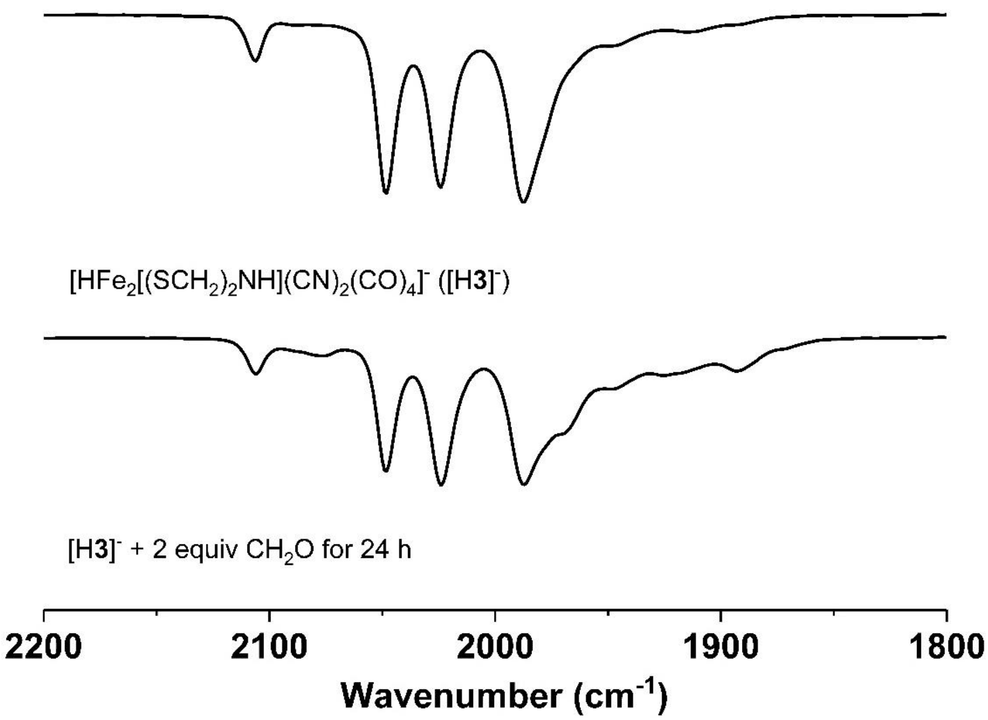

Figure S40. IR spectra of $(Et_4N)[HFe_2[(SCH_2)_2NH](CN)_2(CO)_4]$ ($(Et_4N)[H\mathbf{3}]$) + 2 equiv CH_2O in CH_3CN solution.

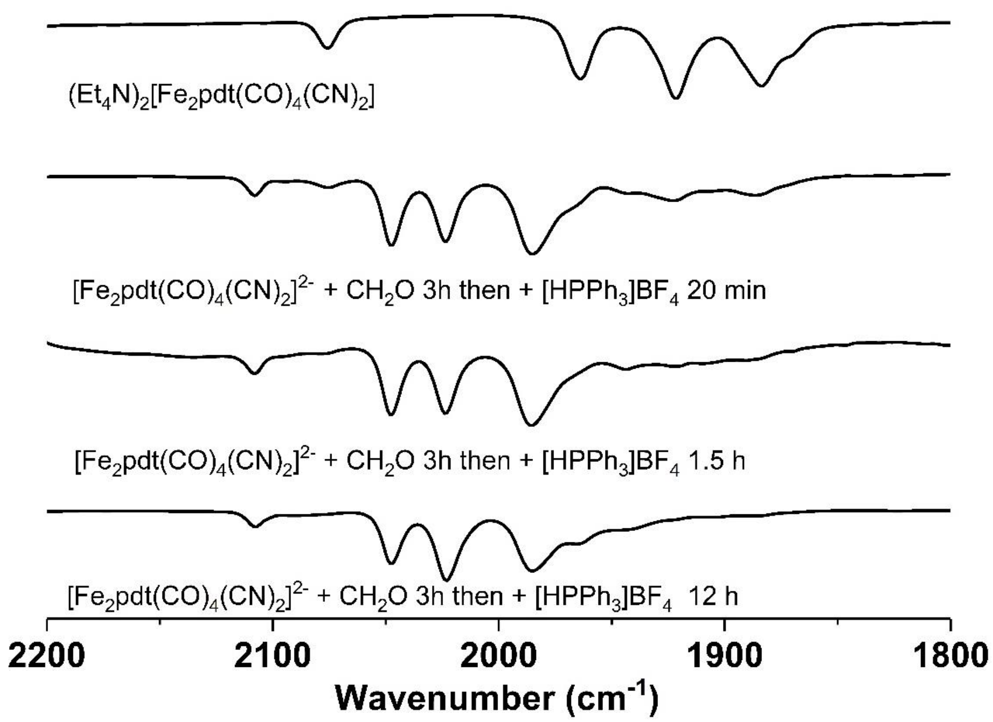

Figure S41. IR spectra for the reaction of $(Et_4N)_2[Fe_2(pdt)(CN)_2(CO)_4]$ with CH_2O and $[HPPh_3]BF_4$ in CH_3CN solution.

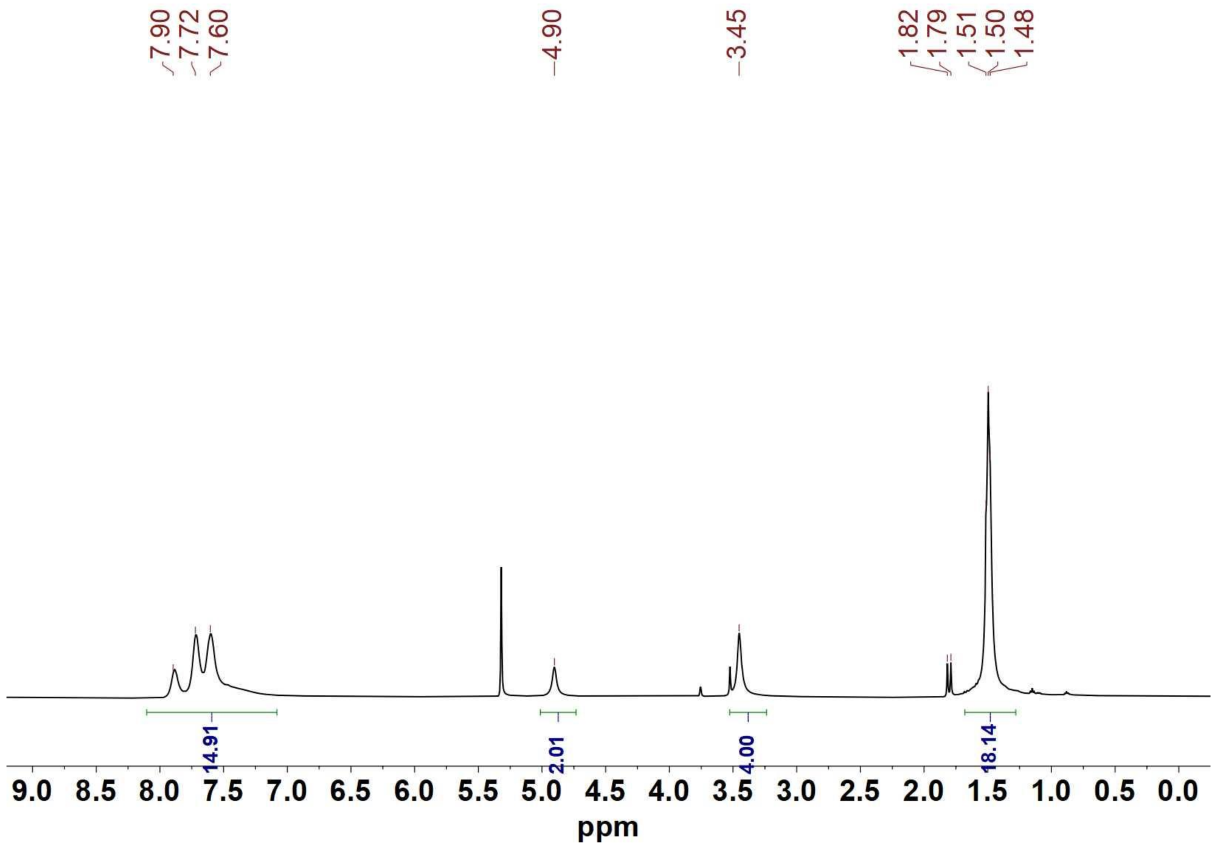

Figure S42. ^{1}H NMR spectrum of $[Fe_2[(SCH_2)_2NCH_2PPh_3](CO)_4(PMe_3)_2]BF_4$ ([**6**]BF_4) in CD_2Cl_2 at room temperature.

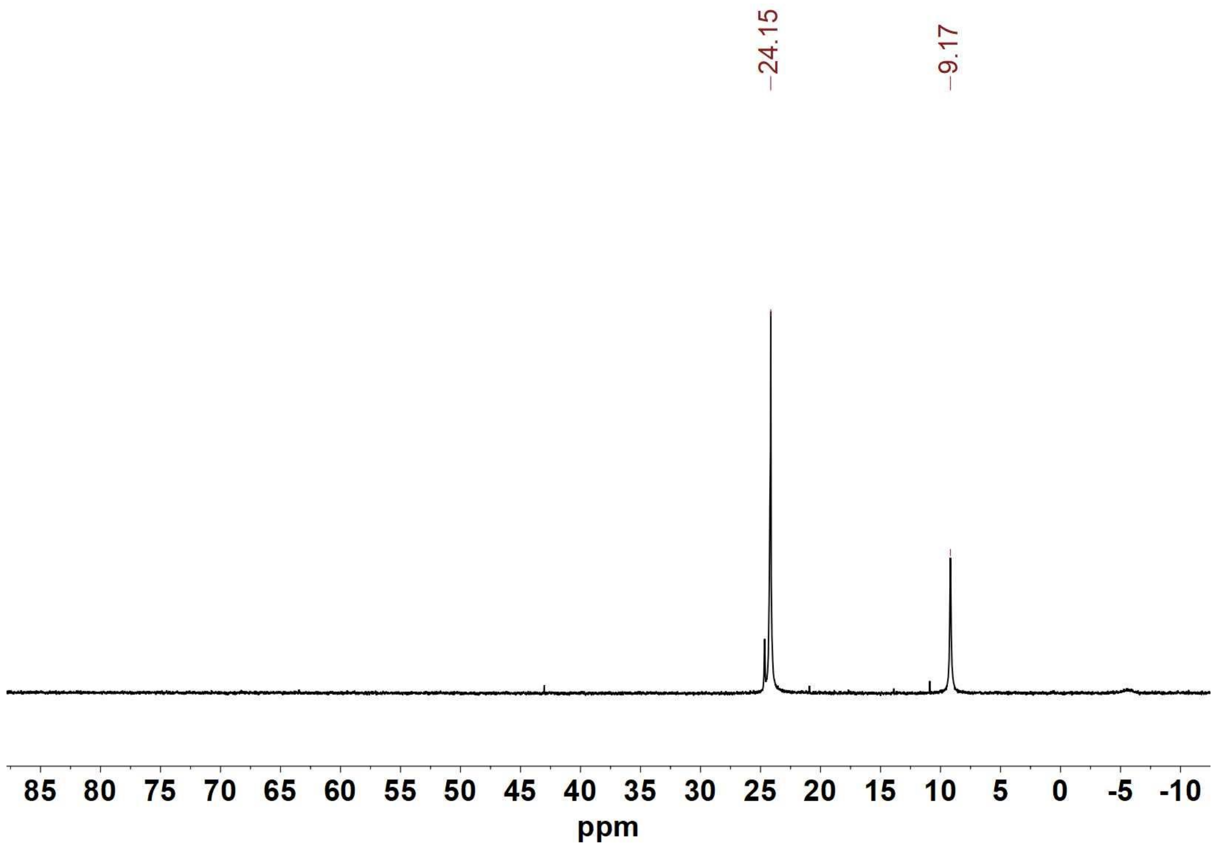

Figure S43. ^{31}P NMR spectrum of $[Fe_2[(SCH_2)_2NCH_2PPh_3](CO)_4(PMe_3)_2]BF_4$ ([**6**]BF_4) in CD_2Cl_2 at room temperature.

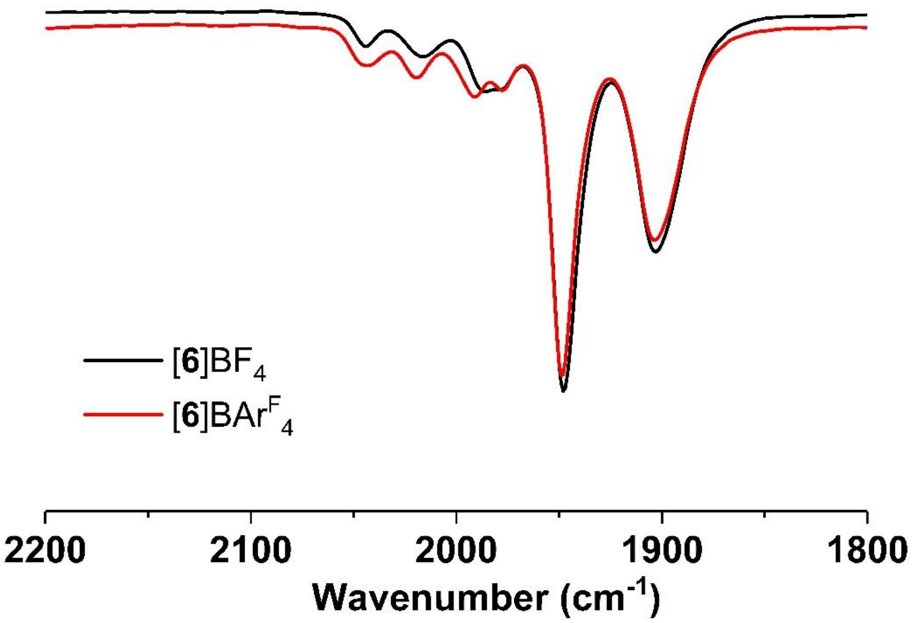

Figure S44. IR spectra of $[Fe_2[(SCH_2)_2NCH_2PPh_3](CO)_4(PMe_3)_2]BF_4$ ([**6**]BF_4) and [**6**]$BAr^F{}_4$ in CH_2Cl_2 solution.

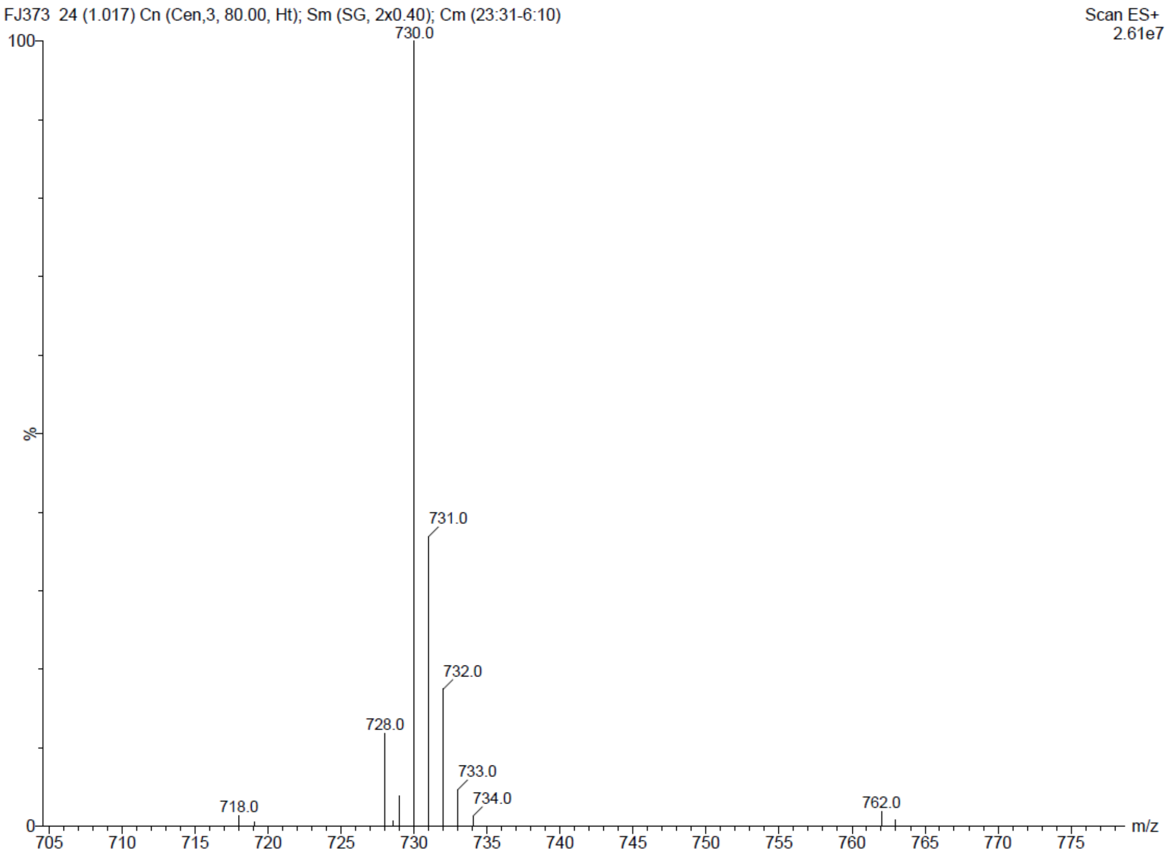

Figure S45. ESI-MS spectrum of $[Fe_2[(SCH_2)_2NCH_2PPh_3](CO)_4(PMe_3)_2]BF_4$ ([**6**]BF_4) in CH_2Cl_2 solution.

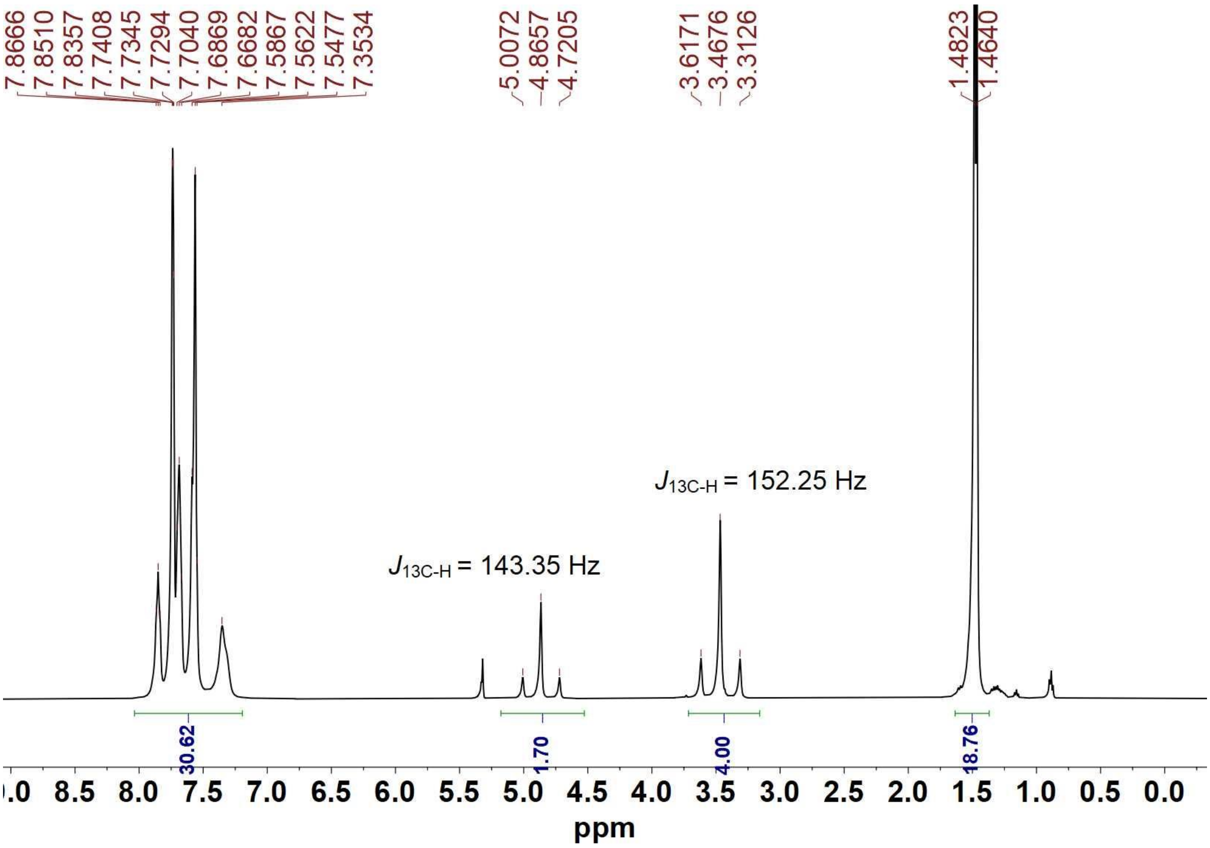

Figure S46. ^{1}H NMR spectrum of ^{13}C labeled $[Fe_2[(SCH_2)_2NCH_2PPh_3](CO)_4(PMe_3)_2]BAr^F{}_4$ ([**6**]$BAr^F{}_4$) in CD_2Cl_2 at room temperature.

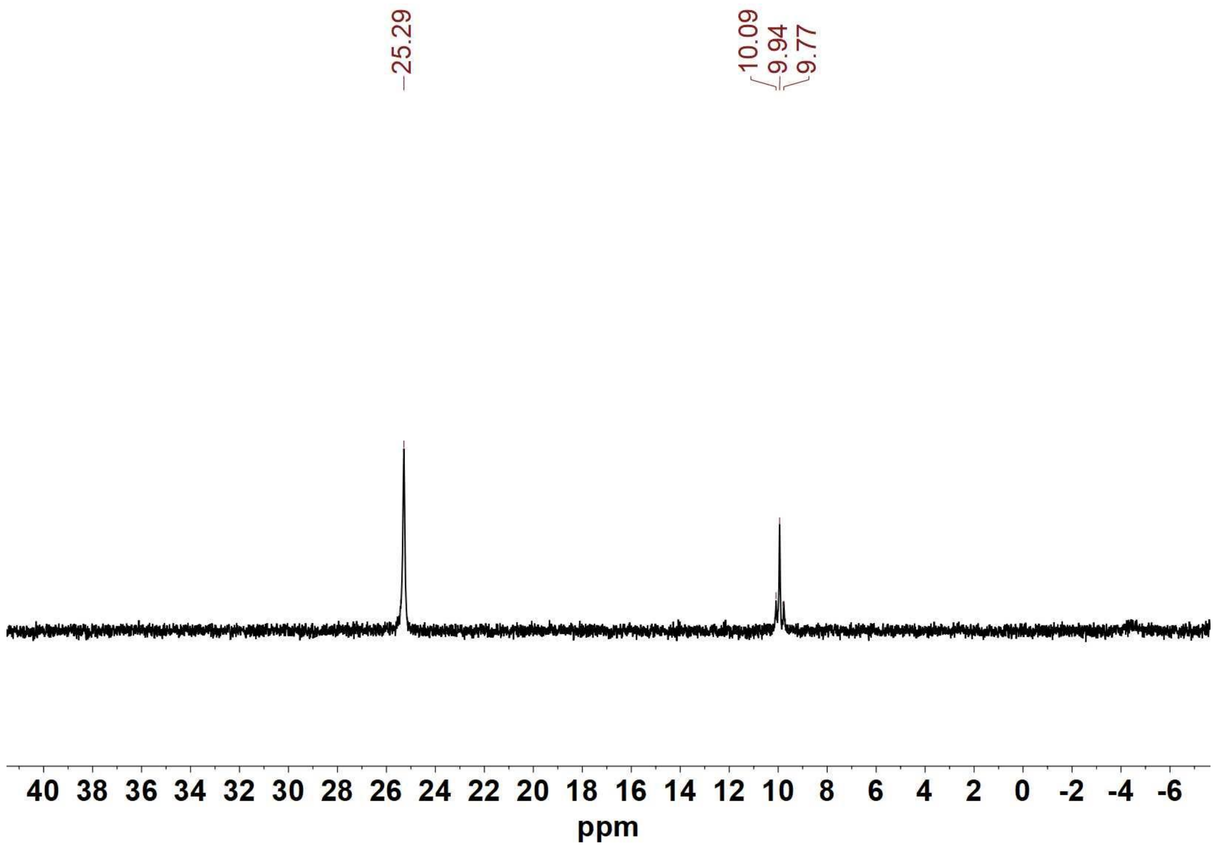

Figure S47. ^{31}P NMR spectrum of ^{13}C labeled $[Fe_2[(SCH_2)_2NCH_2PPh_3](CO)_4(PMe_3)_2]BAr^F_4$ ([**6**]BAr^F_4) in CD_2Cl_2 at room temperature.

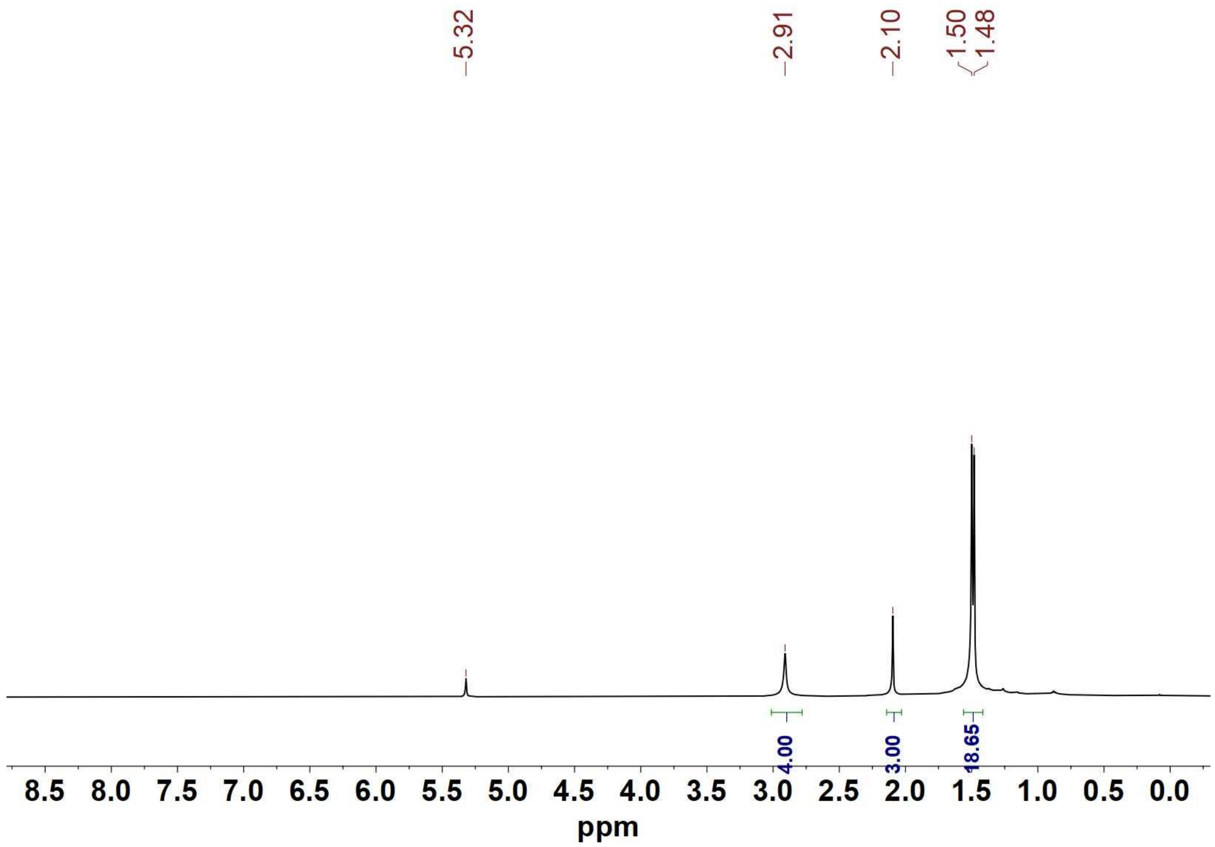

Figure S48. ^{1}H NMR spectrum of $Fe_2[(SCH_2)_2NMe](CO)_4(PMe_3)_2$ in CD_2Cl_2 at room temperature.

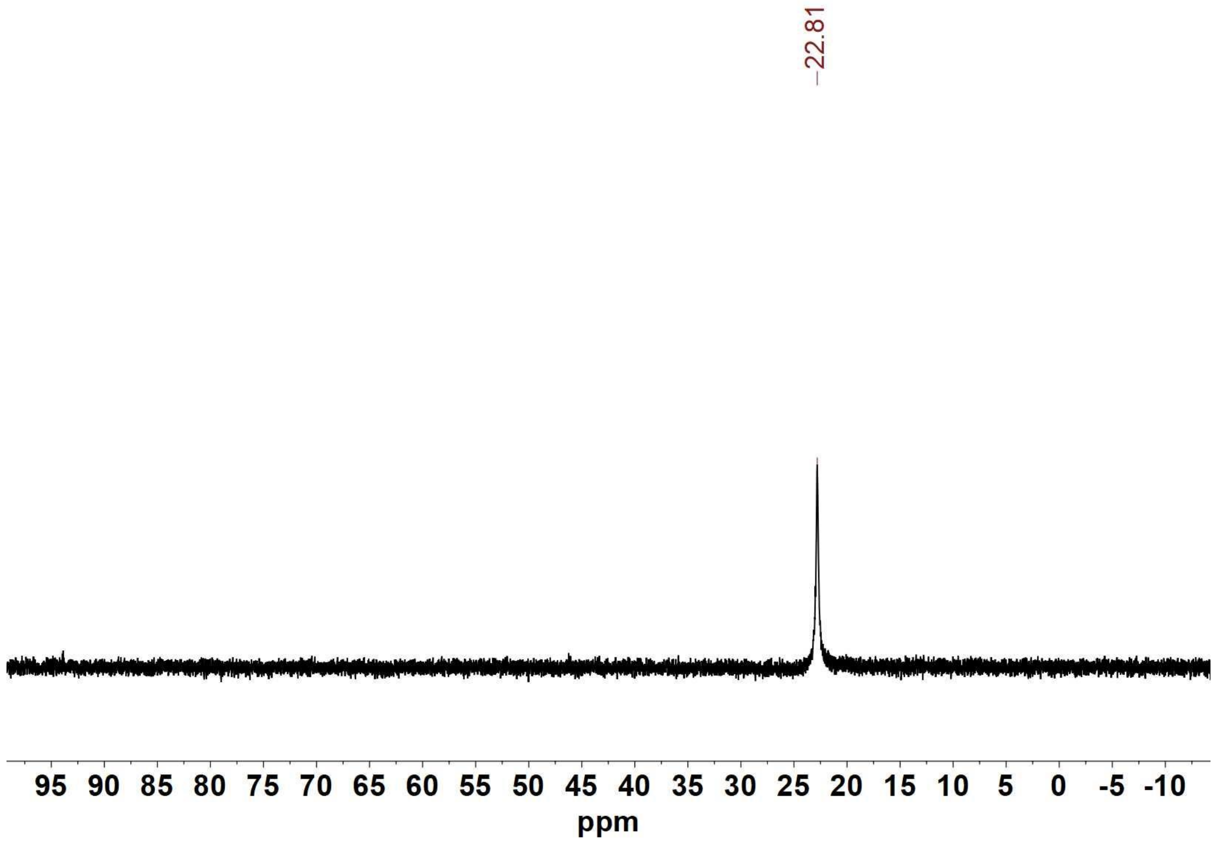

Figure S49. ^{31}P NMR spectrum of $Fe_2[(SCH_2)_2NMe](CO)_4(PMe_3)_2$ in CD_2Cl_2 at room temperature.

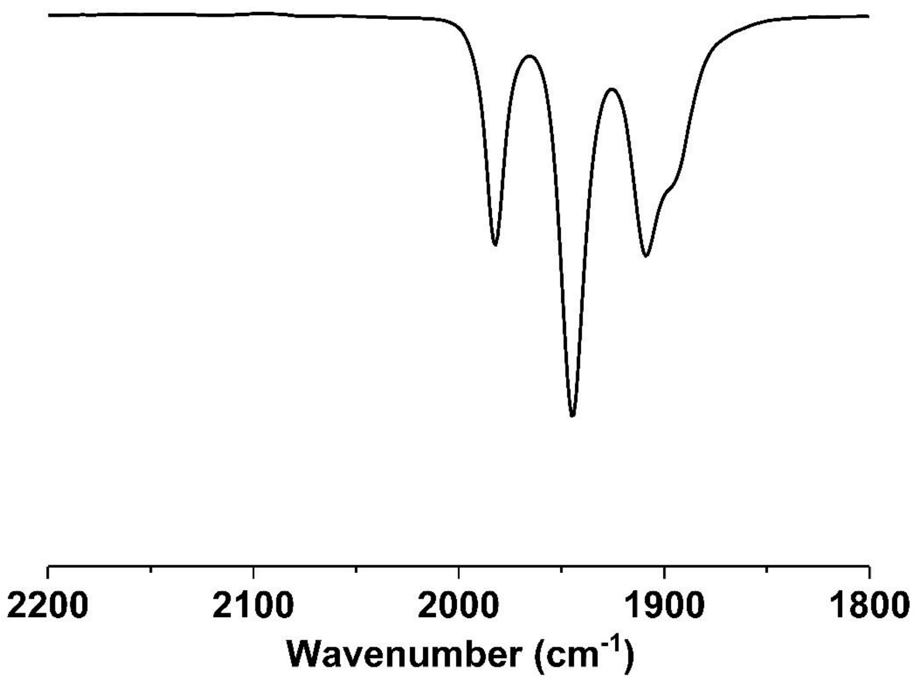

Figure S50. IR spectrum of $Fe_2[(SCH_2)_2NMe](CO)_4(PMe_3)_2$ in CH_2Cl_2 solution.

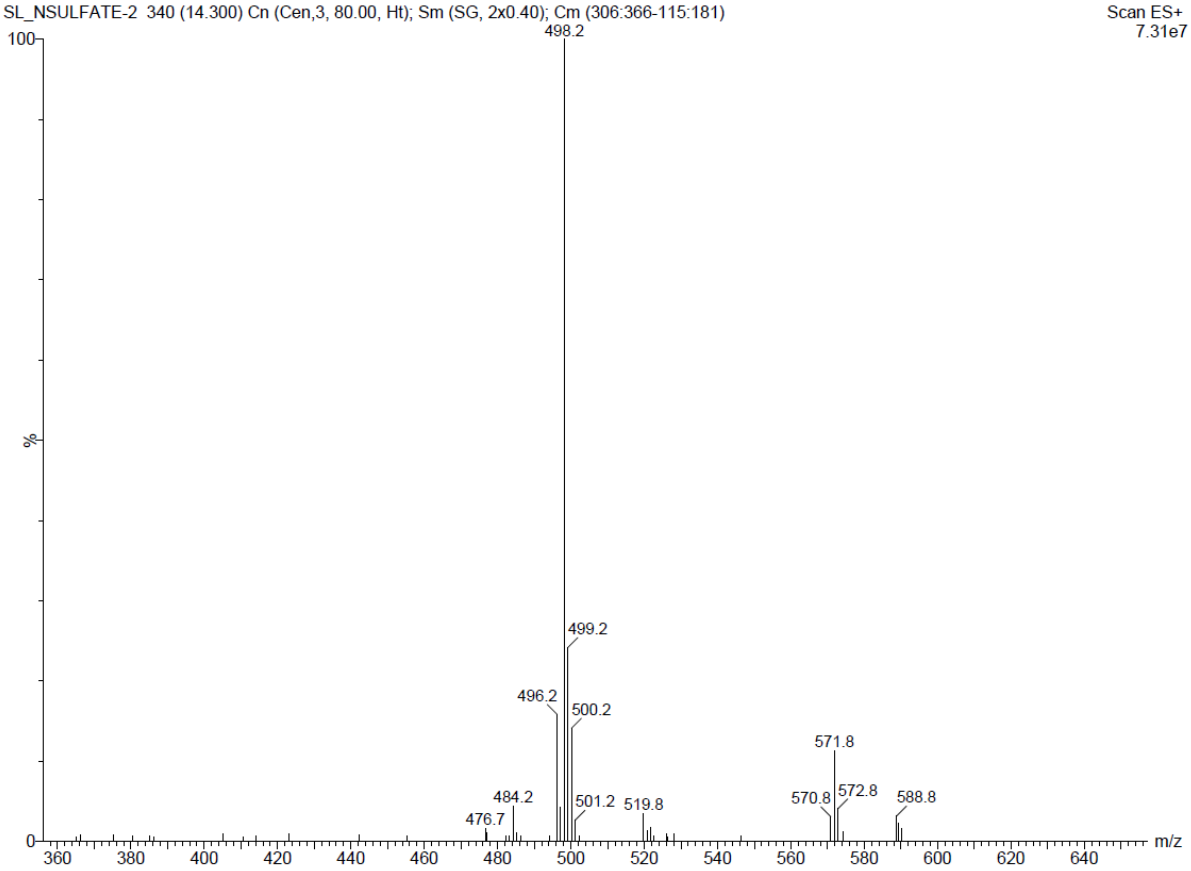

Figure S51. ESI-MS spectrum of $Fe_2[(SCH_2)_2NMe](CO)_4(PMe_3)_2$ in CH_2Cl_2 solution.

Table S1. Crystal Data and Structure Refinements.

Identification code	ed79ks ([**2**]$BAr^F{}_4$)	ed04Ls ([**1**H]BF_4)
Empirical formula	C45 H36 B F24 Fe2 N O4 P2 S2	C12.67 H25.33 B Cl1.33 F4 Fe2 N O4 P2 S2
Formula weight	1359.32	627.51
Temperature	100(2) K	100(2) K
Wavelength	0.71073 Å	0.71073 Å
Crystal system	Triclinic	Triclinic
Space group	P-1	P-1
Unit cell dimensions	a = 13.5275(3) Å<br>b = 14.3475(4) Å<br>c = 16.5656(4) Å	a = 14.0486(2) Å<br>b = 16.9932(3) Å<br>c = 17.3960(3) Å
Volume	2661.82(11) $Å^3$	3759.01(11) $Å^3$
Z	2	6
Density (calculated)	1.696 Mg/m^3	1.663 Mg/m^3
Absorption coefficient	0.811 mm^{-1}	1.643 mm^{-1}
F(000)	1360	1908
Crystal size	0.228 x 0.184 x 0.064 mm^3	0.191 x 0.153 x 0.107 mm^3
Theta range for data collection	2.290 to 28.301°.	2.149 to 28.307°.
Index ranges	-18<=h<=18, -19<=k<=19 -22<=l<=22	-18<=h<=18, -22<=k<=22 -23<=l<=23
Reflections collected	190539	222825
Independent reflections	13211 [R(int) = 0.0312]	18706 [R(int) = 0.0328]
Completeness to theta = 25.242°	99.9 %	99.9 %
Absorption correction	Semi-empirical from equivalents	Semi-empirical from equivalents
Max. and min. transmission	0.7457 and 0.7147	0.7457 and 0.7016
Refinement method	Full-matrix least-squares on F^2	Full-matrix least-squares on F^2
Data / restraints / parameters	13211 / 52 / 743	18706 / 548 / 995
Goodness-of-fit on F^2	1.023	1.050
Final R indices [I>2sigma(I)]	R1 = 0.0348, wR2 = 0.0840	R1 = 0.0213, wR2 = 0.0512
R indices (all data)	R1 = 0.0369, wR2 = 0.0856	R1 = 0.0241, wR2 = 0.0527
Extinction coefficient	n/a	n/a
Largest diff. peak and hole	1.246 and -0.990 $e.Å^{-3}$	0.970 and -0.765 $e.Å^{-3}$

Table S2. Crystal Data and Structure Refinements.

Identification code	ed82Ls (Et_4N[**5**])	ed15Ls ([**6**]BF_4)
Empirical formula	C35 H41 Fe2 N4 O4 P S2	C34.79 H48.44 B Cl0.14 F4 Fe2 N O4.93 P3 S2
Formula weight	788.51	920.04
Temperature	120(2) K	100(2) K
Wavelength	0.71073 Å	0.71073 Å
Crystal system	Monoclinic	Monoclinic
Space group	$P2_1/c$	$P2_1/c$
Unit cell dimensions	a = 16.8201(7) Å<br>b = 17.3607(7) Å<br>c = 13.5621(6) Å	a = 13.5415(3) Å<br>b = 17.0231(4) Å<br>c = 18.4766(4) Å
Volume	3719.3(3) $Å^3$	4173.68(16) $Å^3$
Z	4	4
Density (calculated)	1.408 Mg/m^3	1.464 Mg/m^3
Absorption coefficient	0.978 mm^{-1}	0.977 mm^{-1}
F(000)	1640	1904
Crystal size	0.468 x 0.305 x 0.054 mm^3	0.223 x 0.214 x 0.079 mm^3
Theta range for data collection	1.983 to 26.477°.	2.089 to 27.500°.
Index ranges	-21<=h<=21, -21<=k<=21 -16<=l<=16	-17<=h<=17, -22<=k<=22 -23<=l<=23
Reflections collected	73436	60739
Independent reflections	7636 [R(int) = 0.0605]	9573 [R(int) = 0.0352]
Completeness to theta = 25.242°	99.9 %	99.9 %
Absorption correction	Semi-empirical from equivalents	Semi-empirical from equivalents
Max. and min. transmission	0.7454 and 0.5933	0.7456 and 0.7005
Refinement method	Full-matrix least-squares on F^2	Full-matrix least-squares on F^2
Data / restraints / parameters	7636 / 332 / 542	9573 / 90 / 511
Goodness-of-fit on F^2	1.186	1.103
Final R indices [I>2sigma(I)]	R1 = 0.0600, wR2 = 0.1300	R1 = 0.0317, wR2 = 0.0718
R indices (all data)	R1 = 0.0690, wR2 = 0.1341	R1 = 0.0386, wR2 = 0.0750
Extinction coefficient	n/a	n/a
Largest diff. peak and hole	0.859 and -0.663 e.$Å^{-3}$	0.504 and -0.344 e.$Å^{-3}$

Crystallography

Ed04Ls ([**1**H]BF_4) CCDC: 2104104

Intensity data were collected on a Bruker D8 Venture kappa diffractometer equipped with a Photon-II CPAD detector. An Iμs microfocus Mo source (λ = 0.71073 Å) coupled with a multi-layer mirror monochromator provided the incident beam. The sample was mounted on a 0.3 mm nylon loop with the minimal amount of Paratone-N oil. Data was collected as a series of φ and/or ω scans. Data was collected at 100 K using a cold stream of $N_{2(g)}$. The collection, cell refinement, and integration of intensity data was carried out with the APEX3 software.[1] A multi-scan absorption correction was performed with SADABS[2]. The structure was phased with intrinsic phasing methods using SHELXT[3] and refined with the full-matrix least-squares program SHELXL.[4]

A structural model consisting of three target molecules, three BF_4 anions, and two dichloromethane solvent molecules in the asymmetric unit was developed.

Two of the three BF_4 anions were modeled as disordered over two orientations. Within each anion, all 1,2 and 1,3 distances were restrained to be similar (esd 0.01, 0.02 Å). Rigid-bond restraints (esd 0.004) were imposed on displacement parameters for all disordered sites and similar displacement amplitudes (esd 0.005) were imposed on disordered sites overlapping by less than the sum of van der Waals radii. The site occupancy ratios were allowed to freely refine.

Both dichloromethane solvent molecules were modeled as disordered over two orientations. Within each solvent molecule, all 1,2 and 1,3 distances were restrained to be similar (esd 0.01, 0.02 Å). Similar displacement amplitudes (esd 0.005) were imposed on disordered sites overlapping by less than the sum of van der Waals radii. The site occupancy ratios were allowed to freely refine.

H atom treatment - Methyl H atom positions, R-CH_3, were optimized by rotation about R-C bonds with idealized C-H, R--H and H--H distances. All of the amine H atom positions were located in the difference map; their positions were allowed to freely refine. At convergence, all amine H atoms were in good H- bonding geometries. Remaining H atoms were included as riding idealized contributors. Methyl and amine H atom U's were assigned as 1.5 times U_{eq} of the carrier atom; remaining H atom U's were assigned as 1.2 times carrier U_{eq}.

The -3 5 0 reflection was omitted from the final refinement due to being partially obscured by the beam stop support in some orientations.

Ed15Ls ([**6**]BF_4) CCDC: 2104105

Intensity data were collected on a Bruker D8 Venture kappa diffractometer equipped with a Photon-II CPAD detector. An Iμs microfocus Mo source (λ = 0.71073 Å) coupled with a multi-layer mirror monochromator provided the incident beam. The sample was mounted on a 0.3 mm nylon loop with the minimal amount of Paratone-N oil. Data was collected as a series of φ and/or ω scans. Data was collected at 100 K using a cold stream of $N_{2(g)}$. The collection, cell refinement, and integration of intensity data was carried out with the APEX3 software.[1] A multi-scan absorption correction was performed with SADABS[2]. The structure was phased with intrinsic phasing methods using SHELXT[3] and refined with the full-matrix least-squares program SHELXL.[4]

A structural model consisting of the target molecule, a BF_4 counter ion, and a disordered solvent molecule position in the asymmetric unit was developed.

The solvent molecule position was refined as occupational disorder of diethyl ether and dichloromethane. The dichloromethane occupancy was only approximately seven percent; to maintain a reasonable geometry with such a low occupancy it was refined as an idealized, rigid fragment.[5] Similar displacement amplitudes (esd 0.01) were imposed on disordered sites overlapping by less than the sum of van der Waals radii. The site occupancy ratio was allowed to freely refine.

H atom treatment - Methyl H atom positions, R-CH_3, were optimized by rotation about R-C bonds with idealized C-H, R--H and H--H distances. Remaining H atoms were included as riding idealized contributors. Methyl H atom U's were assigned as 1.5 times U_{eq} of the carrier atom; remaining H atom U's were assigned as 1.2 times carrier U_{eq}.

The 1 1 0, 1 0 0, and 0 1 1 reflections were omitted from the final refinement due to being partially obscured by the beam stop in some orientations. The 6 6 12 reflection was omitted from the final refinement due to being partially obscured by the Cu beam stop in some orientations. The -8 1 3 and -9 1 2 reflections both showed large F_o^2 vs. F_c^2 deviations with F_o^2 being larger than F_c^2. Inspection of individual frame images revealed that in several instances there was a hot pixel on the detector in close enough proximity to the reflection that it may have been included in the integration box. These reflections were omitted from the final refinement.

Ed79ks ([**2**]$BAr^F{}_4$) CCDC: 2104106

Intensity data were collected on a Bruker D8 Venture kappa diffractometer equipped with a Photon-II CPAD detector. An Iμs microfocus Mo source (λ = 0.71073 Å) coupled with a multi-layer mirror monochromator provided the incident beam. The sample was mounted on a 0.3 mm nylon loop with the minimal amount of Paratone-N oil. Data was collected as a series of φ and/or ω scans. Data was collected at 100 K using a cold stream of $N_{2(g)}$. The collection, cell refinement, and integration of intensity data was carried out with the APEX3 software.[1] A multi-scan absorption correction was performed with SADABS[2]. The structure was phased with intrinsic phasing methods using SHELXT[3] and refined with the full-matrix least-squares program SHELXL.[4]

A structural model consisting of the target molecule plus one $BArF_{24}$ anion in the asymmetric unit was developed.

Two of the CF_3 groups on the $BArF_{24}$ anion were modeled as disordered; the C20 group over two orientations and the C29 group over three orientations. Similarity restraints (esd 0.01 Å) were imposed on all disordered C---F bond distances. Within each disordered group, the fluorine displacement parameters were constrained to be the same. The C20 site occupancy ratio was allowed to freely refine. The site occupancies for the three C29 orientations were allowed to freely refine with in the restraint that the total occupancy was 1.000(1).

H atom treatment - Methyl H atom positions, R-CH_3, were optimized by rotation about R-C bonds with idealized C-H, R--H and H--H distances. Remaining H atoms were included as riding idealized contributors. Methyl H atom U's were assigned as 1.5 times U_{eq} of the carrier atom; remaining H atom U's were assigned as 1.2 times carrier U_{eq}.

The -1 1 1 reflection was omitted from the final refinement due to being partially obscured by the beam stop in some orientations.

Ed82Ls (Et_4N[**5**]) CCDC: 2104107
Intensity data were collected on a Bruker D8 Venture kappa diffractometer equipped with a Photon-II CPAD detector. An Iμs microfocus Mo source (λ = 0.71073 Å) coupled with a multi-layer mirror monochromator provided the incident beam. The sample was mounted on a 0.3 mm nylon loop with the minimal amount of Paratone-N oil. Data was collected as a series of φ and/or ω scans. Data was collected at 120 K using a cold stream of $N_{2(g)}$. The collection, cell refinement, and integration of intensity data was carried out with the APEX3 software.[1] A multi-scan absorption correction was performed with SADABS[2]. The structure was phased with intrinsic phasing methods using SHELXT[3] and refined with the full-matrix least-squares program SHELXL.[4]

A structural model consisting of the target molecule plus one tetraethylammonium cation in the asymmetric unit was developed.

The nitrile substituent on the adt bridge was modeled as disordered over two orientations. Similarity restraints (esd 0.01 Å) were imposed on all chemically equivalent bond distances. To maintain a more linear geometry about the nitrile group for the minor orientation, similarity restraints (esd 0.02 Å) were imposed on the distances between the nitrile nitrogen atoms and the CH_2 H atoms. Similar displacement amplitudes were imposed on disordered sites overlapping by less than the sum of van der Waals radii. The site occupancy ratio was allowed to freely refine.

The tetraethylammonium cation was modeled as disordered over two orientations. All 1,2 and 1,3 distances of the cation were restrained to be similar (esd 0.01, 0.04 Å). Similar displacement amplitudes were imposed on disordered sites overlapping by less than the sum of van der Waals radii. The site occupancy ratio was allowed to freely refine.

H atom treatment - Methyl H atom positions, R-CH_3, were optimized by rotation about R-C bonds with idealized C-H, R--H and H--H distances. Remaining H atoms were included as riding idealized contributors. Methyl H atom U's were assigned as 1.5 times U_{eq} of the carrier atom; remaining H atom U's were assigned as 1.2 times carrier U_{eq}.

The -1 1 1 and 0 2 0 reflections were omitted from the final refinement due to being partially obscured by the beam stop in some orientations.

(1) Bruker (2018). APEX3. Bruker AXS, Inc., Madison, Wisconsin, USA.
(2) Krause, L., Herbst-Irmer, R., Sheldrick, G. M. and Stalke, D. *J. Appl. Cryst.*, **2015**, *48*, 3-10.
(3) Sheldrick, G. M. *Acta Cryst*. **2015**, **A**71, 3-8.
(4) Sheldrick, G. M. *Acta Cryst*. **2015**, **C**71, 3-8.
(5) Guzei, I. A. *J. Appl. Cryst.*, **2014**, *47*, 806-809.
